# Trap-assisted circularly polarized organic photodetector

**DOI:** 10.1038/s41467-026-72474-w

**Published:** 2026-04-25

**Authors:** Hongki Kim, Zhuoran Qiao, Marie Houot, Martina Rimmele, Francesco Furlan, Matilde Brunetta, David Reger, Filip Aniés, Rahil Haria, Edoardo Angela, Ding Ding, Michele Conroy, Jessica Wade, Martin Heeney, Artem A. Bakulin, Nicola Gasparini, Matthew J. Fuchter

**Affiliations:** 1 Department of Chemistry, Molecular Science Research Hub, Imperial College London, London, UK; 2 Department of Materials, Imperial College London, Prince Consort Road, London, UK; 3 Department of Chemistry, University College London, 20 Gordon Street, London, UK; 4 Department of Chemistry, Chemistry Research Laboratory, University of Oxford, 12 Mansfield Road, Oxford, UK; 5 King Abdullah University of Science and Technology (KAUST), Thuwal, Saudi Arabia; 6 Centre for Processable Electronics, Imperial College London, London, UK

**Keywords:** Materials for devices, Optical materials and structures, Optical materials, Imaging and sensing

## Abstract

The innovative design of π-conjugated molecules enables the creation of chiral π-systems that selectively interact with circularly polarized light. In circularly polarized organic photodetectors, the use of π-conjugated molecules with highly dissymmetric circularly polarized absorption has been a key strategy for achieving strong differential electrical signals with circularly polarized light. However, factors beyond absorption that influence asymmetric device behavior remain insufficiently explored. In this work, we demonstrate trap-assisted circularly polarized organic photodetectors based on blend systems containing small amounts of achiral acceptors, enabling asymmetric trap filling depending on the handedness of circularly polarized light. Modulated trap dynamics play a crucial role in asymmetric charge extraction, significantly enhancing circularly polarized selectivity. We realize a large-area organic circularly polarized imaging system (4096 pixels) and showcase its potential for real-time visual encryption. This study presents a design strategy for efficient circularly polarized photodetectors with tailored carrier dynamics.

## Abstract (web summary)

Kim et al. report asymmetric trap filling in circularly polarized organic photodetectors, leading to asymmetric charge extraction and enhanced selectivity of circularly polarized light. Large-area circularly polarized imaging system with 4,096 pixels are demonstrated for real-time visual encryption.

## Introduction

The rational design of π-conjugated organic semiconducting materials offers a versatile platform for tuning optoelectronic properties through precise control of molecular structure^1,2^. This alters the transition dipole moments of the molecule, enabling the development of chiral organic semiconductors that possess sizeable electric (*μ*) and magnetic (*m*) transition dipole moments, oriented in such a way that they exhibit a non-zero rotational strength (*R*), given by *R* = |*μ*|·|*m*|cos*θ*^3–5^. A strong *R* enables dissymmetric interactions with right and left-handed circularly polarized (RCP and LCP) light.

When used as the photoactive layer in circularly polarized organic photodetectors (CP-OPDs), chiral organic semiconductors permit electrical detection of CP light^6–29^. This offers exceptional potential for next-generation optoelectronics, particularly for advanced visualization and communication technologies^30–33^. Dissymmetric absorbance (quantified by the *g*-factor, *g*_abs_ = 4*R*/(*μ*² + *m*²)) results in CP-sensitive exciton generation. Subsequent splitting of the excitons and collection of the dissociated free charges at the charge-collection interface leads to a dissymmetric photocurrent. The photocurrent dissymmetry (quantified by *g*_Ph_ = 2(*I*_LCP_ − *I*_RCP_)/(*I*_LCP_ + *I*_RCP_), where *I*_LCP_ and *I*_RCP_ are the photocurrents of the device under LCP and RCP light), is key to the CP selectivity of CP-OPDs.

Traditionally, the main strategy to maximizing *g*_Ph_ in CP-OPDs has focused on designing π-conjugated chiral molecules that have high CP absorption selectivity (i.e. high *g*_abs_) while preserving efficient charge transport. This has driven the preparation of assemblies of organic chiral materials with intrinsically strong *g*_abs_ achieved through supramolecular chirality^11,14,21^, chiral side chains^6,12,13,17,18,22,23^, a combination of both^7,8,15,24,25,27^, or the blending of π-conjugated chiral host molecules and chiral inducers (CIs)^9,10,19,20,26,28^. There has been limited investigation into the factors that contribute to CP-OPD detection selectivity beyond *g*_abs_, particularly the impact of dissymmetric charge carrier dynamics. It has been shown that the sign of *g*_Ph_ can be inverted relative to *g*_abs_ due to the optical penetration depth of CP light in a planar photodiode device^19^. The sign reversal can be attributed to the interplay between the differential optical penetration depth of RCP and LCP photons and the spatial dependence of carrier collection efficiency, which is influenced by the proximity of photogenerated carriers to the charge-collecting contact^22^. This implies that the charge carrier dynamics and optical penetration depth play a significant role in determining *g*_Ph_. While there has been reports of devices where *g*_Ph_ is larger than *g*_abs_^29^, the underlying mechanism of this asymmetry enhancement is presently unclear.

Considering the charge-carrier extraction process, CP-OPDs can differ fundamentally from conventional non-polarized photodetectors due to the variable optical penetration depth depending on the polarization state. Yet the impact of these differences on *g*_Ph_, coupled with the introduction of an acceptor, has not been investigated. In this work, by incorporating a small amount (1 wt%) of non-fullerene small molecule acceptors (NFAs) into a chiral host material, we intentionally create trap states that asymmetrically affect carrier extraction based on the handedness of incident CP light. This trap-induced asymmetry significantly enhances *g*_Ph_, uncovering a previously untapped mechanism for performance improvement. Through a systematic investigation of the interactions between chiral host polymers (CHPs), CIs and acceptors, we enhance the *g*_Ph_ of CP-OPDs to one of the highest values (absolute *g*_Ph_ = 1.19) reported for CP-OPDs, without modifying the *g*_abs_. To our knowledge, this is the highest *g*_Ph_ reported for an organic photodiode operated under low reverse bias (< 2 V) with a uniform active layer, representing a key step toward scalable on-chip implementation of polarization-resolved imaging. Our study provides deep insight into the critical role of charge carrier dynamics, which offers a transformative approach to the design of high-performance CP-OPDs. Finally, we demonstrate a large-area (5 cm × 5 cm) organic CP-imaging system capable of real-time spatiotemporal visualization of light handedness and polarization-dependent pattern encryption. This breakthrough holds the potential to accelerate the development of next-generation optoelectronic devices and advanced technologies in the fields of medicine, biology, and quantum optics.

## Results

### Performance of circularly polarized organic photodetectors

**Fig. 1:**
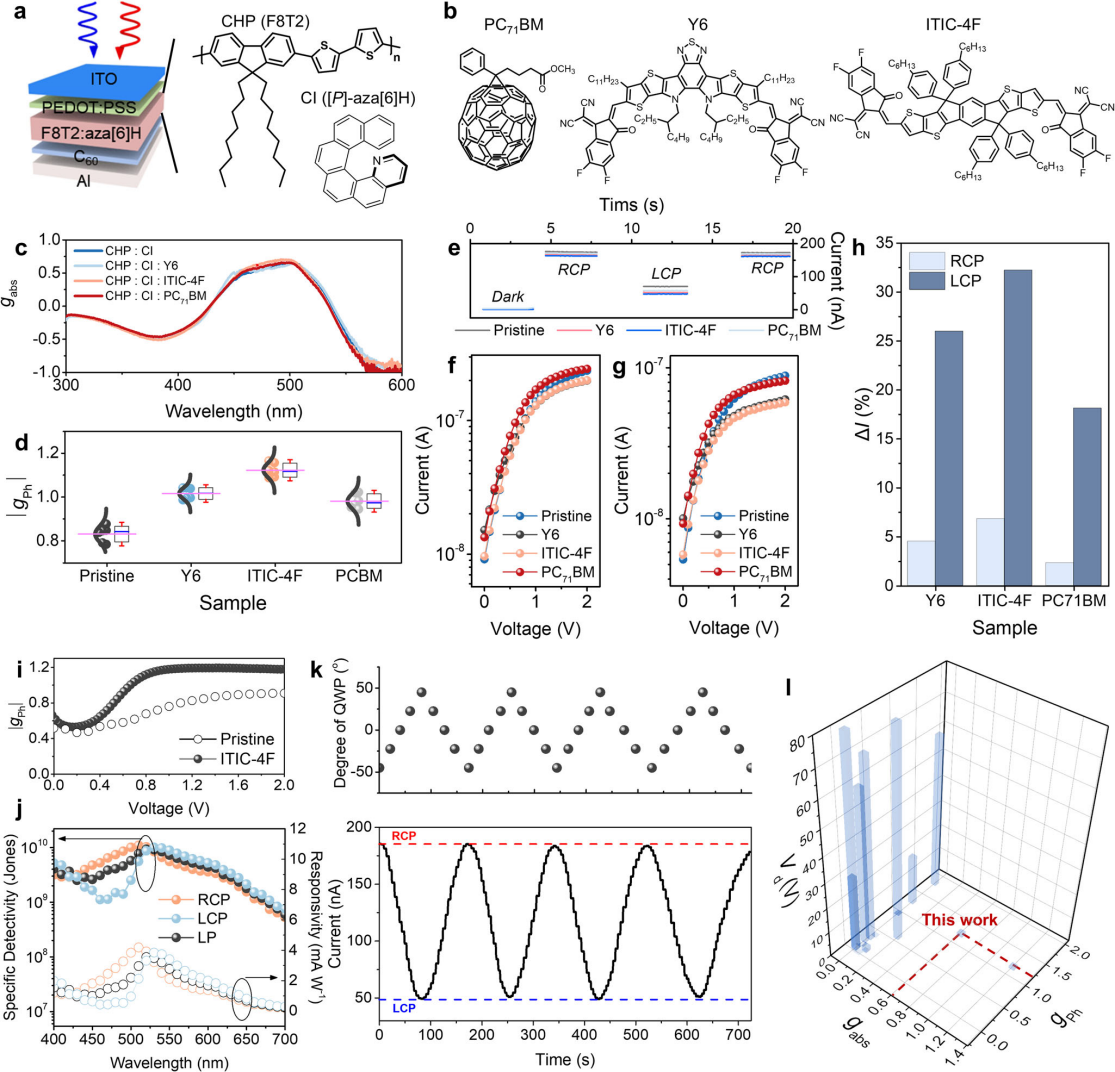
Device characteristics of CP-OPDs. **a** Schematic illustration of device configuration with CHP:CI as an active layer in CP-OPD. **b** Molecular structures of each acceptor. **c** *g*_abs_ spectra of CHP:CI films with the introduction of different acceptors. **d** Statistics of *g*_Ph_ for each type of CP-OPDs under 470 nm CP light (23.6 mW cm^−2^). Box range is defined by the SD of *g*_Ph_ for 6 devices. Red, blue, and pink lines indicate whisker, median line, and mean line, respectively (pristine: median (0.84219), upper (0.84752) and lower quartiles (0.79082), upper (0.87670) and lower whiskers (0.78423), interquartile range (0.05670), no outliers; Y6: median (1.01799), upper (1.04150) and lower quartiles (0.99370), upper (1.04150) and lower whiskers (0.98710), interquartile range (0.04780), no outliers; ITIC-4F: median (1.11730), upper (1.15670) and lower quartiles (1.09170), upper (1.16450) and lower whiskers (1.08780), interquartile range (0.06500), no outliers; PC_71_BM: median (0.97386), upper (1.01910) and lower quartiles (0.95250), upper (1.02180) and lower whiskers (0.94461), interquartile range (0.06660), no outliers). **e** Time-dependent CP light detection characteristics for each type of device. **f**, **g** *I* − *V* curves of each CP-OPDs under RCP (**f**) and LCP illumination (**g**). **h** Photocurrent difference under RCP and LCP compared to those from the pristine device. **i** Voltage-dependent *g*_Ph_ for the pristine and the ITIC-4F-incorporated CP-OPDs. **j** Spectral *R* and D^*^ of ITIC-4F-introduced CP-OPDs under the linear polarized (LP), RCP, and LCP excitation at the reverse bias of 1 V. **k** Sensing of polarized light with full polarization states (bottom), where the polarization state is controlled by adjusting the angle of the quarter-wave plate (QWP) (top). **l** Comparison of this work with previously reported organic-based CP-OPDs in terms of *g*_Ph_, *g*_abs_, and driving voltage (*V*_d_) (see Supplementary Table 2).

As an active layer for CP-OPDs, we employed a CHP:CI blend composed of F8T2 and either [*P*]- or [*M*]-aza[6]H, incorporating 10 wt% of [*P*]- or [*M*]-aza[6]H into the polymer solution. This blend was chosen because our previous work demonstrated that thin films prepared with this ratio exhibited a high *g*_abs_ of 0.7 and excellent film homogeneity, enabling planar CP-OPDs with absolute *g*_Ph_ values approaching 0.8^19^. We used a previously reported device structure of CP-OPDs: indium tin oxide (ITO)/poly(3,4-ethylenedioxythiophene):polystyrene sulfonate (PEDOT:PSS)/CHP:CI/C_60_/Al (Fig. 1a)^19^. To investigate the role of charge dynamics, we selected acceptors of distinct materials classes: Y6 and ITIC-4F (planar non-fullerene acceptors, NFAs), and PC_71_BM (a non-planar fullerene derivative) (Fig. 1b). The planar NFAs are more susceptible to conformational perturbations induced by CIs, which can drive them into chiral configurations, as reported previously^10^. In contrast, the non-planar fullerene derivative possesses greater structural rigidity and is therefore less responsive to such perturbations. These structural distinctions are expected to affect the homogeneity of the acceptors within the chiral blend, leading to variations in the interfacial area within the chiral material. Such variations may, in turn, influence the overall charge dynamics of CP-OPDs.

We incorporated small amounts (1 wt%) of these acceptors into the CHP:CI blend and then studied the impact on CP-OPD performance. Figure 1c demonstrates that the incorporation of a low loading of acceptor does not affect the *g*_abs_ of a CHP:CI blend, as evidenced by the similar intensity and spectral shape of *g*_abs_ compared to the pristine film. The maximum *g*_abs_ (0.7) occurs at *λ* ≈ 500 nm, and the detection wavelength of CP-OPDs was set to 470 nm. The resulting CP-OPDs showed marked differences in *g*_Ph_ depending on the type of incorporated acceptor (Fig. 1d). The average absolute *g*_Ph_ values for pristine, Y6-, ITIC-4F-, and PC_71_BM-incorporated CP-OPDs were 0.83, 1.02, 1.12, and 0.97, respectively, highlighting a substantial enhancement with just 1% acceptor addition, without any alteration to *g*_abs_. Identical trends in *g*_abs_ and *g*_Ph_ were observed when using the opposite enantiomer of CI ([*M*]-aza[6]H) (Supplementary Figs. 1, 2), validating the generality and robustness of our strategy in enhancing CP selectivity of CP-OPDs, independent of the chiral configuration of the CI. This data indicates that *g*_abs_ is not solely responsible for amplifying the selectivity in CP-OPDs. Using grazing-Incidence wide-angle X-Ray scattering (GIWAXS), atomic force microscopy (AFM), and profilometry, we confirmed that the observed differences in CP-OPD performance did not originate from changes in the morphology or thickness of the chiral material (Supplementary Figs. 3–5, Supplementary Table 1), as no noticeable differences were observed between the samples. Bulk and surface morphologies appeared amorphous, as seen through the lack of anisotropic diffraction patterns and flat, featureless surfaces. Film thickness varied by less than 5%, ranging between 220–230 nm. The *I*−*V* curves and time-dependent detection characteristics under CP light (Fig. 1e−g) reveal that the introduction of the acceptor impacts *I*_LCP_, with a much smaller effect on *I*_RCP_ (Fig. 1h). This effect maximizes the difference between *I*_LCP_ and *I*_RCP_, which amplifies *g*_Ph_. The ITIC-4F-incorporated CP-OPD displayed the most pronounced reduction in *I*_LCP_ and achieved the highest *g*_Ph_.

Overall, the best-performing device (with ITIC-4F) gave CP-OPDs with an absolute *g*_Ph_ = 1.19 under a very small reverse bias (< 2 V) and the stable discrimination of CP light (Fig. 1i, Supplementary Fig. 6). We also extracted other figure-of-merit parameters, including spectral responsivity (SR) and detectivity (D^*^), external quantum efficiency (EQE), linear dynamic range (LDR), and rise and fall times (Fig. 1j, Supplementary Fig. 7). All of these parameters showed significantly CP-selective responses; e.g. D^*^ values of 1.01 × 10^10^ Jones and 2.53 × 10^9^ Jones, along with *R* values of 3.84 mAW^−1^ and 0.96 mAW^−1^ under 500 nm RCP and LCP excitation, respectively. Other figures of merit of acceptor-introduced CP-OPDs, such as D^*^, *R*, response speed, and linear dynamic range (LDR), were maintained similarly or slightly decreased compared to those obtained from pristine CP-OPDs without the addition of acceptors (Supplementary Fig. 8). The reductions can be explained by a decrease in charge extraction efficiency caused by traps induced by the acceptor. The pristine CP-OPDs exhibited D^*^ values of 8.42 × 10⁹ Jones and 3.11 × 10⁹ Jones, together with *R* of 4.65 mA W^−1^ and 1.72 mA W^−1^ under 500 nm RCP and LCP illumination, respectively. Compared with the ITIC-4F–introduced CP-OPDs, the pristine devices showed slightly higher EQE, *R*, and faster response times. However, the introduction of ITIC-4F led to a pronounced enhancement in CP selectivity. In addition, the ITIC-4F-introduced CP-OPDs exhibited a slightly higher D^*^ under RCP illumination than the pristine CP-OPDs, which was attributed to their slightly lower noise current. Although a minor trade-off in overall photodetector performance was observed, the incorporation of ITIC-4F resulted in a substantially enhanced polarization-dependent differential response.

We further scanned a wide range of polarization states by controlling the angle of a quarter-wave plate (QWP) (Fig. 1k). The ITIC-4F-incorporated CP-OPDs exhibited an excellent absolute *g*_Ph_ of 1.17 in real-time and also efficiently extracted helicity information from elliptically polarized beams. The long-term stability and reproducibility of the CP-OPDs were also validated by tracking the *g*_Ph_ for 1000 h, during which the devices retained about 90% of their original *g*_Ph_, confirming reliable and consistent operation (Supplementary Fig. 9). Unlike previous studies that relied on extremely high operating voltages^11,12,14,24^ and high *g*_abs_^9^ to achieve efficient, selective CP-OPDs, our method of adding 1% of a non-chiral acceptor provides a distinct and strategic advantage (Fig. 1l, Supplementary Table 2).

### Impact of acceptors on chiral matrix films

**Fig. 2:**
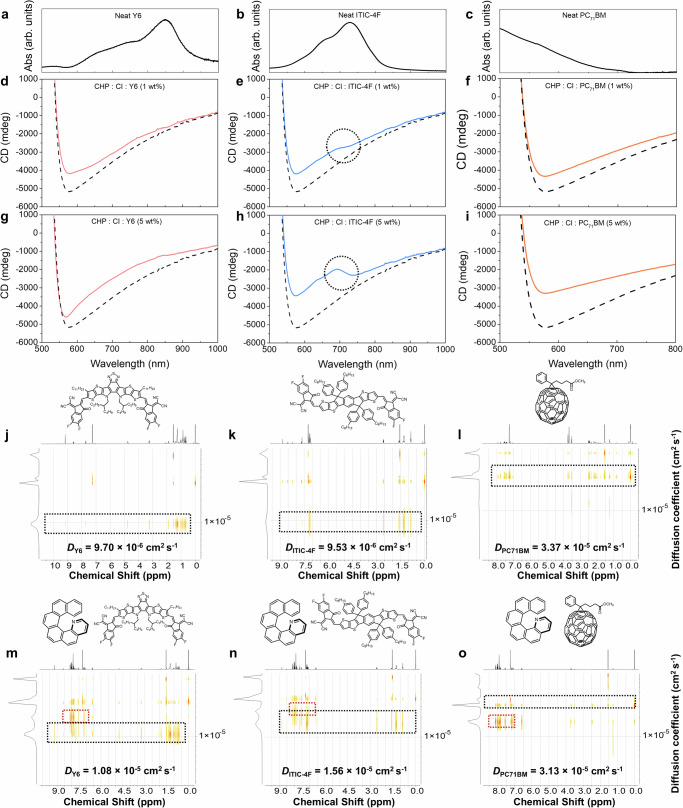
Chiroptical properties of CHP:CI:acceptor films in the near-infrared region and DOSY spectra. **a**–**c** Absorbance of neat Y6 (**a**), ITIC-4F (**b**), and PC_71_BM films (**c**). **d**–**f** CD spectra of CHP:CI with a low-content (1%) introduction of Y6 (**d**), ITIC-4F (**e**), and PC_71_BM (**f**). **g**–**i** CD spectra of CHP:CI with a high-content (5%) introduction of Y6 (**g**), ITIC-4F (**h**), and PC_71_BM (**i**). Dashed black line shows the CD spectrum of pristine CHP:CI film. **j**–**l** Solution-state DOSY spectra of pure Y6 (**j**), ITIC-4F (**k**), and PC_71_BM (**l**). **m**–**o** Solution-state DOSY spectra of mixture Y6:[*P*]-aza[6]H (**m**), ITIC-4F:[*P*]-aza[6]H (**n**), and PC_71_BM:[*P*]-aza[6]H (**o**). In DOSY spectra, dashed black and red lines indicate diffusive peaks corresponding to each acceptor and [*P*]-aza[6]H, respectively.

To uncover the origin of the amplified *g*_ph_, we investigated the chiroptical properties of acceptor-doped CHP:CI thin films in the near-infrared, a region with significant acceptor absorption, but no absorption by the CHP:CI blend (Fig. 2a–i). While no changes were observed in the visible region (Fig. 1c), even at a low loading of 1 wt% ITIC-4F, it exhibited a clear induced CD peak near 700 nm (marked with a dashed circle), which corresponds to the 0–0 transition observed in the absorption spectrum of a neat ITIC-4F film (Fig. 2b, e)^34^. Upon increasing the ITIC-4F content to 5 wt%, a more distinct CD peak appeared in the same spectral region (marked with a dashed circle), suggesting a strong perturbation of ITIC-4F molecules by the CHP:CI film (Fig. 2h)^10,35^. This induced CD feature at the 0–0 transition is more distinct than that observed for Y6-containing films. When Y6 is introduced at a low loading of 1 wt%, no clearly discernible induced CD peak is observed in the wavelength region corresponding to the 0–0 transition (~850 nm) of Y6^36^ (Fig. 2a, d). At a higher loading of 5 wt%, a broad CD feature emerged in the spectral region associated with the 0–1 transition (~750 nm), accompanied by the absence of a clearly discernible CD peak associated with the 0–0 transition (Fig. 2g). Overall, the induced CD features by Y6 remain less distinct than those observed for ITIC-4F at their respective 0–0 transitions. For PC₇₁BM, a flat CD profile with no distinct additional CD peak features attributable to its 0-0 transition near 720 nm^37^ or other absorbance region is detected at either 1 wt% or 5 wt% loading (Fig. 2c, f, i), indicating that the perturbation of PC₇₁BM molecules by the CHP:Cl films is negligible.

It has been previously reported that pure NFA films can be induced to exhibit significant CD when blended with an alternative CI^10^. We observed a similar effect for aza[6]H (Supplementary Fig. 10). Based on these results, we hypothesized that induced CD in acceptors by CIs may, in part, be impacted by the interaction of these two species. The strength of this interaction was examined by using the solution-state diffusion-ordered nuclear magnetic resonance (NMR) spectroscopy (DOSY) (Fig. 2j−o). Comparing the diffusion coefficient (*D*) of the pure acceptor with *D* of the acceptor:CI blend should reveal whether the two species interact^38^, through a change in their effective hydrodynamic radius. For pure [*P*]-aza[6]H, a *D*_aza_ of 2.05 × 10^−5^ cm^2^ s^−1^ was determined from the DOSY spectrum (Supplementary Fig. 11). When comparing the spectrum of pure Y6 with a 1:1 Y6:[*P*]-aza[6]H blend (Fig. 2j, m), it was observed that *D*_Y6_ changes slightly from 9.70 × 10^−6^ cm^2^ s^−1^ in the pure Y6 to 10.8 × 10^−6^ cm^2^ s^−1^ in the Y6:[*P*]-aza[6]H blend. The same effect but much more evident was observed for ITIC-4F (Fig. 2k, n): *D*_ITIC-4F_ increased from 9.53 × 10^−6^ cm^2^ s^−1^ in pure ITIC-4F to 15.6 × 10^−6^ cm^2^ s^−1^ in the blend with [*P*]-aza[6]H. This suggests that the interaction between [*P*]-aza[6]H and ITIC-4F is more intense than the interaction between [*P*]-aza[6]H and Y6, and that aza[6]H better breaks up acceptor aggregates in solution. PC_71_BM was found to diffuse much faster than the NFAs (Fig. 2l), owing to the smaller hydrodynamic radius and better solvation (i.e. less aggregation). When the CI is added (Fig. 2o), the *D*_PC71BM_ does not change appreciably (*D*_PC71BM_ = 3.37 × 10^−5^ cm^2^ s^−1^ in the pure PC_71_BM, *D*_PC71BM_ = 3.13 × 10^−5^ cm^2^ s^−1^ in the blend with [*P*]-aza[6]H). While care should be taken in extrapolating a direct interaction in solution to induced chiroptical effects in CHP:CI thin films, it would seem the diffusion data follow the trend we observe in induced CD.

To elucidate the miscibility trend of the multicomponent system, contact angle measurements were performed to extract surface free energies (*γ*) and estimate miscibility using the Flory–Huggins framework. Surface free energies were calculated from contact angles measured with water, ethylene glycol (EG), and diiodomethane (DIM) as probe liquids (Supplementary Figs. 12, 13), and the corresponding Flory–Huggins interaction parameters (*χ*) were derived to assess the miscibility between the F8T2 host polymer (or F8T2:[*P*]-aza[6]H) and the [*P*]-aza[6]H:acceptor (or acceptor) systems. The extracted Flory–Huggins interaction parameters follow the order [*P*]-aza[6]H:ITIC-4F (or ITIC-4F) < [*P*]-aza[6]H:Y6 (or Y6) < [*P*]-aza[6]H:PC_71_BM (or PC_71_BM), indicating that the [*P*]-aza[6]H:ITIC-4F (or ITIC-4F) exhibits the strongest miscibility with the F8T2 (or F8T2:[*P*]-aza[6]H) among the investigated acceptors. This trend provides information on the general miscibility behavior between the host F8T2 polymer (or F8T2:[*P*]-aza[6]H) and the [*P*]-aza[6]H:acceptor (or acceptor) system containing all three components.

### Asymmetric trap filling and carrier extraction

We further investigated differences in acceptor distribution in CHP:CI thin films using time-of-flight secondary ion mass spectrometry (ToF-SIMS, Supplementary Fig. 14). The distribution of PC_71_BM exhibited less homogeneity in the blend than the NFAs, which maintained a uniform distribution. CI also exhibited a uniform distribution within the blend (Supplementary Fig. 15). Taken together with the DOSY and miscibility studies, it would seem that the inter-mixing between acceptors and CHP:CI is favorable in the order of ITIC-4F > Y6 > PC_71_BM, which aligns with the *g*_Ph_ trend observed in CP-OPDs. Such a correlation suggests that the acceptors form small islands in the blend with different interfacial areas, which impacts the overall charge dynamics of CP-OPDs. Given the difficulty in forming a continuous charge transfer pathway at low acceptor loading, and their deeper lowest unoccupied molecular orbital (LUMO) levels compared to the CHP (Supplementary Fig. 16), we hypothesized that the acceptor does not form a continuous electron transport network, but instead acts as a trap site that modifies the charge extraction process. This was confirmed by space-charge-limited current (SCLC) measurements on electron-only devices with the introduction of acceptors, showing that the addition of 1 wt% acceptors increased the electron trap density (Supplementary Fig. 17).

**Fig. 3:**
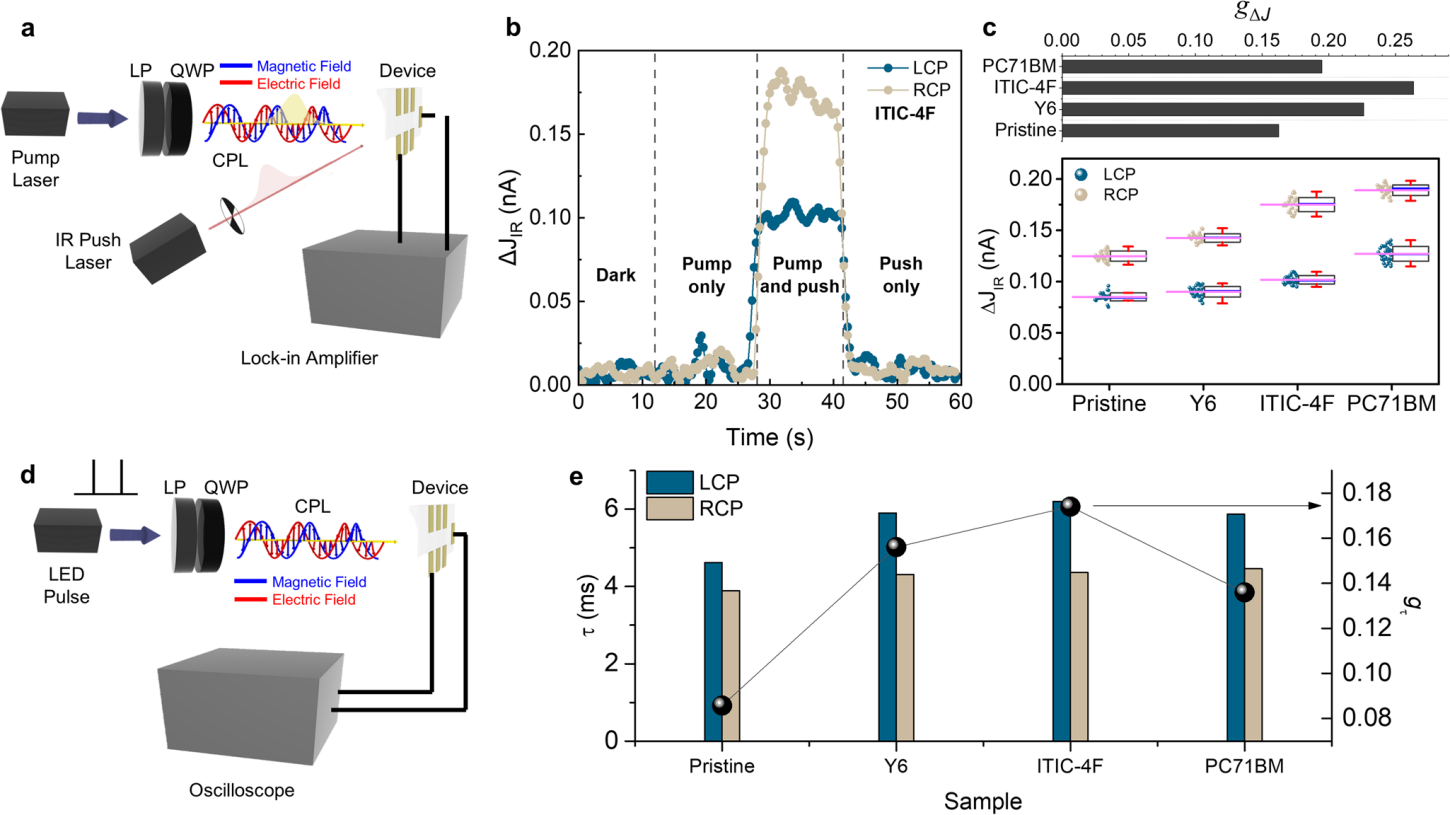
Spectroscopic analysis for CP-OPDs. **a** Schematic illustration of the experimental setup for the PPPc measurement. **b** Δ*J*_IR_ responses in polarization-dependent cw-PPPc measurement for the ITIC-4F introduced CP-OPD as a function of time. **c** Polarization-dependent difference in Δ*J*_IR_ and *g*_Δ*J*_, defined by (Δ*J*_IR,RCP_ − Δ*J*_IR,LCP_)/(Δ*J*_IR,RCP_ + Δ*J*_IR,LCP_) for CP-OPDs. Box range in the box chart is defined by the SD of Δ*J*_IR_ for 31 data points. Red, blue, and pink lines indicate whisker, median line, and mean line, respectively (Under RCP; pristine: median (0.12568), upper (0.12955) and lower quartiles (0.12199), upper (0.13418) and lower whiskers (0.11641), interquartile range (0.00756), no outliers; Y6: median (0.14294), upper (0.14529) and lower quartiles (0.13929), upper (0.15193) and lower whiskers (0.13541), interquartile range (0.00600), no outliers; ITIC-4F: median (0.17671), upper (0.18144) and lower quartiles (0.17157), upper (0.18777) and lower whiskers (0.16332), interquartile range (0.00987), no outliers; PC_71_BM: median (0.19079), upper (0.19281) and lower quartiles (0.18488), upper (0.19818) and lower whiskers (0.17877), interquartile range (0.00793), no outliers), (Under LCP; pristine: median (0.08477), upper (0.08780) and lower quartiles (0.08362), upper (0.09585) and lower whiskers (0.07521), interquartile range (0.00418), no outliers; Y6: median (0.08989), upper (0.09198) and lower quartiles (0.08562), upper (0.09811) and lower whiskers (0.07871), interquartile range (0.00636), no outliers; ITIC-4F: median (0.10125), upper (0.10397) and lower quartiles (0.09905), upper (0.10945) and lower whiskers (0.09493), interquartile range (0.00492), no outliers; PC_71_BM: median (0.12575), upper (0.13024) and lower quartiles (0.12105), upper (0.14026) and lower whiskers (0.11488), interquartile range (0.00919), no outliers). **d** Schematic illustration of the experimental setup for TPC measurement under CP LED (470 nm). **e** The polarization-dependent *τ* for each type of CP-OPDs without the acceptor or with the introduction of acceptors (left axis) and *g*_τ_, defined by (*τ*_R_ − *τ*_L_)/(*τ*_R_ + *τ*_L_) (right axis).

To test this scenario, continuous-wave (cw) visible pump-IR push-photocurrent (PPPc)^39^ was used to directly observe trapped charge carriers in CP-OPDs (Fig. 3a). In the PPPc setup, the device under a 2 V reverse bias was exposed to a circularly polarized pump beam (405 nm), generating free carriers, which give a reference photocurrent (*J*_Pump_), as well as trapped charges. Due to the low loading, acceptor molecules act as trap sites, and carriers localized on acceptor show IR absorption^40^. IR push beam (1550 nm) illuminating the sample is selectively absorbed by trapped charges, and excess energy provided by IR photons de-traps the carriers, generating additional current (Δ*J*_IR_), which is proportional to trapped carrier concentration. Figure 3b, Supplementary Fig. 18 compare the concentrations of trapped carriers created by light with LCP and RCP polarizations. The detectable Δ*J*_IR_ is only observed when both pump and push beams are applied. This confirms that Δ*J*_IR_ results from the combined action of the pump and of the push beam, which re-excites charge carriers trapped on the acceptor^41^. Two key observations emerge from the PPPc study: 1) the concentration of trapped carriers (given by Δ*J*_IR_) is higher under a specific polarization state, 2) CP-OPDs with acceptors exhibit a distinctively larger polarization sensitivity (i.e., Δ*J*_IR,RCP_ − Δ*J*_IR,LCP_), than pristine CP-OPDs without acceptors. The introduction of ITIC-4F produces the greatest polarization-dependent difference in Δ*J*_IR_, compared to CP-OPDs with other acceptors (Fig. 3c). The dissymmetric factor for Δ*J*_IR_ (*g*_Δ*J*_), defined by (Δ*J*_IR,RCP_ − Δ*J*_IR,LCP_)/(Δ*J*_IR,RCP_ + Δ*J*_IR,LCP_) reveals that polarization-dependent asymmetric depopulation of trap sites is strongest in the order of ITIC-4F > Y6 > PC_71_BM, which closely correlates with device CP selectivity (Fig. 1d). Based on these findings, we propose a dissymmetric charge carrier trapping model: carrier trapping occurs more frequently under a specific polarization state of CP light (based on the handedness of the CHP:CI blend), and this trapping is most pronounced in ITIC-4F-based devices. These results suggest that trap dynamics, which become dissymmetric depending on the handedness of CP light and the chiral blend layer, play a key role in the behavior of CP-OPDs.

We further investigated carrier dynamics through polarization-dependent transient photocurrent (TPC) measurements (under 470 nm) to probe charge-carrier photogeneration and extraction dynamics (Fig. 3d). As shown in Supplementary Fig. 19, all devices exhibited a faster current decay under RCP excitation and a slower decay under LCP excitation. The fitting parameters for the TPC decay curves are summarized in Supplementary Table 3, with the lifetimes depending on the handedness of CP light (*τ*_R_, *τ*_L_) and the dissymmetric factor for the lifetime (*g*_*τ*_), defined by (*τ*_R_ − *τ*_L_)/(*τ*_R_ + *τ*_L_), shown in Fig. 3e. *τ*_R_ remained relatively constant across different acceptors, while *τ*_L_ showed significant variation depending on the acceptor type. This supports our assignment that carrier extraction dynamics are more strongly influenced under a specific polarization state of CP light, with charge extraction under LCP light being most suppressed for the CP-OPD with ITIC-4F. The *g*_*τ*_ of 1.74 × 10^−1^ (ITIC-4F), 1.56 × 10^−1^ (Y6), 1.36 × 10^−1^ (PC_71_BM), and 8.58 × 10^−2^ (pristine) directly reflects the trend observed in *g*_Ph_ from CP-OPDs. These TPC results also verify the dissymmetric charge carrier dynamics and are consistent with the PPPc measurements. To ensure the reliability of the observed trend, we also conducted TPC measurements using the opposite enantiomer of aza[6]H incorporated into a chiral matrix (CHP:[*M*]-aza[6]H). As a result, the response to polarization was reversed upon using the opposite enantiomer, while the same trend was consistently observed upon addition of the acceptor (Supplementary Fig. 20).

### Underlying mechanism

**Fig. 4:**
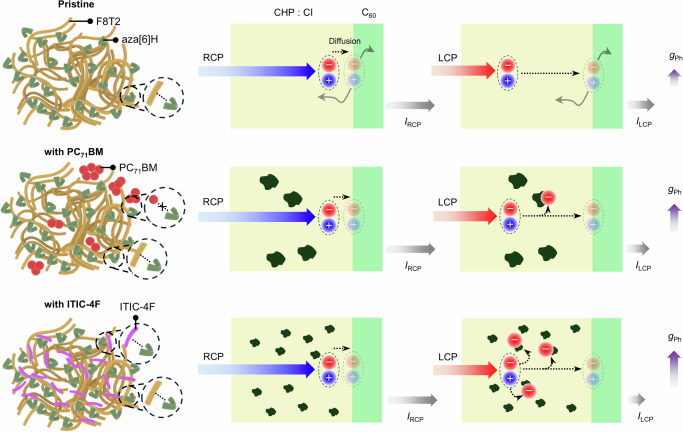
Proposed mechanism for trap-assisted CP-OPDs. RCP light penetrates deeper near the charge collection interface (C_60_), facilitating exciton dissociation and photocurrent compared to LCP light (top). Adding a small amount of chiral non-active acceptor (PC_71_BM) results in confined and larger islands within a chiral material, where LCP excitation causes excitons to dissociate and electrons to trap before reaching the interface, reducing photocurrent. This scenario is less affected under RCP excitation, increasing overall *g*_Ph_ (middle). This effect is further strengthened by incorporating a strong chiral-active acceptor (ITIC-4F), which promotes inter-mixing with a chiral material, increases interfacial trapping, and further boosts *g*_Ph_ (bottom).

Figure 4 outlines our proposed mechanism of trap-assisted CP-OPDs. ITIC-4F provides optimal inter-mixing in the CHP:CI blends, which leads to form finer islands in CHP:CI blends. In contrast, PC_71_BM gives larger, more confined islands within the chiral material. The optical penetration depth of CP light, which can differ by up to a factor of ~ 2 at a wavelength of 470 nm depending on the CP handedness (Supplementary Fig. 21). Under RCP excitation, the generated excitons travel a shorter distance to reach the diode bilayer interface (C₆₀), resulting in a higher photocurrent. In contrast, under LCP excitation, excitons must traverse a longer distance to the interface, leading to a lower photocurrent compared to RCP excitation. This difference gives rise to differential electrical signals in CP-OPDs with *g*_Ph_ opposite in sign to *g*_abs_^19^. When acceptors are incorporated, they introduce trap sites that further modulate carrier dynamics. Under RCP excitation, carriers experience fewer trapping events due to the shorter transport path, whereas under LCP excitation, the longer path increases the probability of carrier trapping. This interpretation is consistent with our device-level observations, including the *I*–*V* curves (Fig. 1f, g), time-dependent real-time CP selectivity (Fig. 1e), and the spectral EQE, *R*, and D^*^ (Fig. 1j, Supplementary Figs. 7, 8) as well as results obtained using the opposite enantiomer of CI ([*M*]-aza[6]H) (Supplementary Fig. 2). In both enantiomer cases, introducing the acceptor more strongly influences the device response under one specific polarization state, while having less effect under the opposite polarization state, providing strong support for the proposed mechanism. These trapping events further reduce the photocurrent compared to the case without acceptors added. This differential effect increases *g*_Ph_, with ITIC-4F demonstrating the largest *g*_Ph_. We assume that Y6 forms slightly larger islands than ITIC-4F within the CHP:CI blends. This reduces the dissymmetric trapping effect and results in a slightly smaller *g*_Ph_. To further validate this mechanism, we minimized the effect of the optical penetration depth of CP light by using a thinner active layer (Supplementary Fig. 22). Acceptors play a less significant role in CP-OPDs with significantly reduced chiral layer thickness (Supplementary Fig. 23). We investigated CP-OPDs with a higher loading of acceptor (5 wt%) (Supplementary Fig. 24), and observe no significant amplification of *g*_Ph_ compared to the case with low (1 wt%) loading. When examining the dependence of photocurrent (*I*_Ph_) on the incident light intensity (*P*_In_) for CP-OPDs with a higher loading of acceptor (5 wt%), we observed that the exponential factor *α* in the power-law relation (*I*_Ph_ ∝ *P*_In_^α^) approached 1, compared to the low loading case (1 wt%) (Supplementary Fig. 25). Ideally, *α* should equal to 1 when all photogenerated charge carriers are efficiently collected before recombination occurs^42^. These results indicate that CP-OPDs with a higher acceptor content exhibit more ideal charge carrier collection behavior: charge carriers are more efficiently extracted before recombination occurs. This suggests that the increased concentration of acceptors facilitates the formation of a continuous carrier transport pathway, alleviating the trapping effect^43,44^. Overall, these results provide compelling evidence that the enhanced *g*_ph_ in CP-OPDs with added acceptors (1% wt) is primarily driven by asymmetric charge carrier trapping in combination with the polarization-dependent optical penetration depth. Meanwhile, the enhancement of *g*_Ph_ was minor under zero-bias conditions (Fig. 1i). This behavior can be attributed to limited exciton dissociation and charge extraction under zero bias, arising from the bilayer device architecture consisting of a thick chiral active layer and a C₆₀ layer. In this configuration, the built-in electric field is mainly localized at the active layer/C₆₀ interface, thereby restricting exciton dissociation and carrier extraction within the bulk of the active layer. As a result, carrier transport is expected to be strongly suppressed under zero bias, reducing the carrier flux and the probability of electrons encountering trap sites. Consequently, trapping events may occur less frequently, weakening the asymmetric trapping effect and leading to a much reduced impact of acceptor addition on CP selectivity.

### Large-area organic CP-imager

**Fig. 5:**
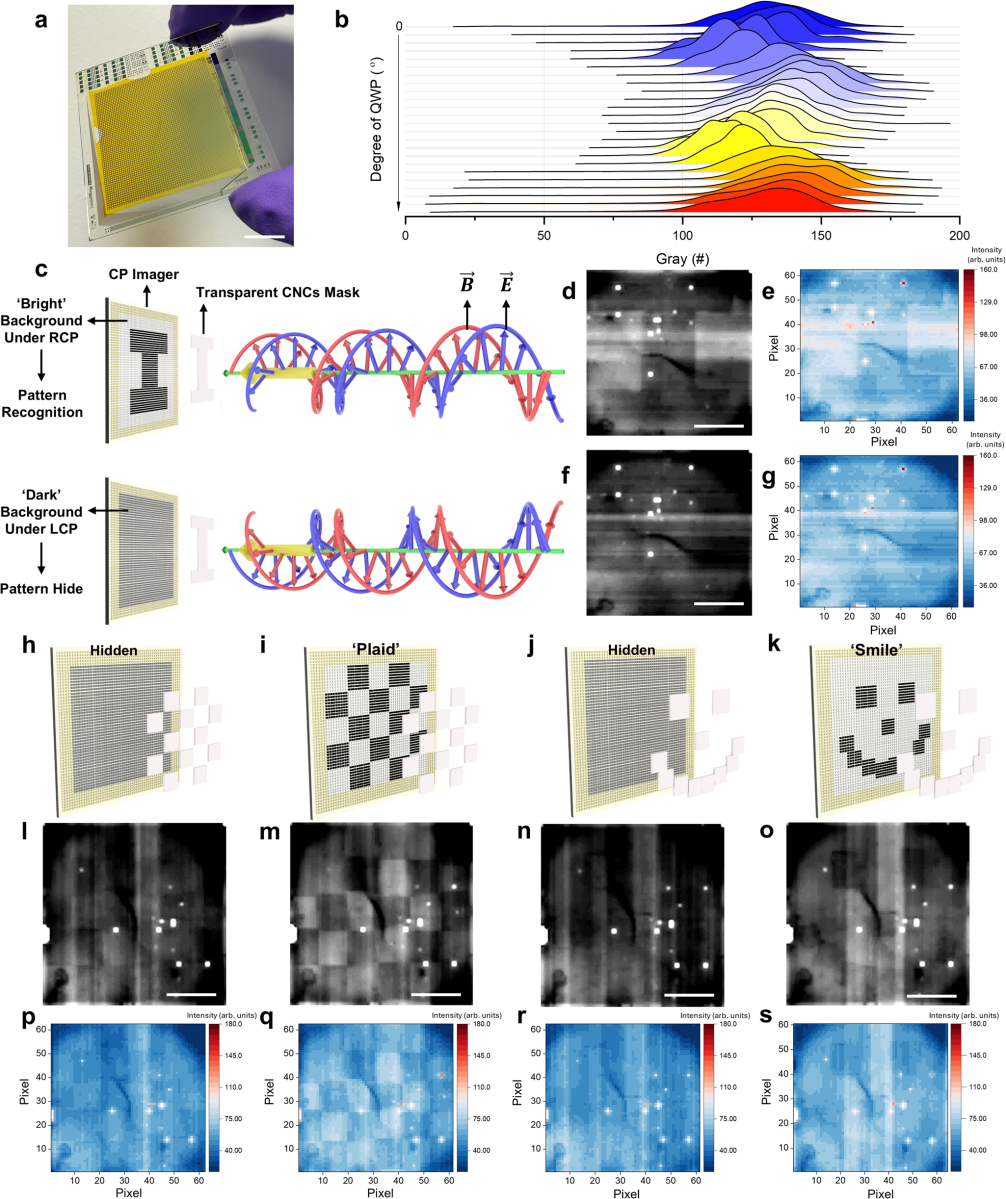
Large-area organic CP-imager. **a** Photograph of a 64 × 64 pixel CP-imager (5 × 5 cm). **b** Grayscale histograms with full polarization states. **c** Schematic of CNCs mask-assisted encryption. **d–g** Scanned ‘I’-pattern images and heatmaps under RCP (**d**, **e**) or LCP light (**f**, **g**). **h–k** Hidden/recognized ‘plaid’ and ‘smile’ patterns under opposite polarizations under LCP (**h**, **j**) or RCP light (**i**, **k**). **l–o** Corresponding scanned images by the CP-imager under LCP (**l**, **n**) or RCP light (**m**, **o**). **p**–**s** Grayscale heatmaps highlighting hidden and revealed patterns under LCP (**p**, **r**) or RCP light (**q**, **s**). Inset scale bars in all scanned images indicate 1 cm.

Having achieved the generation of highly sensitive CP-OPDs, we sought to showcase their practical applicability by fabricating a large-area organic CP-imaging system with 4096 pixels (64 × 64) and capturing real-time images with a software-controlled multipixel imaging acquisition system (Fig. 5a). Real-time detection of light trajectory and handedness was demonstrated by illuminating the CP-imager with polarized light through a specially designed mask. As the helicity of the incident light was modulated, the CP-imager successfully produced real-time spatiotemporal imaging, visualized as contrast variations with full polarization states from 0° to 345° (Supplementary Figs. 26, 27). The conversion of light handedness into visual contrast was evident, with the extracted grayscale values showing distinct mean variations depending on light handednesses (Fig. 5b). The prototype large-area organic CP-imaging system was further applied to pattern encryption using a transparent cellulose nanocrystals (CNCs) film (Fig. 5c, Supplementary Fig. 28). As the CNCs film with a naturally left-handed twist transmit less RCP than LCP light, patterned regions appeared darker under RCP illumination, while uncovered background areas appear bright due to the CP imager’s higher photocurrent response, enabling clear pattern recognition (Fig. 5d, e). Under LCP illumination, however, the background region (not covered by the CNC film) produced lower photocurrent, rendering the background dark and effectively encrypting the pattern (Fig. 5f, g). Depending on the polarization state of the incident light, patterns such as plaid or smiley face were either concealed or revealed (Fig. 5h–k). Scanned images from the CP-imaging system and extracted heatmaps from scanned images clearly showed selective pattern recognition exclusively under specific polarization states (Fig. 5l–s). This proof-of-concept highlights the applicability for encrypted optical communication enabled by direct readout from an all-organic, large-area CP-imaging system.

## Discussion

Until now, the crucial role of charge transport in the dissymmetric response of CP-OPDs has been largely overlooked. Through a detailed investigation spanning materials science and advanced spectroscopy, we reveal that asymmetric trap dynamics play a pivotal role in amplifying *g*_Ph_ in CP-OPDs. Our findings introduce an entirely new approach for designing high-performance CP-OPDs based on tuned charge transport, paving the way for advanced chiral optoelectronics. The resulting CP-OPDs incorporating ITIC-4F demonstrated exceptional sensitivity to CP light, achieving an absolute *g*_Ph_ of 1.19 under reverse bias, one of the highest values ever reported for CP-OPDs. We showcase this high-level sensitivity in a large-area organic CP-imaging with 4096 pixels, which enables real-time spatiotemporal visualization of light handedness and CNCs-assisted pattern encryption. These results suggest our approach to high-performance CP-OPDs holds potential in advancing quantum cryptography and optical communication technologies.

## Methods

### Fabrication of chiral matrix films and photodetectors

F8T2 polymer was purchased from Solaris Chem. To prepare chiral aza[6]H^45^, A solution of 1-aza[6]H precursor (5.4 g, 10.0 mmol) in toluene was treated with (Ph_3_P)₂PdCl₂ (1.8 g, 2.5 mmol), Ph₃P (1.3 g, 5.0 mmol), and (Me₃Sn)₂ (4.8 g, 14.5 mmol), then stirred at 110 °C for 24 h. After cooling, the mixture was concentrated, diluted with Et₂O, washed with saturated NaHCO₃, then brine, dried (Na₂SO₄), filtered, concentrated, and purified by silica gel chromatography. Prepared 1-aza[6]H was separated using preparative chiral high-performance liquid chromatography. Solvents were purchased from Sigma-Aldrich, NFAs, and PC_71_BM were purchased from 1-Material. Indium tin oxide (ITO, 15 Ω sq^−1^) was pre-patterned on 12 mm × 12 mm glass substrates. The substrates underwent a cleaning process involving sonication in acetone for 15 min, followed by isopropanol and a 15 min UV ozone treatment. To prepare chiral CHP:CI films, 25 µL of F8T2 solution in toluene (20 mg ml^−1^) with a 10 wt% loading ratio of [*P*]-aza[6]H was spread on the ITO glass substrate, and spin-coating was performed at 800 rpm for 60 s, followed by 1200 rpm for 5 s. The spin-coated film was annealed at 140 °C for 10 min on a hotplate. To prepare acceptor-introduced chiral CHP:CI films, a specific amount of acceptors was introduced into the CHP:CI solution. To fabricate CP-OPDs, a PEDOT:PSS dispersion (Clevios™ P VP Al 4083) was deposited on the ITO through spin-coating at 5000 rpm for 40 s, followed by annealing at 150 °C for 30 min. CHP:CI films were fabricated by spin-coating onto PEDOT:PSS-coated ITO glass as described above. Then, a 12 nm thick layer of C_60_ and 100 nm thick layer of Al were thermally evaporated under vacuum (< 10^–6^ mbar) in sequence to form an electrode (active area of 0.045 cm^2^). A thin-film transistor (TFT) array chip on an a-Si substrate was purchased from LinkZill to fabricate an imager device. A PEDOT:PSS dispersion (Clevios™ P VP Al 4083) was deposited on the TFT array chip substrate through spin-coating at 5000 rpm for 40 s, followed by annealing at 150 °C for 15 min. A chiral material solution (F8T2:[*P*]-aza[6]H blend with 1 wt% ITIC-4F) was filtered by a 0.45 µm PTFE filter and then spin-coated on the TFT array chip at 1300 rpm for 60 s, followed by annealing at 140 °C for 10 min. Then, a 14 nm thick layer of C_60_ and 100 nm thick layer of Al was thermally evaporated under vacuum (< 10^–6^ mbar). An additional Ag electrode (100 nm) was deposited on top to suppress oxidation of the device.

### Film and device characterization

Keithley 4200 Source-Measure unit (scan rate 25 mV s^−1^) was used to record the *I*–*V* curves. Noise power spectral density was measured by a Keithley 4200 Source Measurement unit and a digital oscilloscope (Siglent, SDS6054A) coupled with a high-speed current amplifier (Femto, DHPCA-100) to provide a variable gain. Responsivity was measured in ambient air using an integrated system from a Quantum Design PVE300 at a modulation frequency of 90 Hz after signal calibration with a Si reference photodiode. For linear dynamic range measurements, a 470 nm continuous LED was pulsed by a function generator (ThorLabs DC2200) and focused onto CP-OPDs. To attenuate the light intensity, neutral density filters were placed between the device and the lamp. Transient photocurrent data were obtained by using a digital oscilloscope (RIGOL DS4024) with a 470 nm LED as the excitation source and a pulse duration of 190 fs. Repetition frequency was set to 5 kHz. Dynamic characterizations (rise and fall times) were performed using a digital oscilloscope (Siglent, SDS6054A). The CP-OPDs were illuminated with a 470 nm LED driven by a function generator (ThorLabs DC2200). For the determination of the rise and fall time a 0.5 kHz square wave pulse was applied to the LED using the function generator. The atomic force microscopy (AFM) images were obtained using an Agilent Technologies Keysight 5500 SPM in tapping mode. Grazing-Incidence Wide-Angle X-Ray Scattering (GIWAXS) were obtained using a Xenocs Xeuss 3.0 SAXS/WAXS system equipped with an auxiliary Cu K*α* Genix3D microfocus X-ray (1.54 Å) source and an Eiger2 4 M detector. Films were prepared by spin-coating the chiral material solution onto silicon substrates. Data analysis and image processing were conducted in XSACT 2.0. ToF-SIMS measurements were conducted on glass/organic thin film samples. The samples were depth profiled using an IONTOF ToF-SIMS V instrument. A 25 keV Bi^+3^ ion beam in HCBM was used to analyze the sample. The samples were sputtered using a 10 keV Ar_15000_^+^ GCIB over an area of 600 × 600 µm^2^. Final sputter crater depths were measured using the Zygo NexView optical interferometer. Absorption spectra were obtained by using an Agilent Cary 60 UV–Vis spectrophotometer. Samples for DOSY NMR were prepared at 2 mg mL^−1^ in CDCl_3_. For the blend samples, the weight ratio of acceptor:CI was kept at 1:1. The DOSY experiments were measured at a temperature of 298 K on a Bruker 500 MHz AVANCE III HD spectrometer running TopSpin3.6.5 and equipped with a z-gradient Prodigy nitrogen-cooled 5 mm tuneable cryoprobe and a GRASP II gradient spectroscopy accessory providing a maximum gradient output (100%) of 53.5 G cm^−1^ (5.35 G cmA^−1^). The 1H DOSY spectra were collected using the Bruker pulse program ledbpgp2s at a frequency of 500.13 MHz with a spectral width of 5000 Hz (centered on 4.0 ppm) and 32768 data points, giving an acquisition time of 3.28 s. For sample MR PC_71_BM, a relaxation delay of 12.3 s was employed along with a diffusion time (large delta) of 20 ms and a longitudinal eddy current delay (LED) of 5 ms. Bipolar gradient pulses (little delta/2) of 1.35 ms and homospoil gradient pulses of 0.6 ms were used. The gradient strengths of the 2 homospoil pulses were −17.13% and −13.17%. 16 experiments of 24 transients each were collected with the bipolar gradient strength, initially at 2% (1st experiment), linearly increased to 95% (16th experiment). For MR [*P*]-aza[6]H:PC_71_BM sample, the DOSY experimental parameters were the same except a relaxation delay of 22 s was used and 160 transients collected. All gradient pulses were smoothed-square shaped (SMSQ10.100) and after each application a recovery delay of 200 us used. The data was processed using 32768 data points in the direct dimension, applying an exponential function with a line broadening of 1 Hz, and 64 data points in the indirect dimension. Further processing was achieved using the Bruker Dynamics Center software (version 2.8.b.4)—error estimation by Monte Carlo simulation with a confidence level of 95%. PPPc measurements were conducted with a visible 405 nm laser (ThorLabs CPS405) and an IR 1550 nm (LDM1550 Thorlabs) laser focused together through a dichroic mirror and a lens with a focus distance of 20 cm. To ensure the beams were colinearly focused, they were aligned to pass through a pinhole (ThorLabs P200D). A quarter wave plate and a linear polarizer were placed in front of the pump beam to generate CP light. The beams were modulated by an optical chopper (Thorlabs, MC1F60) with a modulation frequency of 717 Hz. A Zurich Instruments lock-in amplifier (MFLI 500 kHz/5MHz 60MSa/s) was used to apply bias and collect the photocurrent. The fluence of the beams was recorded with a Thorlabs power meter (PM400 Optical Power Meter) while the various fluences were obtained by a motorized filter wheel (FW212C Thorlabs). The pump beam size was measured using a Thorlabs camera beam profiler (BC106N-VIS/M). CD spectroscopy was performed using a CD spectropolarimeter. The *g*_abs_, indicating the ratio of circular dichroism to conventional absorption, was calculated by,

1 $${g}_{{{{\rm{abs}}}}}=2\frac{{A}_{{{{\rm{L}}}}}-{A}_{{{{\rm{R}}}}}}{{A}_{{{{\rm{L}}}}}+{A}_{{{{\rm{R}}}}}}=\frac{{{{\rm{CD}}}}}{32980\times A}$$

where *A*_L_ is the absorption for LCP light, *A*_R_ is the absorption for RCP light, CD is the value extracted from CD spectroscopy, and *A* is the absorbance of the sample. The photocurrent dissymmetry factor for CP-OPDs was defined by,

2 $${g}_{{{{\rm{Ph}}}}}=\frac{2\left({I}_{{{{\rm{LCP}}}}}-{I}_{{{{\rm{RCP}}}}}\right)}{\left({I}_{{{{\rm{LCP}}}}}+{I}_{{{{\rm{RCP}}}}}\right)}$$

where *I*_LCP_ and *I*_RCP_ are the photocurrents under LCP and RCP light illumination, respectively.

SR was calculated from the following equation,

3 $${{{\rm{SR}}}}\left(\lambda \right)=\frac{{J}_{{{{\rm{ph}}}}}}{{P}_{{{{\rm{in}}}}}}={{{\rm{EQE}}}}\frac{\lambda q}{{hc}}$$

where *J*_ph_ is the photogenerated current, *P*_in_ is the power of the incident light, *λ* is the wavelength of the incident light, EQE is the external quantum efficiency, *q* is the elementary charge, *h* is the Planck’s constant, and *c* is the speed of light, respectively. LDR was calculated by the equation,

4 $${{{\rm{LDR}}}}=20\log \frac{{I}_{\max }}{{I}_{\min }}$$

where *I*_max_ and *I*_min_ are the photocurrents at the upper and lower limits of the linear range, respectively. D^*^ was calculated as follows,

5 $${D}^{*}(\lambda,f)=\frac{\sqrt{A\triangle f}\cdot {{{\rm{SR}}}}}{{i}_{{{{\rm{n}}}}}(f)}$$

where *A* is the active area of the device, Δ*f* is the bandwidth of the measurement system, SR is the spectral responsivity, and *i*_n_ is the measured noise current, respectively.

## Supplementary information

Supplementary Information

Transparent Peer Review file

## Source data

Source Data Figure 1

Source Data Figure 2

Source Data Figure 3

## Data Availability

Additional data associated with this study can be found in the Supplementary Materials or are available from the corresponding authors upon request. Source data are provided with this paper.

## References

[1] Bronstein, H., Nielsen, C. B., Schroeder, B. C. & McCulloch, I. The role of chemical design in the performance of organic semiconductors. *Nat. Rev. Chem.* **4**, 66–77 (2020). 37128048 10.1038/s41570-019-0152-9

[2] Dimitriev, O. P. Dynamics of excitons in conjugated molecules and organic semiconductor systems. *Chem. Rev.* **122**, 8487–8593 (2022). 35298145 10.1021/acs.chemrev.1c00648

[3] Greenfield, J. L. et al. Pathways to increase the dissymmetry in the interaction of chiral light and chiral molecules. *Chem. Sci.* **12**, 8589–8602 (2021). 34257860 10.1039/d1sc02335g PMC8246297

[4] Sato, S. et al. Chiral intertwined spirals and magnetic transition dipole moments dictated by cylinder helicity. *Proc. Natl. Acad. Sci.* **114**, 13097–13101 (2017). 29180419 10.1073/pnas.1717524114 PMC5740620

[5] Uceda, R. G. et al. Can magnetic dipole transition moment be engineered?. *Angew. Chem. Int. Ed.* **63**, e202316696 (2024). 10.1002/anie.202316696 38051776

[6] Zhang, Y. et al. Chiral polyacetylene with thermally activated delayed fluorescence feature for high-performance circularly polarized light detection. *Chem. Mater.* **36**, 3369–3380 (2024).

[7] Liu, L. et al. Chiral non-fullerene acceptor enriched bulk heterojunctions enable high-performance near-infrared circularly polarized light detection. *Small* **18**, 2202941 (2022). 10.1002/smll.202202941 35808959

[8] Shang, X. et al. Surface-doped quasi-2D chiral organic single crystals for chiroptical sensing. *ACS Nano* **14**, 14146–14156 (2020). 33120505 10.1021/acsnano.0c07012

[9] Song, I. et al. Helical polymers for dissymmetric circularly polarized light imaging. *Nature* **617**, 92–99 (2023). 37138111 10.1038/s41586-023-05877-0

[10] Wan, L. et al. Sensitive near-infrared circularly polarized light detection via non-fullerene acceptor blends. *Nat. Photonics* **17**, 649–655 (2023).

[11] Zhu, D. et al. Organic donor-acceptor heterojunctions for high-performance circularly polarized light detection. *Nat. Commun.* **13**, 3454 (2022). 35705562 10.1038/s41467-022-31186-7 PMC9200767

[12] Zhang, L. et al. Extended perylene diimide double-heterohelicenes as ambipolar organic semiconductors for broadband circularly polarized light detection. *Nat. Commun.* **12**, 142 (2021). 33420007 10.1038/s41467-020-20390-y PMC7794514

[13] Gilot, J. et al. Polymer photovoltaic cells sensitive to the circular Polarization of light. *Adv. Mater.* **22**, E131–E134 (2010). 20641093 10.1002/adma.200903995

[14] Yang, Y., da Costa, R. C., Fuchter, M. J. & Campbell, A. J. Circularly polarized light detection by a chiral organic semiconductor transistor. *Nat. Photonics* **7**, 634–638 (2013).

[15] Liu, L. et al. Building supramolecular chirality in bulk heterojunctions enables amplified dissymmetry current for high-performing circularly polarized light detection. *ACS Mater. Lett.* **4**, 401–409 (2022).

[16] He, W.-M. et al. Atomically precise chiral metal nanoclusters for circularly polarized light detection. *Angew. Chem. Int. Ed.* **63**, e202407887 (2024). 10.1002/anie.202407887 38802322

[17] Li, C. et al. Twisting of porphyrin by assembly in a metal-organic framework, yielding chiral photoconducting films for circularly-polarized-light detection. *Angew. Chem. Int. Ed.* **62**, e202217377 (2023). 10.1002/anie.202217377 36515401

[18] Kwon, Y., Jung, J.-Y., Lee, W. B. & Oh, J. H. Axially chiral organic semiconductors for visible-blind uv-selective circularly polarized light detection. *Adv. Sci.* **11**, 2308262 (2024). 10.1002/advs.202308262 PMC11005684 38311579

[19] Ward, M. D. et al. Highly selective high-speed circularly polarized photodiodes based on π-conjugated polymers. *Adv. Opt. Mater.* **10**, 2101044 (2022).

[20] Hu, R., Lu, X., Hao, X. & Qin, W. An organic chiroptical detector favoring circularly polarized light detection from near-infrared to ultraviolet and magnetic-field-amplifying dissymmetry in detectivity. *Adv. Mater.* **35**, 2211935 (2023). 10.1002/adma.202211935 36916071

[21] Gu, Q. et al. Constructing chiral covalent-organic frameworks for circularly polarized light detection. *Adv. Mater.* **36**, 2306414 (2024). 10.1002/adma.202306414 37589261

[22] Liu, L., Wei, Z. & Meskers, S. C. J. Semi-transparent, chiral organic photodiodes with incident direction-dependent selectivity for circularly polarized light. *Adv. Mater.* **35**, 2209730 (2023). 10.1002/adma.202209730 36577393

[23] Shi, W. et al. Fullerene desymmetrization as a means to achieve single-enantiomer electron acceptors with maximized chiroptical responsiveness. *Adv. Mater.* **33**, 2004115 (2021). 33225503 10.1002/adma.202004115 PMC11468824

[24] Shang, X. et al. Supramolecular nanostructures of chiral perylene diimides with amplified chirality for high-performance chiroptical sensing. *Adv. Mater.* **29**, 1605828 (2017). 10.1002/adma.201605828 28370408

[25] Schulz, M. et al. Chiral excitonic organic photodiodes for direct detection of circular polarized light. *Adv. Funct. Mater.* **29**, 1900684 (2019).

[26] Kim, N. Y. et al. Chiroptical-conjugated polymer/chiral small molecule hybrid thin films for circularly polarized light-detecting heterojunction devices. *Adv. Funct. Mater.* **29**, 1808668 (2019).

[27] Gao, K. et al. Reversal of chirality in solutions and aggregates of chiral tetrachlorinated diperylene diimides towards efficient circularly polarized light detection. *Mater. Horiz.* **12**, 1903–1912 (2025). 39688194 10.1039/d4mh01435a

[28] Song, I. et al. Helical donor–acceptor bulk heterojunctions for dissymmetric circularly polarized light detection. *Chem. Eng. J.* **505**, 158991 (2025).

[29] Ward, M. D. et al. Best practices in the measurement of circularly polarised photodetectors. *J. Mater. Chem. C.* **10**, 10452–10463 (2022). 10.1039/d2tc01224c PMC9332130 35967516

[30] Stachelek, P., MacKenzie, L., Parker, D. & Pal, R. Circularly polarised luminescence laser scanning confocal microscopy to study live cell chiral molecular interactions. *Nat. Commun.* **13**, 553 (2022). 35087047 10.1038/s41467-022-28220-z PMC8795401

[31] Kwon, J., Park, K. H., Choi, W. J., Kotov, N. A. & Yeom, J. Chiral spectroscopy of nanostructures. *Acc. Chem. Res.* **56**, 1359–1372 (2023). 37256726 10.1021/acs.accounts.2c00756

[32] Almanakly, A. et al. Deterministic remote entanglement using a chiral quantum interconnect. *Nat. Phys.* **21**, 825–830 (2025).

[33] Lodahl, P. et al. Chiral quantum optics. *Nature* **541**, 473–480 (2017). 28128249 10.1038/nature21037

[34] Ciammaruchi, L., Zapata-Arteaga, O., Gutiérrez-Fernández, E., Martin, J. & Campoy-Quiles, M. Structure-dependent photostability of ITIC and ITIC-4F. *Mater. Adv.* **1**, 2846–2861 (2020).

[35] Dalisay, D. S., Quach, T., Nicholas, G. N. & Molinski, T. F. Amplification of the cotton effect of a single chromophore through liposomal ordering—stereochemical assignment of plakinic acids I and J. *Angew. Chem. Int. Ed.* **48**, 4367–4371 (2009). 10.1002/anie.200900888 PMC2872062 19437515

[36] Kumar, A. et al. Morphological control of Y6 thin films reveals charge transfer is facilitated by co-facial interactions. *J. Phys. Chem. Lett.* **16**, 1367–1375 (2025). 39878327 10.1021/acs.jpclett.4c03119

[37] Pinto, R. M., Maçoas, E. M. S. & Alves, H. Enhanced conductivity and photoresponse at a rubrene single-crystal–PCBM film interface. *J. Mater. Chem. C.* **2**, 3639–3644 (2014).

[38] Colbourne, A. A., Morris, G. A. & Nilsson, M. Local covariance order diffusion-ordered spectroscopy: a powerful tool for mixture analysis. *J. Am. Chem. Soc.* **133**, 7640–7643 (2011). 21528860 10.1021/ja2004895

[39] Bakulin, A. A. et al. The role of driving energy and delocalized states for charge separation in organic semiconductors. *Science* **335**, 1340–1344 (2012). 22362882 10.1126/science.1217745

[40] Price, M. B. et al. Free charge photogeneration in a single-component high photovoltaic efficiency organic semiconductor. *Nat. Commun.* **13**, 2827 (2022). 35595764 10.1038/s41467-022-30127-8 PMC9122989

[41] Pan, J. et al. Operando dynamics of trapped carriers in perovskite solar cells observed via infrared optical activation spectroscopy. *Nat. Commun.* **14**, 8000 (2023). 38044384 10.1038/s41467-023-43852-5 PMC10694143

[42] Liu, X. et al. Efficient organic solar cells with extremely high open-circuit voltages and low voltage losses by suppressing nonradiative recombination losses. *Adv. Energy Mater.* **8**, 1801699 (2018).

[43] Sharma, A. et al. Semitransparent organic photovoltaics utilizing intrinsic charge generation in non-fullerene acceptors. *Adv. Mater.* **36**, 2305367 (2024). 10.1002/adma.202305367 38100279

[44] Wu, Y.-L., Fukuda, K., Yokota, T. & Someya, T. A highly responsive organic image sensor based on a two-terminal organic photodetector with photomultiplication. *Adv. Mater.* **31**, 1903687 (2019). 10.1002/adma.201903687 31495992

[45] Takenaka, N., Sarangthem, R. S. & Captain, B. Helical chiral pyridine N-oxides: a new family of asymmetric catalysts. *Angew. Chem. Int. Ed.* **47**, 9708–9710 (2008). 10.1002/anie.200803338 18988214

