# Supplementary material for: Trap-assisted circularly polarized organic photodetector
Source: Nat Commun. 2026 Apr 25;17:5710. doi: 10.1038/s41467-026-72474-w (PMC13319231; doi:10.1038/s41467-026-72474-w)
Supplement: Supplementary file 1 — Supplementary Information [file 41467_2026_72474_MOESM1_ESM.pdf]

Supplementary Information

Trap-assisted circularly polarized organic photodetector

Hongki Kim[1*], Zhuoran Qiao[1], Marie Houot[1], Martina Rimmele[1], Francesco Furlan[1], Matilde Brunetta[2,3], David Reger[1,4], Filip Aniés[5], Rahil Haria[2], Edoardo Angela[1], Ding Ding[1], Michele Conroy[2], Jessica Wade[2,6], Martin Heeney[1,5,6], Artem A. Bakulin[1,6], Nicola Gasparini[1,6*] and Matthew J. Fuchter[1,4,6*]

[1]Department of Chemistry, Molecular Science Research Hub, Imperial College London, London W12 0BZ, UK.

[2]Department of Materials, Imperial College London, Prince Consort Road, London SW7 2AZ, UK

[3]Department of Chemistry, University College London, 20 Gordon Street, London WC1H 0AJ, UK

[4]Department of Chemistry, Chemistry Research Laboratory, University of Oxford, 12 Mansfield Road, Oxford, OX1 3TA, UK.

[5]King Abdullah University of Science and Technology (KAUST), Thuwal, 23955-6900 Saudi Arabia

[6]Centre for Processable Electronics, Imperial College London, London SW7 2AZ, UK.

*Corresponding authors: hongki.kim@imperial.ac.uk; n.gasparini@imperial.ac.uk; matthew.fuchter@chem.ox.ac.uk

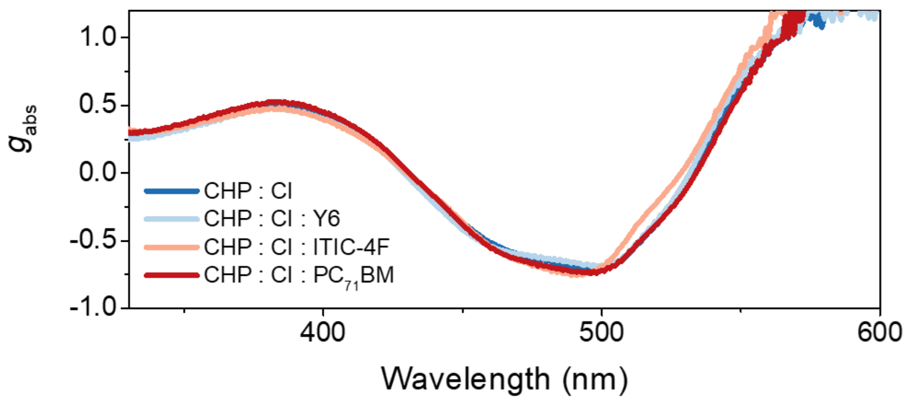

Supplementary Figure 1| g_{abs} spectra of CHP:CI blend films with a different enantiomer. CHP:CI (10 wt% loading of [*M*]-aza[6]H into F8T2 polymer) film without acceptor or with different types of acceptors. The CHP:CI thin films with [*M*]-aza[6]H were prepared with the same processing conditions and concentrations used for fabricating the CHP:CI film with [*P*]-aza[6]H.

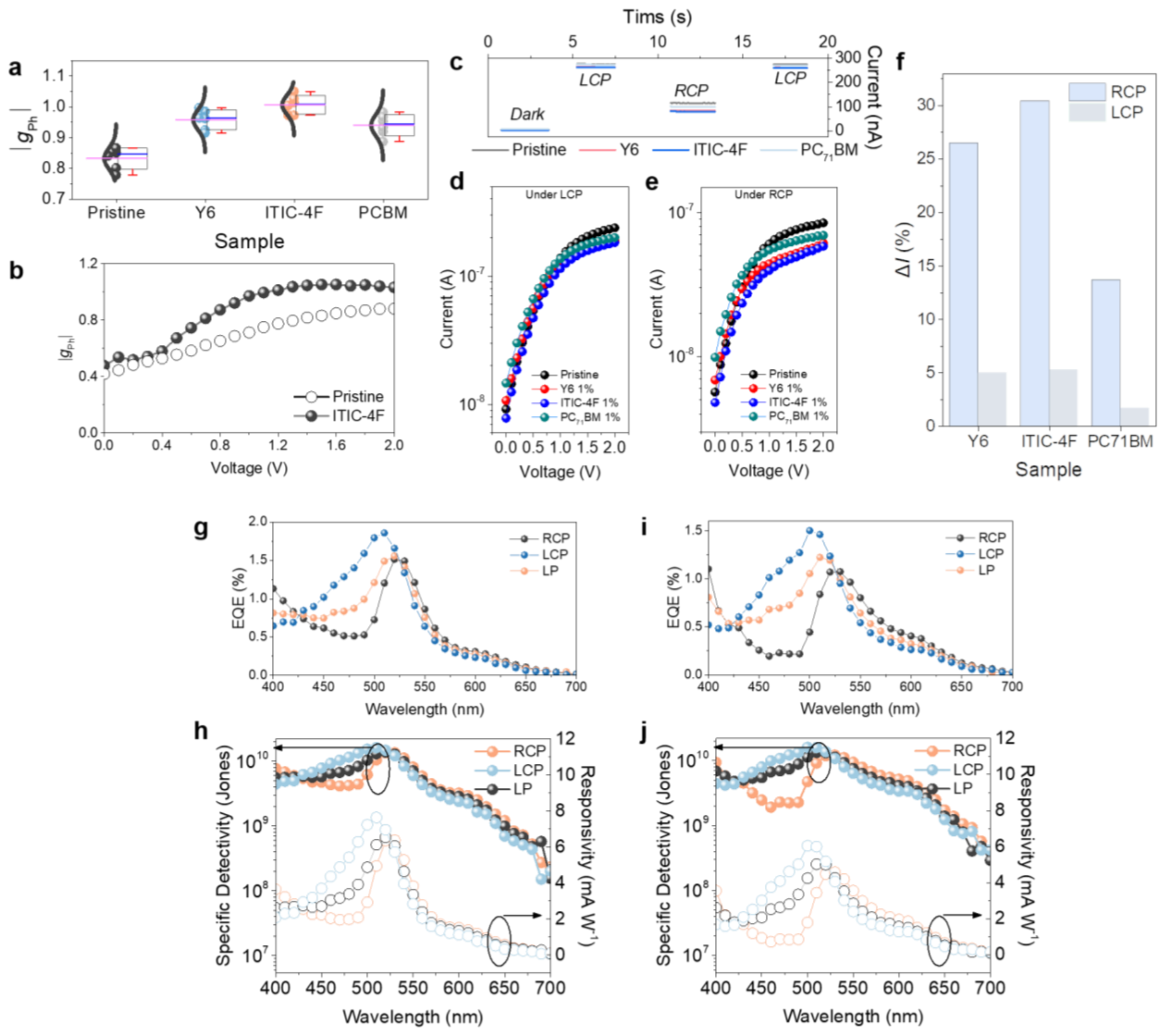

Supplementary Figure 2| g_{Ph} trends and device characteristics of CP-OPDs by using CHP:CI ([*M*]-aza[6]H) matrices, without acceptor or with acceptors. a, Statistics of g_{Ph} for each type of CP-OPDs under 470 nm CP light (23.6 mW cm^{-2}). Box range is defined by standard deviation of g_{Ph} for 6 devices. Red, blue, and pink lines indicate whisker, median line, and mean line, respectively (pristine: median (0.84796), upper (0.85432) and lower quartiles (0.81087), upper (0.86576) and lower whiskers (0.77820), interquartile range (0.04345), no outliers; Y6: median (0.96346), upper (0.98096) and lower quartiles (0.92401), upper (0.99625) and lower whiskers (0.91478), interquartile range (0.05695), no outliers; ITIC-4F: median (1.00490), upper (1.02325) and lower quartiles (0.97211), upper (1.04960) and lower whiskers (0.97180), interquartile range (0.05114), no outliers; $PC_{71}BM$: median (0.94602), upper (0.96992) and lower quartiles (0.92151), upper (0.98237) and lower whiskers (0.88615), interquartile range

(0.04841), no outliers). **b**, Voltage-dependent g_{Ph} for the pristine and the ITIC-4F-incorporated CP-OPD. **c,** Time-dependent CP light detection characteristics for each type of device. **d,e,** *I*−*V* curves of each CP-OPDs under LCP (**d**) and RCP illumination (**e**). **f,** Photocurrent difference under RCP and LCP compared to those from the pristine device. **g–j,** EQE, Spectral *R*, and D^* of pristine CP-OPDs (**g,h**) or ITIC-4F-introduced CP-OPDs (**i,j**) under the linear polarized (LP), RCP, and LCP excitation.

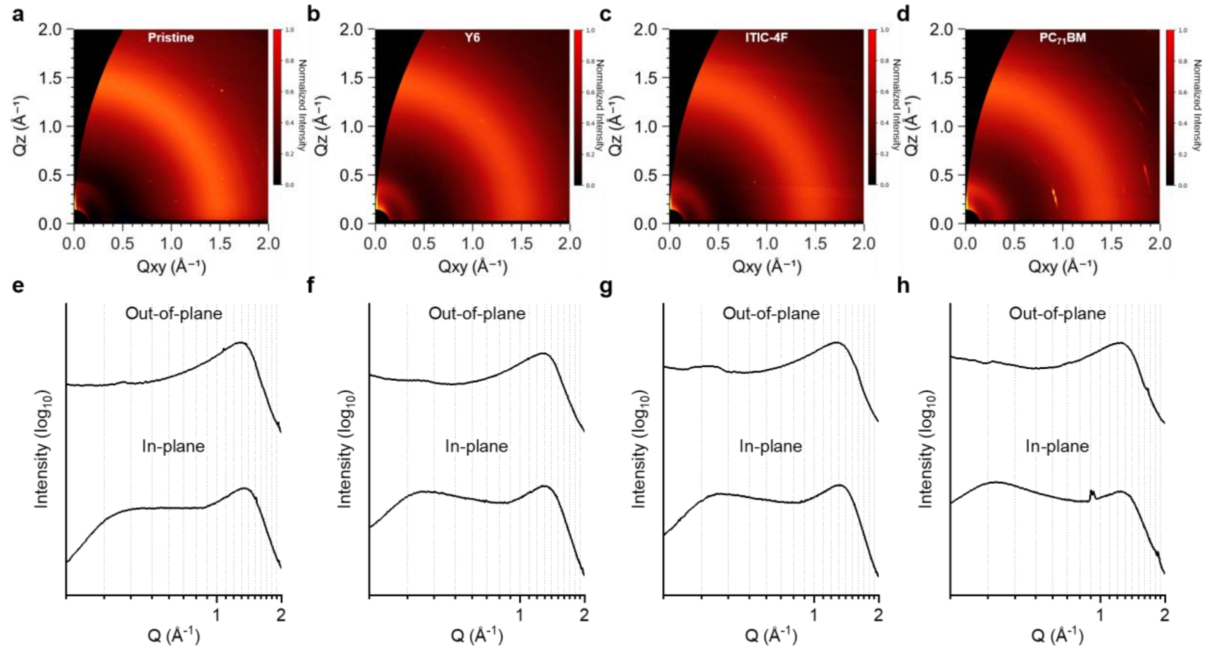

Supplementary Figure 3| GIWAXS of chiral matrices. a−d, 2D GIWAXS images of pure CHP:CI thin film (**a**), Y6-introduced (**b**), ITIC-4F-introduced (**c**), and $PC_{71}BM$-introduced CHP:CI thin films (**d**). **e−h**, 1D line plots of CHP:CI thin film (**e**), Y6-introduced (**f**), ITIC-4F-introduced (**g**), and $PC_{71}BM$-introduced CHP:CI thin films (**h**). The CHP:CI thin films were prepared with the same processing conditions used for fabricating the CP-OPDs.

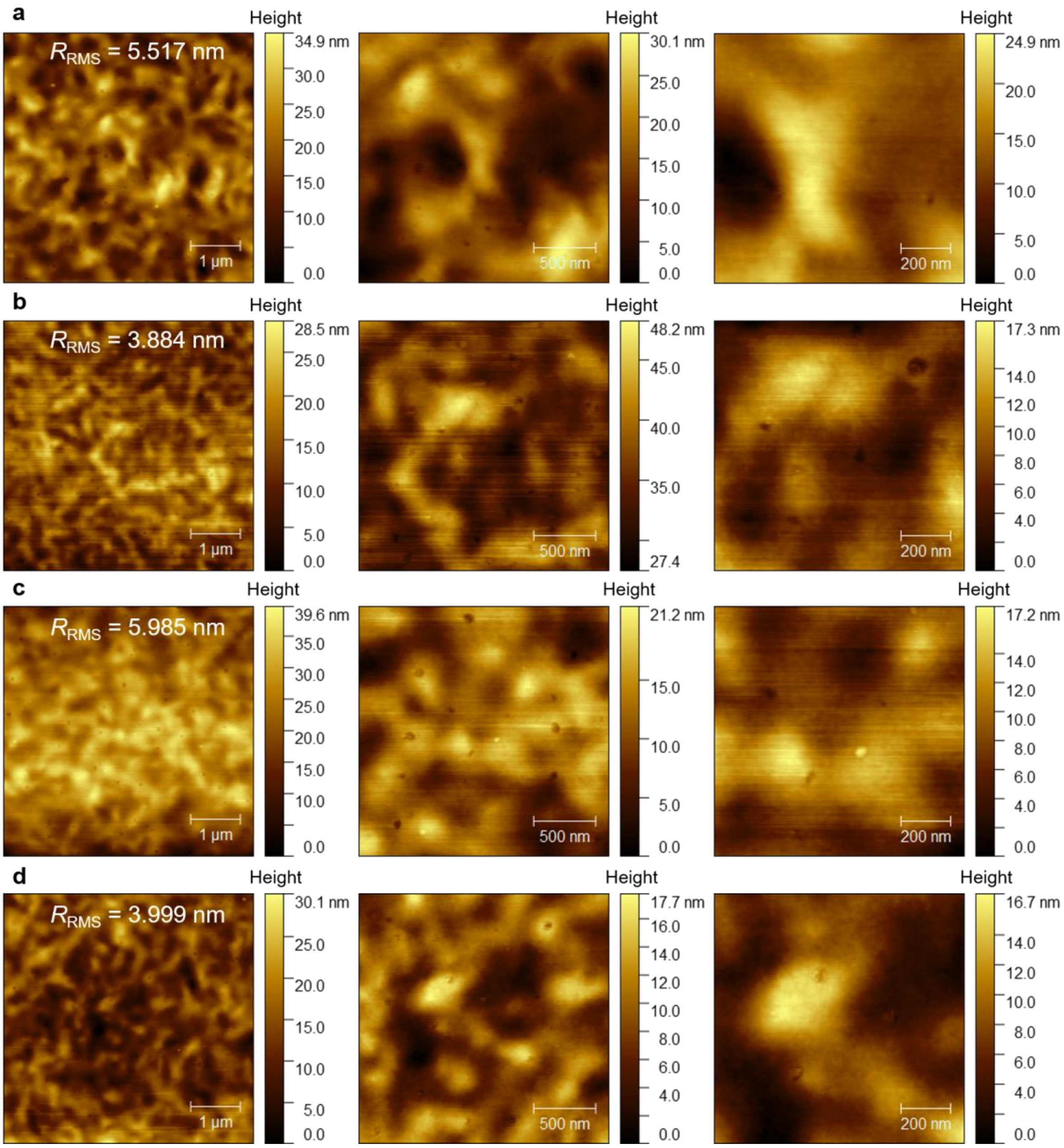

Supplementary Figure 4| AFM images of chiral matrices. a, Reference CHP:CI thin film. **b**, Y6-introduced CHP:CI thin films. **c**, ITIC-4F-introduced CHP:CI thin films. **d**, $PC_{71}BM$-introduced CHP:CI thin films. Root mean square roughness (R_{RMS}) was shown for each AFM image. The CHP:CI thin films were prepared with the same processing conditions used for fabricating the CP-OPDs.

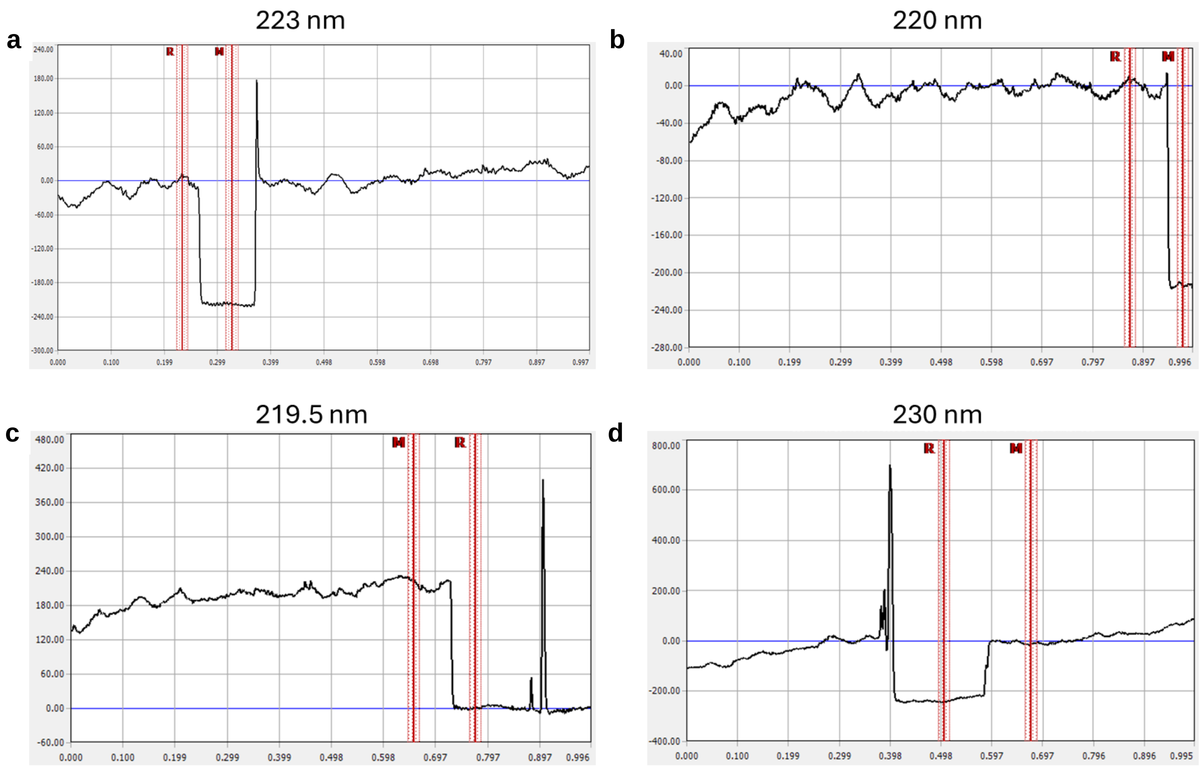

Supplementary Figure 5| Thickness profiles of chiral matrices. **a**, Reference CHP:CI thin film. **b**, Y6-introduced CHP:CI thin films. **c**, ITIC-4F-introduced CHP:CI thin films. **d**, $PC_{71}BM$-introduced CHP:CI thin films. The CHP:CI thin films were prepared with the same processing conditions used for fabricating the CP-OPDs.

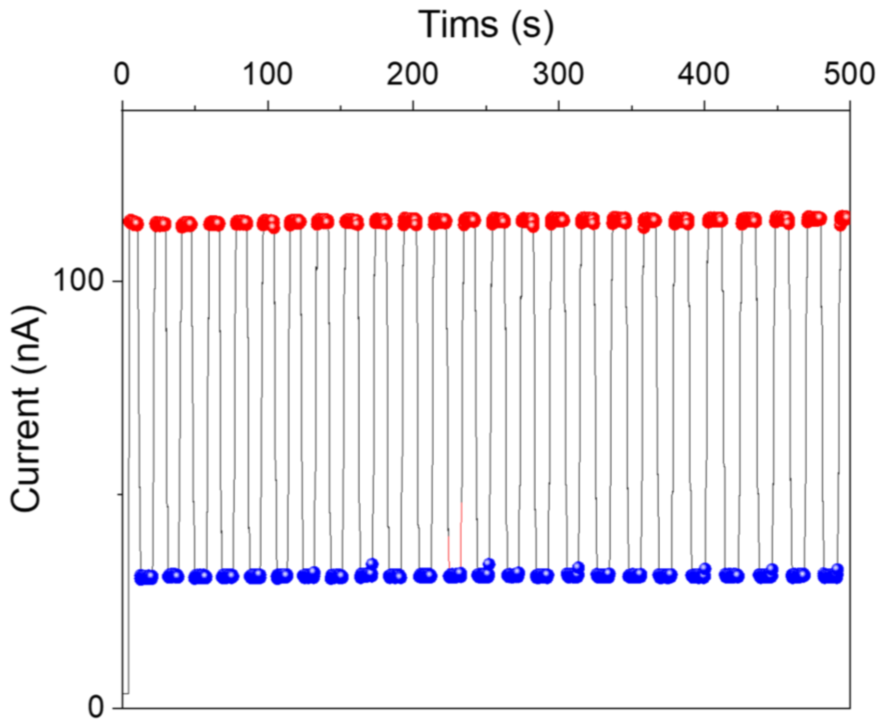

Supplementary Figure 6| Time-dependent CP light detection of a CP-OPD with ITIC-4F.
Red and blue dots represent RCP and LCP states, respectively.

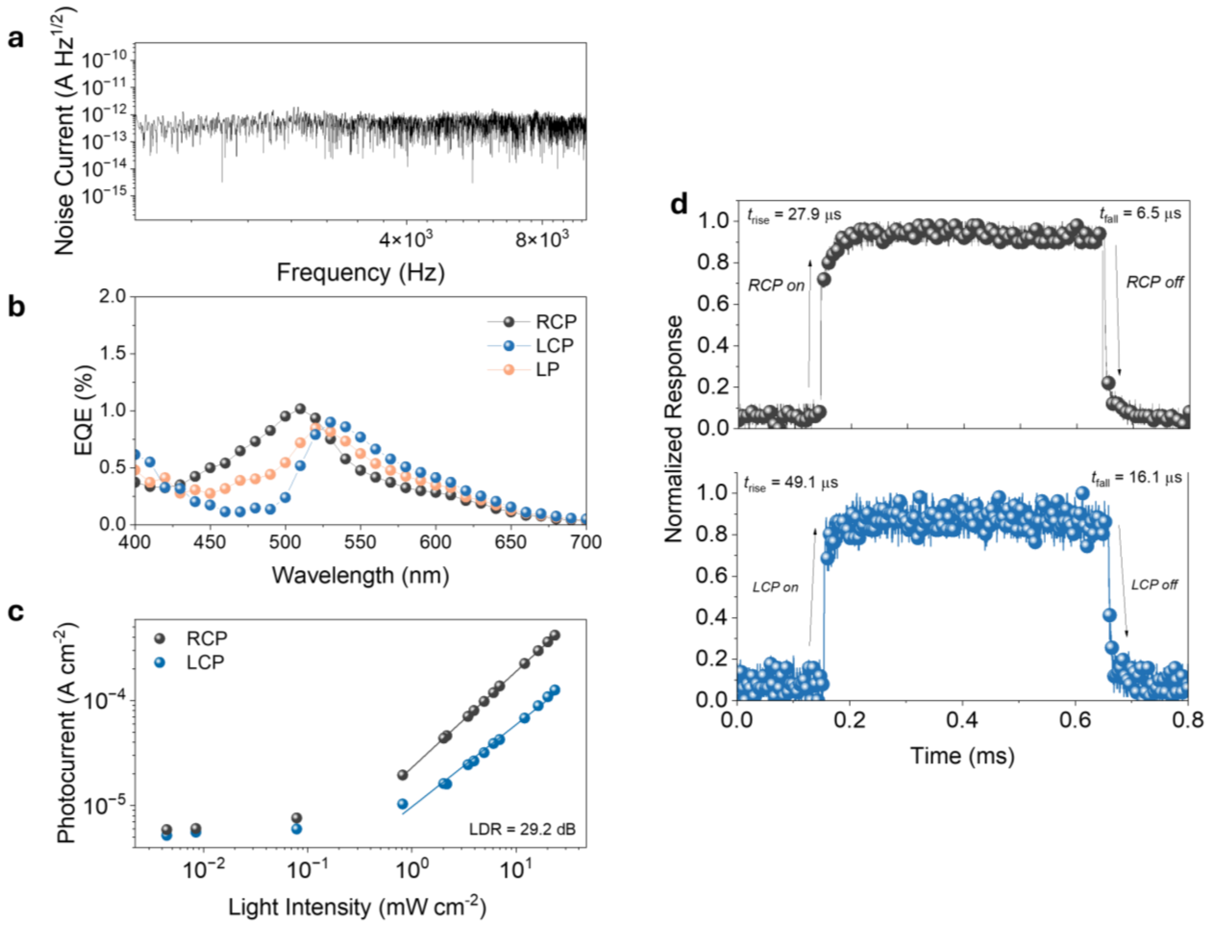

Supplementary Figure 7| Photo-response characteristics of ITIC-4F-introduced CP-OPDs. a, Noise current spectral density. **b**, EQE spectra of CP-OPD with the different polarization of light. **c**, LDR under CP light. **d**, Rise and fall time under RCP and LCP light excitation.

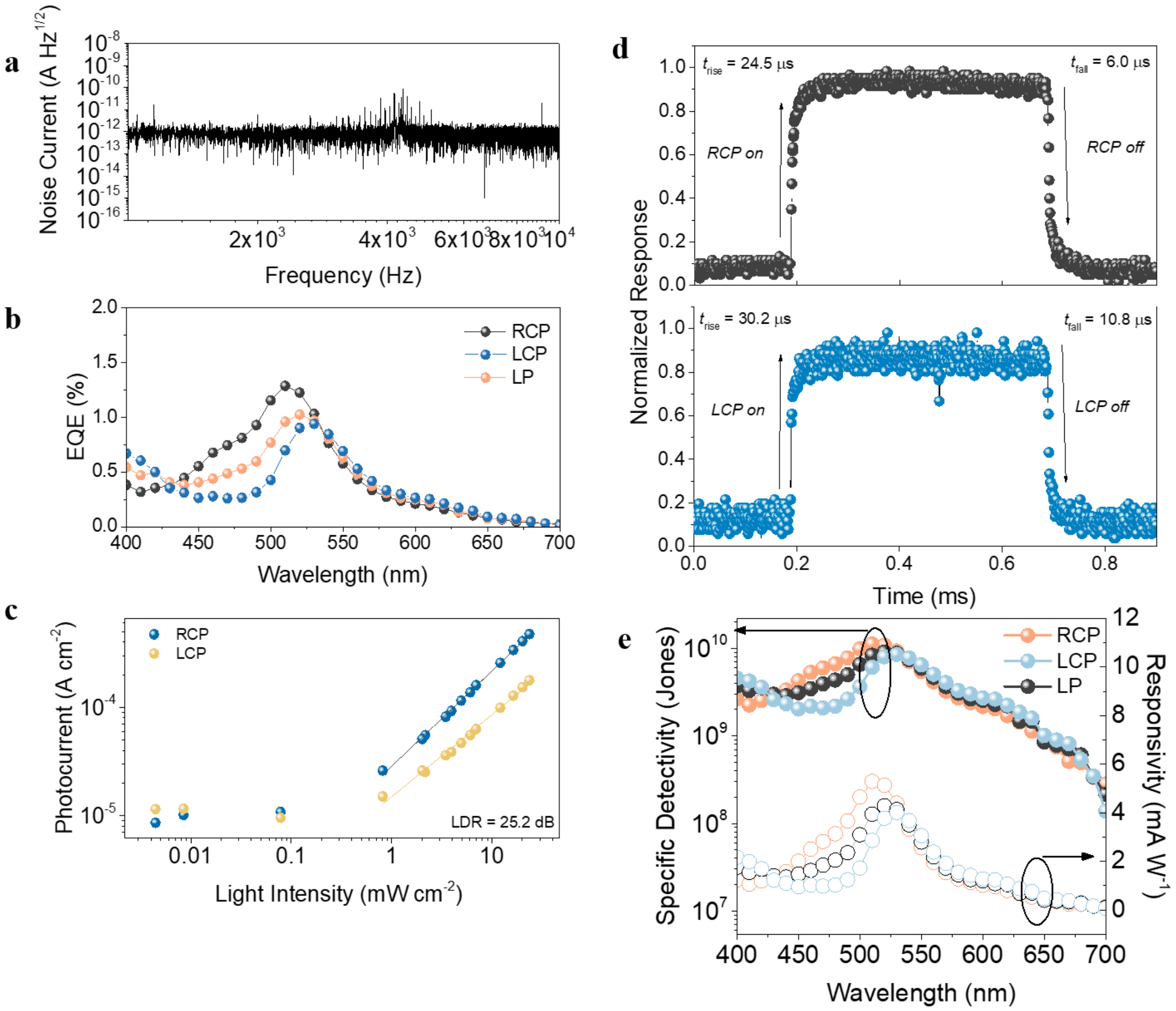

Supplementary Figure 8| Photo-response characteristics of pristine CP-OPDs. a, Noise current spectral density. **b**, EQE spectra of CP-OPD with the different polarization of light. **c**, LDR under CP light. **d**, Rise and fall time under RCP and LCP light excitation. e, Spectral *R* and *D** of ITIC-4F-introduced CP-OPDs under the linear polarized (LP), RCP, and LCP excitation.

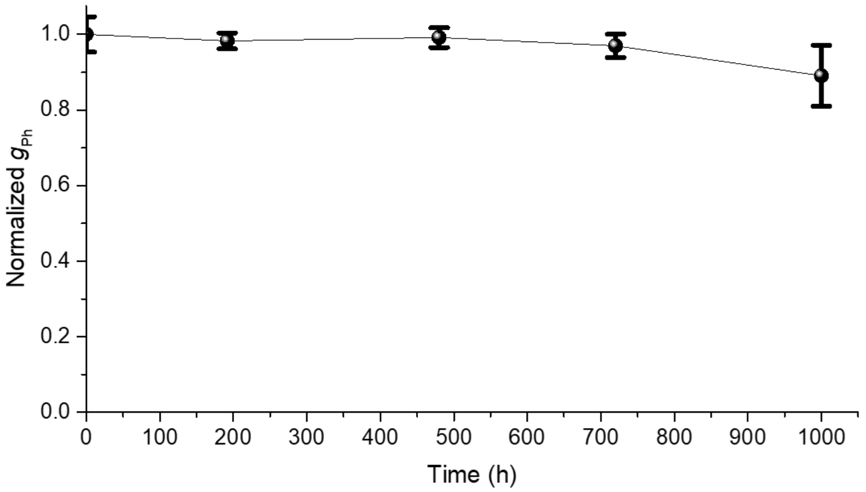

Supplementary Figure 9| Stability test represented by the normalized g_{Ph} of unencapsulated ITIC-4F-incorporated CP-OPDs. The devices were stored in a nitrogen-filled glove box, and measurements were performed in air. Error bar is defined by SD of g_{Ph} in 8 devices. Device measurement conditions were performed at the same wavelength (470 nm) and light intensity (23.6 mW cm^{-2}).

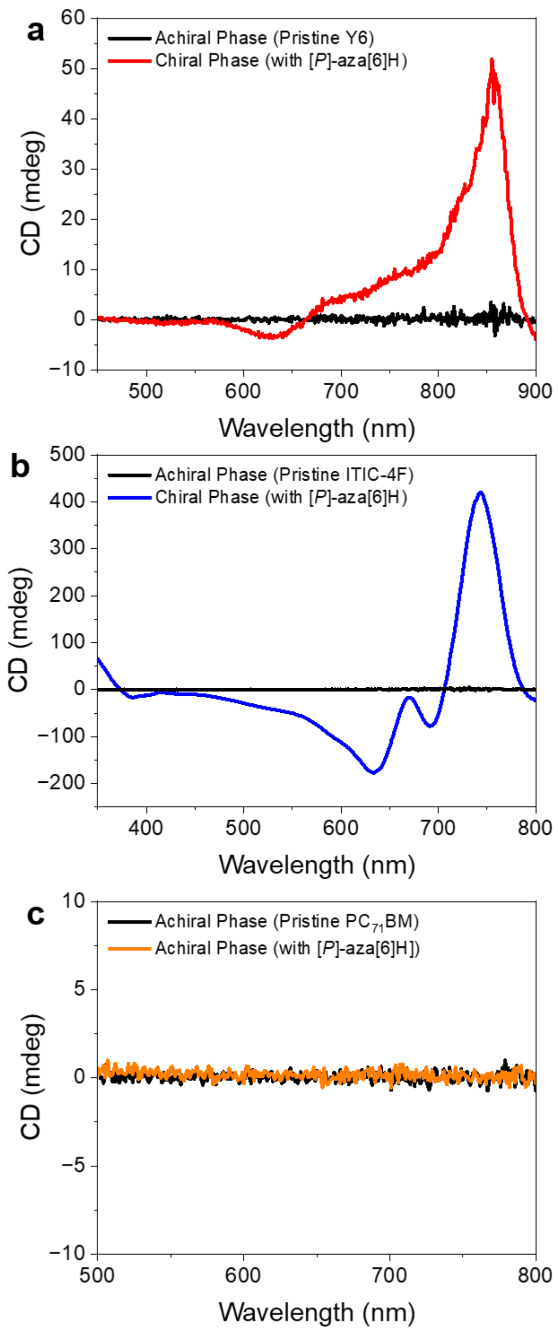

Supplementary Figure 10| Chiroptical properties of acceptor:CI films. a, CD spectrum of pristine Y6 thin film and its mixture with [*P*]-aza[6]H. **b**, CD spectrum of pristine ITIC-4F thin film and its mixture with [*P*]-aza[6]H. **c**, CD spectrum of pristine $PC_{71}BM$ thin film and its mixture with [*P*]-aza[6]H. Y6 and ITIC-4F exhibited clear chiroptical properties when mixed with CIs, whereas $PC_{71}BM$ did not show any chiroptical properties. Thin films were fabricated from the solution consisting of each acceptor and CI (10 mg ml^{-1}, 1:1 weight ratio of [*P*]-aza[6]H). Y6 had low solubility in toluene, especially when used in high concentrations. To address this issue and ensure film quality, a small amount (= 10 vol%) of chloroform is added to the toluene.

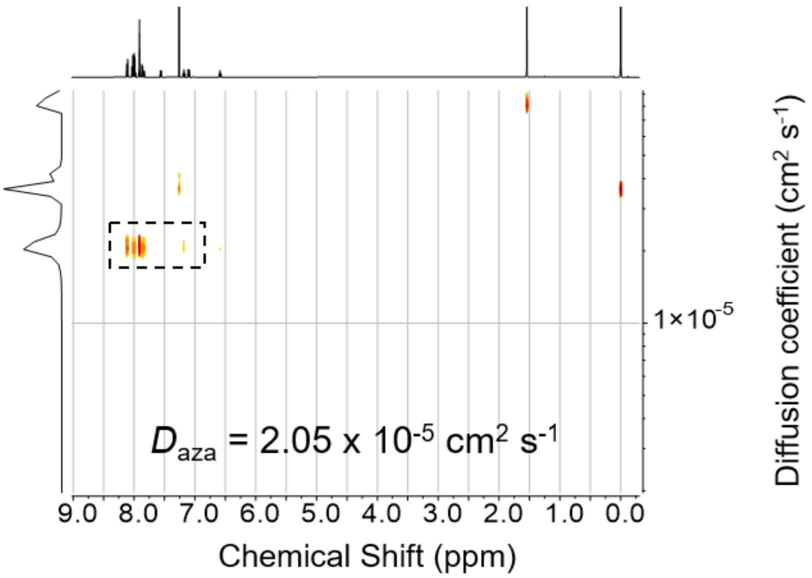

Supplementary Figure 11| DOSY study. DOSY spectrum of pure [*P*]-aza[6]H.

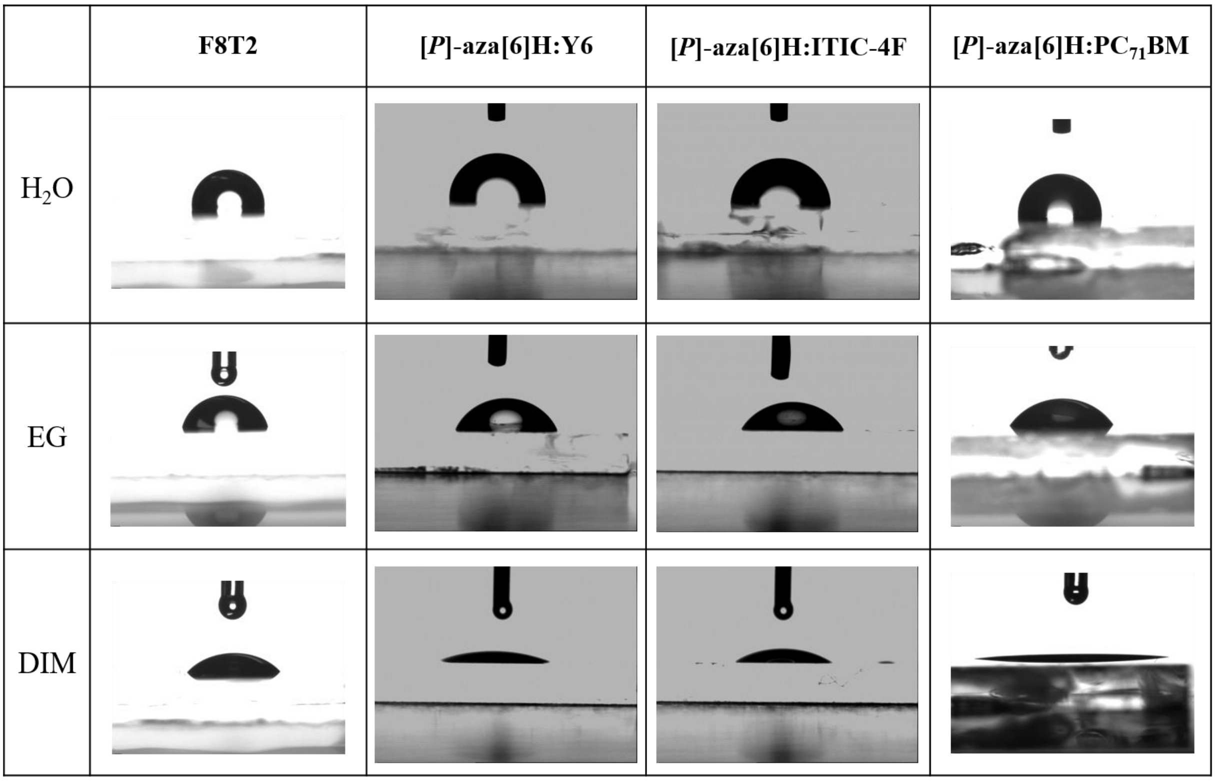

	θ_{water} (°)	θ_{EG} (°)	θ_{DIM} (°)	γ (mM/m)	χ
F8T2	100.8	75.2	48.8	31.5	
[*P*]-aza[6]H:Y6	96.3	66.4	27.5	40.7	0.58
[*P*]-aza[6]H:ITIC-4F	93.4	60.6	34.5	39.5	0.45
[*P*]-aza[6]H:$PC_{71}BM$	94.2	57.9	4.6	46.7	1.49

Supplementary Figure 12| Contact angle measurements. The surface energy was calculated by using Owens–Wendt–Rabel–Kaelble (OWRK) method[1]. The Flory-Huggins interaction parameter between F8T2 and [*P*]-aza[6]H:acceptor was calculated by using $\chi = (\sqrt{\gamma_{F8T2}} - \sqrt{\gamma_{aza[6]H:acceptor}})^2$, where γ_{F8T2} and $\gamma_{aza[6]H:accpetor}$ are surface energies of F8T2 and [*P*]-aza[6]H:acceptor blend. Surface energy extraction based on contact angle measurements was performed under conditions that closely reflect the actual thin-film environment in the devices. In our system, the active layer is predominantly composed of the host polymer F8T2, together with 10 wt% [*P*]-aza[6]H and a significantly smaller fraction (1 wt%) of the acceptor. Accordingly, contact angle measurements were carried out on neat F8T2 films and on [*P*]-aza[6]H:acceptor blend films, since F8T2 and [*P*]-aza[6]H constitute the major components of the thin film. In addition, [*P*]-aza[6]H:acceptor blend films blend films were prepared with an acceptor to [*P*]-aza[6]H ratio of 1:10, identical to that used in the devices.

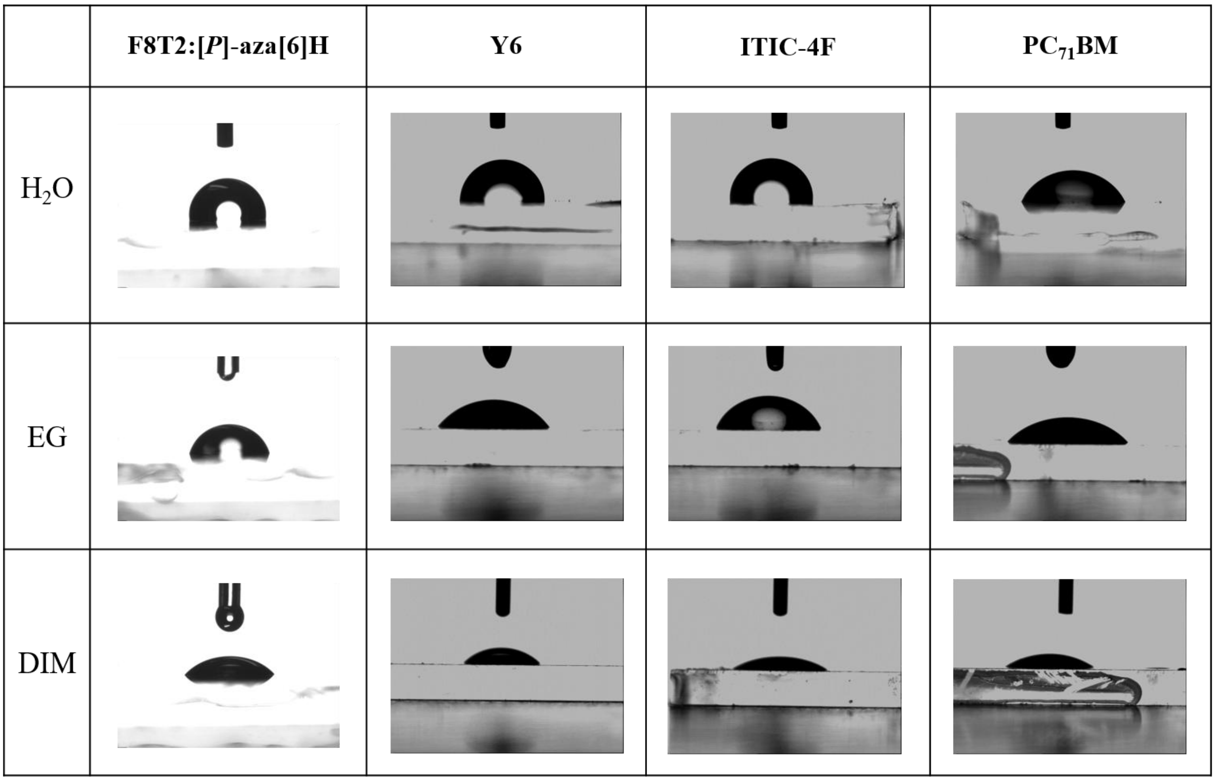

	θ_{water} (°)	θ_{EG} (°)	θ_{DIM} (°)	γ (mM/m)	χ
F8T2:[*P*]-aza[6]H	99.8	75.6	49.6	30.9	
Y6	92.9	53.3	46.0	37.1	0.28
ITIC-4F	99.1	68.2	45.3	35.7	0.17
$PC_{71}BM$	65.4	48.3	33.8	44.0	1.15

Supplementary Figure 13| Contact angle measurements. The surface energy was calculated by using Owens–Wendt–Rabel–Kaelble (OWRK) method[1]. The Flory-Huggins interaction parameter between F8T2 and [*P*]-aza[6]H:acceptor was calculated by using $\chi = (\sqrt{\gamma_{F8T2:[P]\text{-aza[6]H}}} - \sqrt{\gamma_{acceptor}})^2$, where $\gamma_{F8T2:[P]\text{-aza[6]H}}$ and $\gamma_{accpetor}$ are surface energies of F8T2:[*P*]-aza[6]H blend and acceptor. To ensure relevance to device conditions, films were processed using toluene, the same solvent employed during device fabrication.

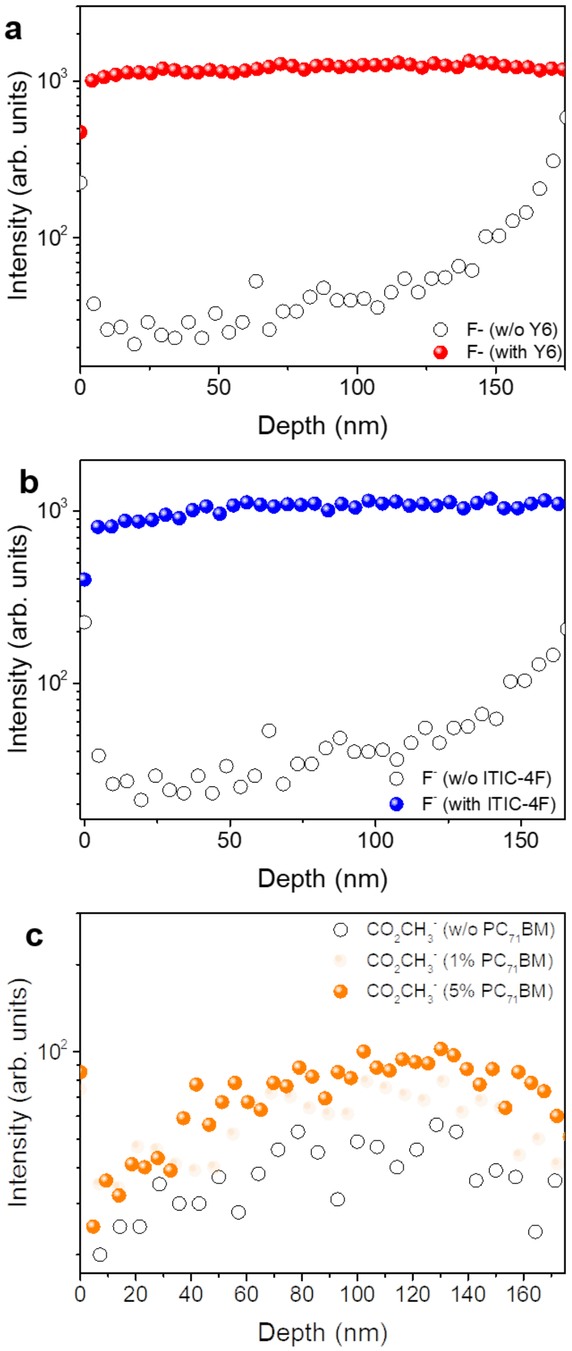

Supplementary Figure 14| ToF-SIMS results of chiral matrices. a, Y6-introduced chiral matrix. **b**, ITIC-4F-introduced chiral matrix. **c**, $PC_{71}BM$-introduced chiral matrix. The acceptor-introduced CHP:CI thin films were prepared with the same processing conditions used for fabricating the CP-OPDs.

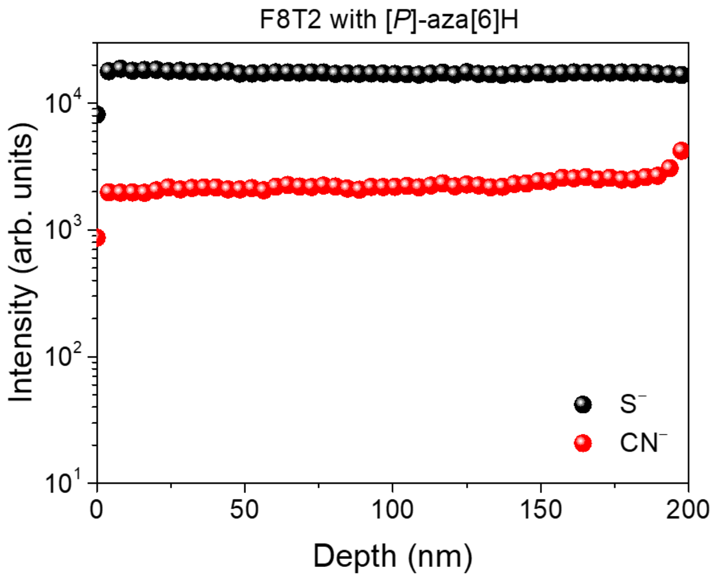

Supplementary Figure 15| ToF-SIMS result of pristine CHP:CI thin film. The CHP:CI thin film was prepared with the same processing conditions used for fabricating the CP-OPDs.

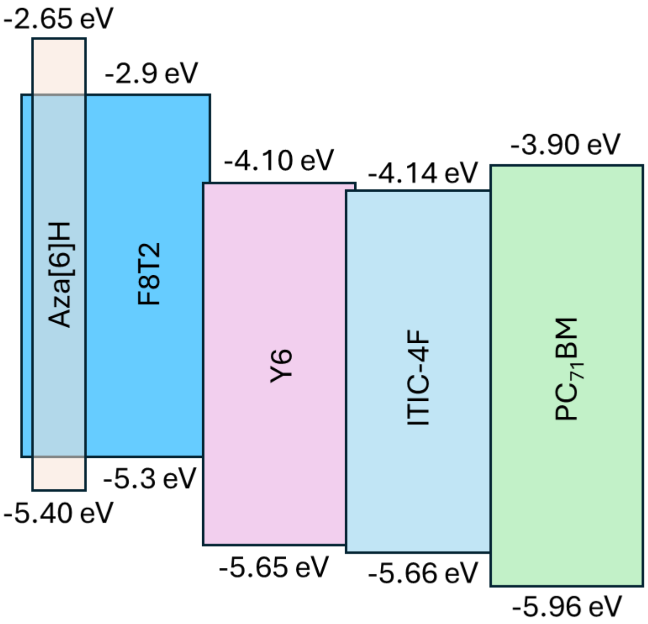

Supplementary Figure 16| Band alignment. Energy levels of F8T2[2], aza[6]H[3], Y6[4], ITIC-4F[5], and $PC_{71}BM$[6].

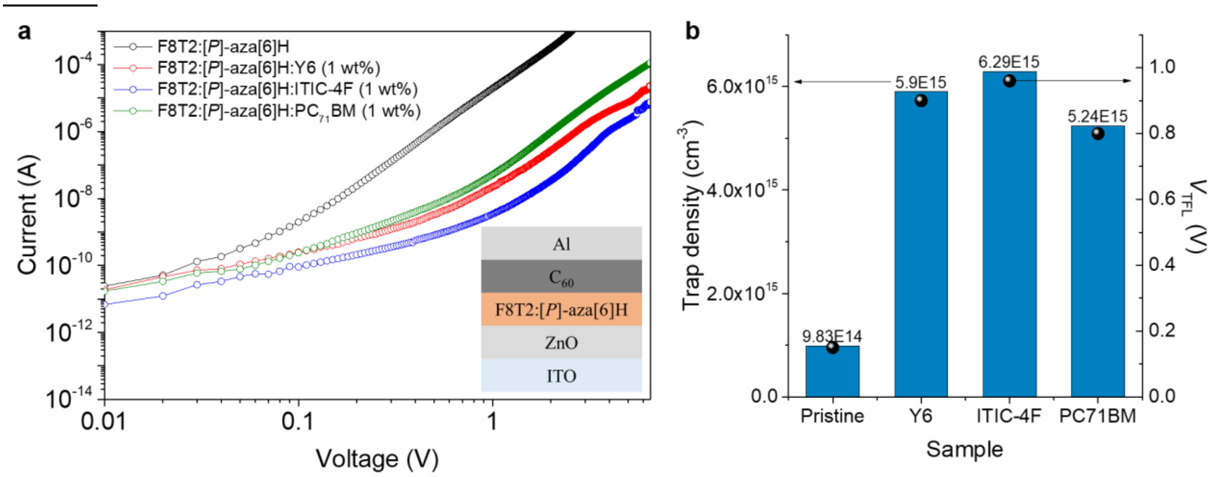

Supplementary Figure 17| SCLC measurements of electron-only devices. a, *I*–*V* curves of electron-only devices without or with acceptors and inset device configuration is used for electron-only device. **b,** Electron trap density (left-axis) was calculated by using trap-filled limit voltage (V_{TFL}) value (right-axis) based on SCLC technique[7].

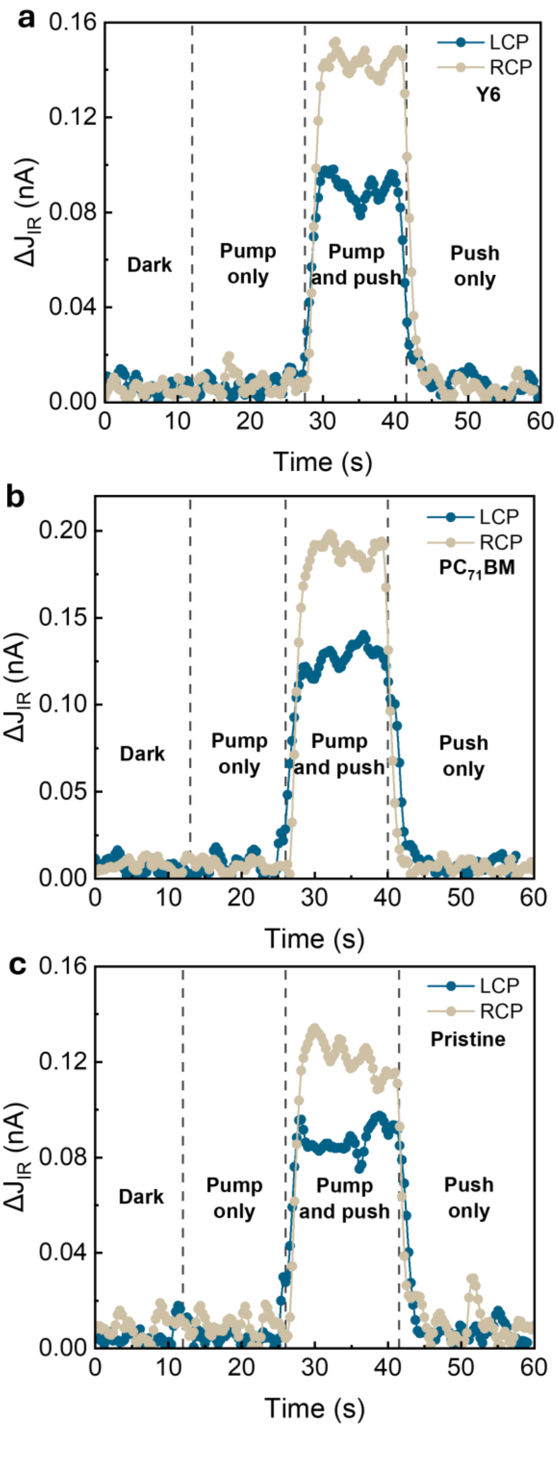

Supplementary Figure 18| PPPc measurements for CP-OPDs. a-c, ΔJ_{IR} responses in polarization-dependent cw-PPPc measurement for Y6 (**a**), $PC_{71}BM$-introduced CP-OPDs (**b**), and a pristine CP-OPD as a function of time (**c**).

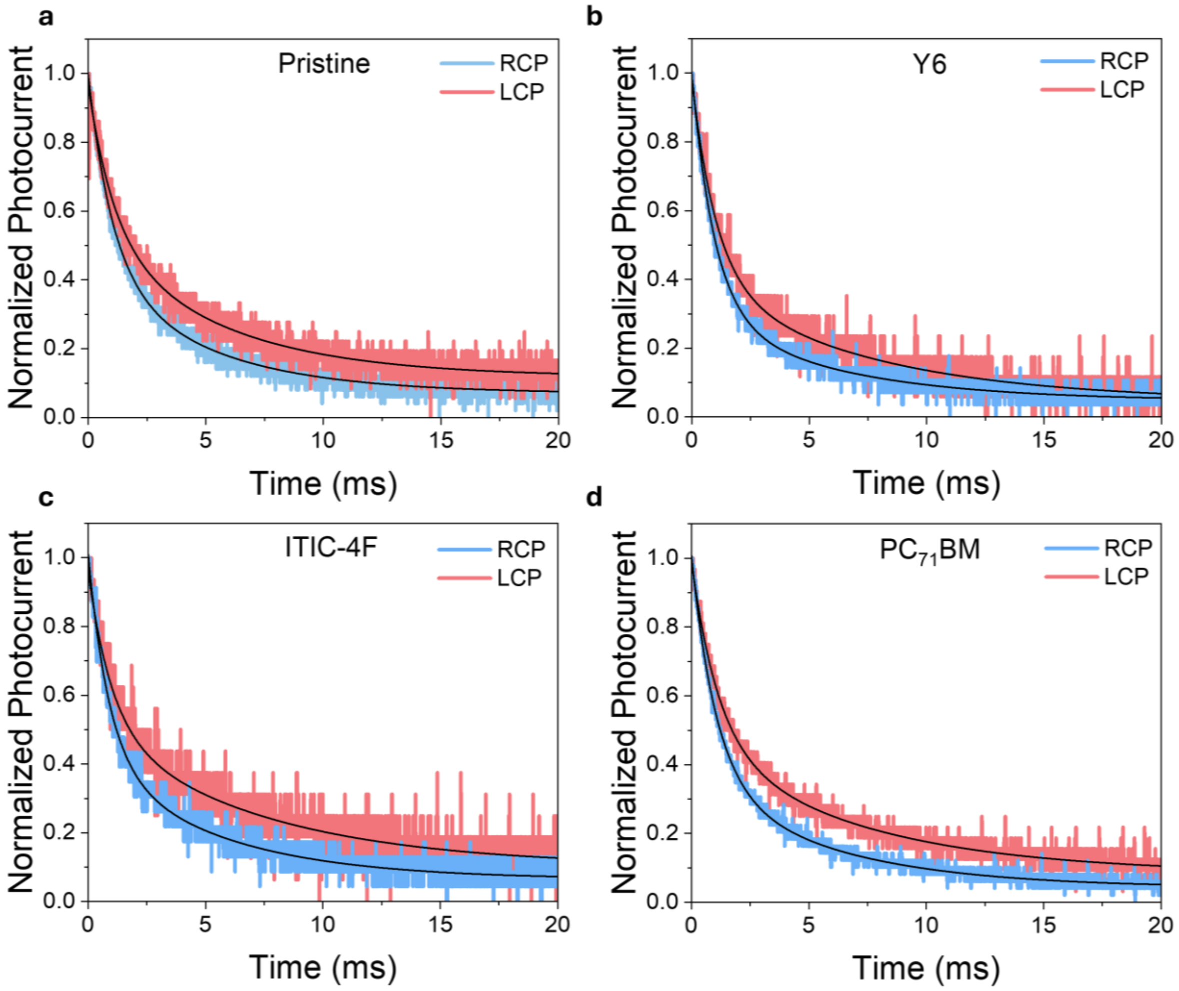

Supplementary Figure 19| TPC measurements. a-d, Polarization-dependent TPC decay curves of CP-OPDs without the acceptor (**a**) or with the introduction of Y6 (**b**), ITIC-4F (**c**), and $PC_{71}BM$ (**d**). Devices with the configuration of ITO/PEDOT:PSS/CHP:CI ([*P*]-aza[6]H)/C_{60}/Al were fabricated and measured under CP illumination at a wavelength of 470 nm.

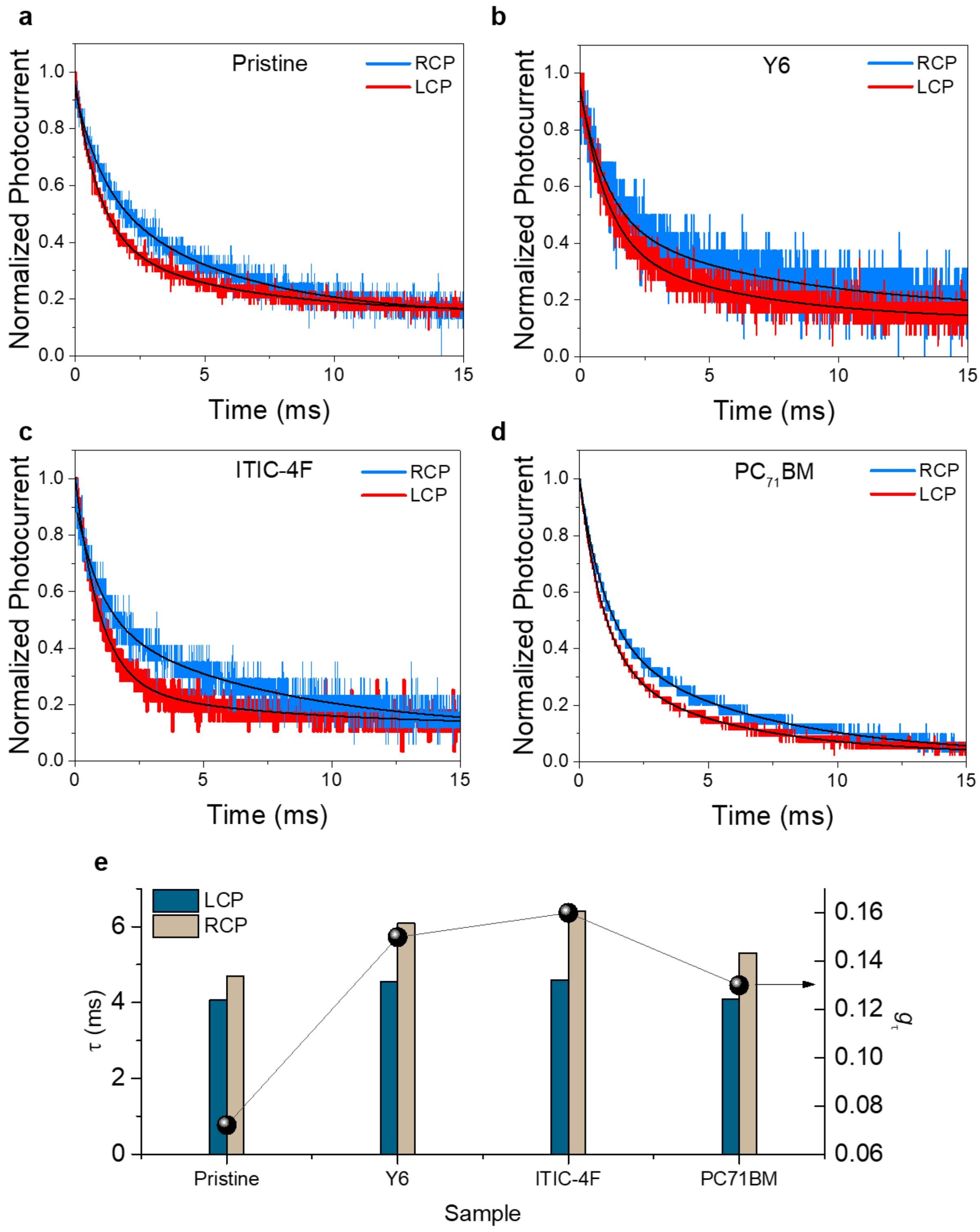

Supplementary Figure 20| TPC measurements with a different enantiomer of aza[6]H molecule. a-d, Polarization-dependent TPC decay curves of CP-OPDs without the acceptor (**a**) or with the introduction of Y6 (**b**), ITIC-4F (**c**), and $PC_{71}BM$ (**d**). **e**, Polarization-dependent τ for each type of CP-OPDs. Devices with the configuration of ITO/PEDOT:PSS/CHP:CI ([*M*]-aza[6]H)/C_{60}/Al were fabricated and measured under CP illumination at a wavelength of 470 nm. Fitted parameters for TPC analysis are shown in Supplementary Table 3.

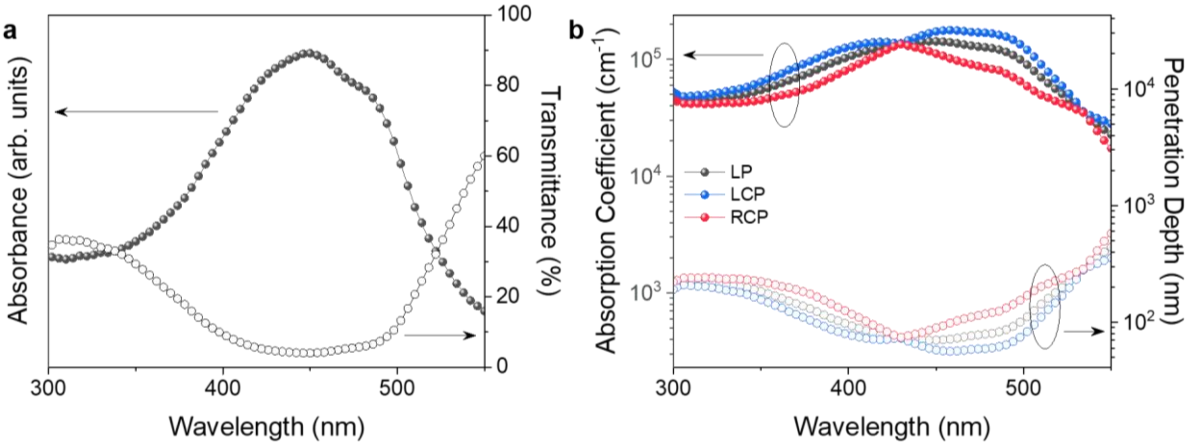

Supplementary Figure 21| Derivation of the optical penetration depth in a 220 nm thick CHP:CI film under CP light. a, Absorption spectrum and transmittance of thin CHP:CI film. **b**, Extracted absorption coefficient and corresponding penetration depth as a function of light polarization. Absorption coefficient (α) was derived by the equation, $\alpha = -\frac{\ln T}{x}$, where T is the transmittance and x is thickness of the film, respectively. Polarization-dependent optical penetration depth was calculated, considering g_{abs} spectrum of the film and equations, $g_{abs} = \frac{2(\alpha_L - \alpha_R)}{(\alpha_L + \alpha_R)}$ and $\alpha_{LP} = \frac{\alpha_L + \alpha_R}{2}$, where α_L, α_R, and α_{LP} denotes the absorption coefficient under LCP, RCP, linear polarized (LP) light illumination. Estimated optical penetration depths are 110.4 nm and 58.3 nm under RCP and LCP light at 470 nm, respectively. Absorption spectra are usually measured with unpolarized light, but here we assume linearly polarized light simply to account for the penetration depth. This calculation was based on the assumption that if the incident intensity (I_o) decreases due to a linear polarizer, the transmitted intensity (I_t) will decrease proportionally. Accordingly, the transmittance value (I_t/I_o) will remain at a constant level.

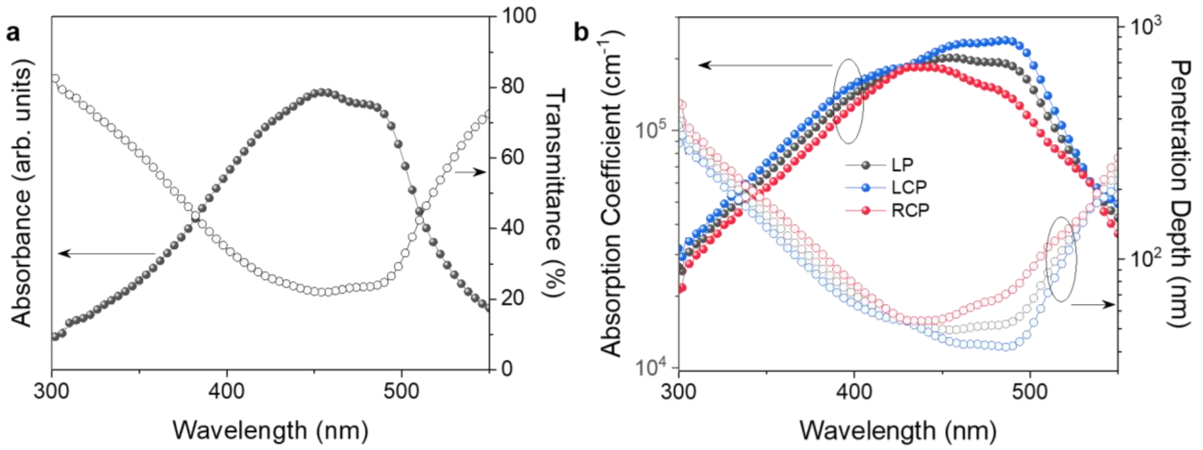

Supplementary Figure 22| Derivation of the optical penetration depth in a 75 nm thick CHP:CI film under CP light. a, Absorption spectrum and transmittance of thin CHP:CI film. **b**, Extracted absorption coefficient and corresponding penetration depth as a function of light polarization. Estimated optical penetration depths are 63.5 nm and 43.0 nm under RCP and LCP light at 470 nm, respectively.

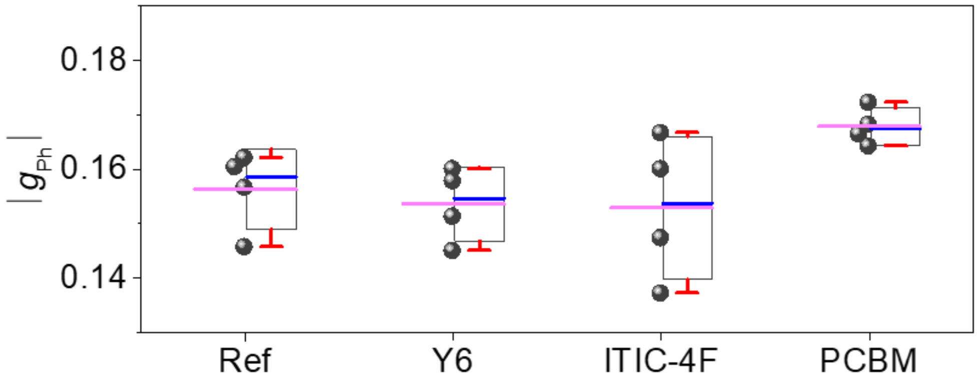

Supplementary Figure 23| Absolute g_{Ph} of CP-OPDs fabricated by the thinner chiral matrices (~ 75 nm) with the different type of acceptors. The thickness of the chiral active layers was adjusted by varying the spin-coating conditions. Devices with the configuration of ITO/PEDOT:PSS/CHP:CI/C_{60}/Al were fabricated and measured under CP illumination at a wavelength of 470 nm and a light intensity of 23.6 mW cm^{-2}. Box range is defined by standard deviation of g_{Ph} for 4 devices. Red, blue, and pink lines indicate whisker, median line, and mean line, respectively (pristine: median (0.15897), upper (0.16196) and lower quartiles (0.15130), upper (0.16219) and lower whiskers (0.14578), interquartile range (0.01066), no outliers; Y6: median (0.15366), upper (0.15956) and lower quartiles (0.14947), upper (0.16013) and lower whiskers (0.14509), interquartile range (0.01009), no outliers; ITIC-4F: median (0.15384), upper (0.16243) and lower quartiles (0.14335), upper (0.16677) and lower whiskers (0.13727), interquartile range (0.01908), no outliers; $PC_{71}BM$: median (0.16743), upper (0.17034) and lower quartiles (0.16546), upper (0.17237) and lower whiskers (0.16435), interquartile range (0.00488), no outliers).

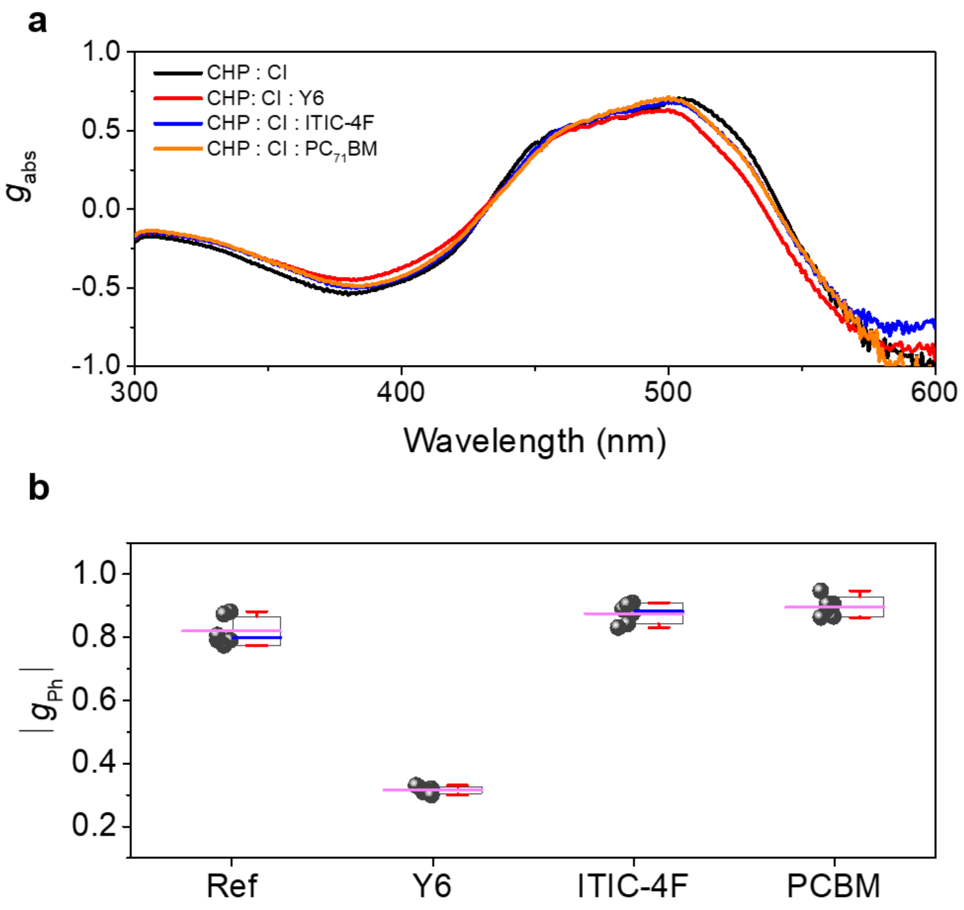

Supplementary Figure 24| g_{abs} spectra of chiral matrices and corresponding absolute g_{Ph} of CP-OPDs with the high doping concentration of acceptors (5 wt%). **a**, g_{abs} spectra of chiral matrices. **b**, corresponding absolute g_{Ph} of CP-OPDs with the high doping concentration of acceptors (5 wt%). Except the acceptor doping concentration, device fabrication and measurement conditions were identical to those used for the CP-OPDs with the low doping concentration of acceptors (1 wt%), with measurements performed at the same wavelength (470 nm) and light intensity (23.6 mW cm^{-2}). Box range is defined by standard deviation of g_{Ph} for 6 devices. Red, blue, and pink lines indicate whisker, median line, and mean line, respectively (pristine: median (0.80015), upper (0.85846) and lower quartiles (0.79125), upper (0.88137) and lower whiskers (0.77403), interquartile range (0.06721), no outliers; Y6: median (0.31639), upper (0.32754) and lower quartiles (0.31035), upper (0.33053) and lower whiskers (0.29927), interquartile range (0.01719), no outliers; ITIC-4F: median (0.88004), upper (0.90089) and lower quartiles (0.84384), upper (0.90952) and lower whiskers (0.83081), interquartile range (0.05705), no outliers; $PC_{71}BM$: median (0.88654), upper (0.90117) and lower quartiles (0.86769), upper (0.94764) and lower whiskers (0.86343), interquartile range (0.03348), no outliers).

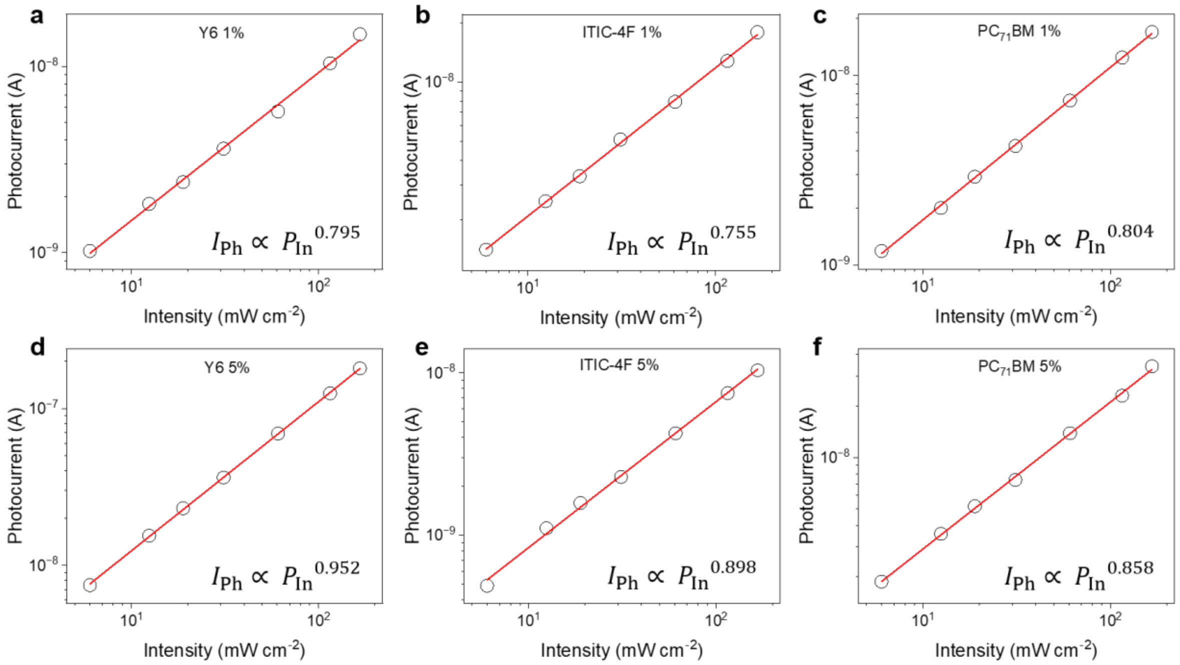

Supplementary Figure 25| Light intensity dependence of photocurrent (I_{Ph}) of CP-OPDs with the different concentration of acceptors. **a**, 1 wt% Y6. **b**, 1 wt% ITIC-4F. **c**, 1 wt% $PC_{71}BM$. **d**, 5 wt% Y6. **e**, 5 wt% ITIC-4F. **f**, 5 wt% $PC_{71}BM$. The measurement was performed at the wavelength of 470 nm (non-polarized) with various light intensities, which was modulated by ND filters. The I_{Ph} exhibits a power-law dependence on the incident light intensity (P_{In}), described by the relation $I_{Ph} \propto P_{In}^{\alpha}$.

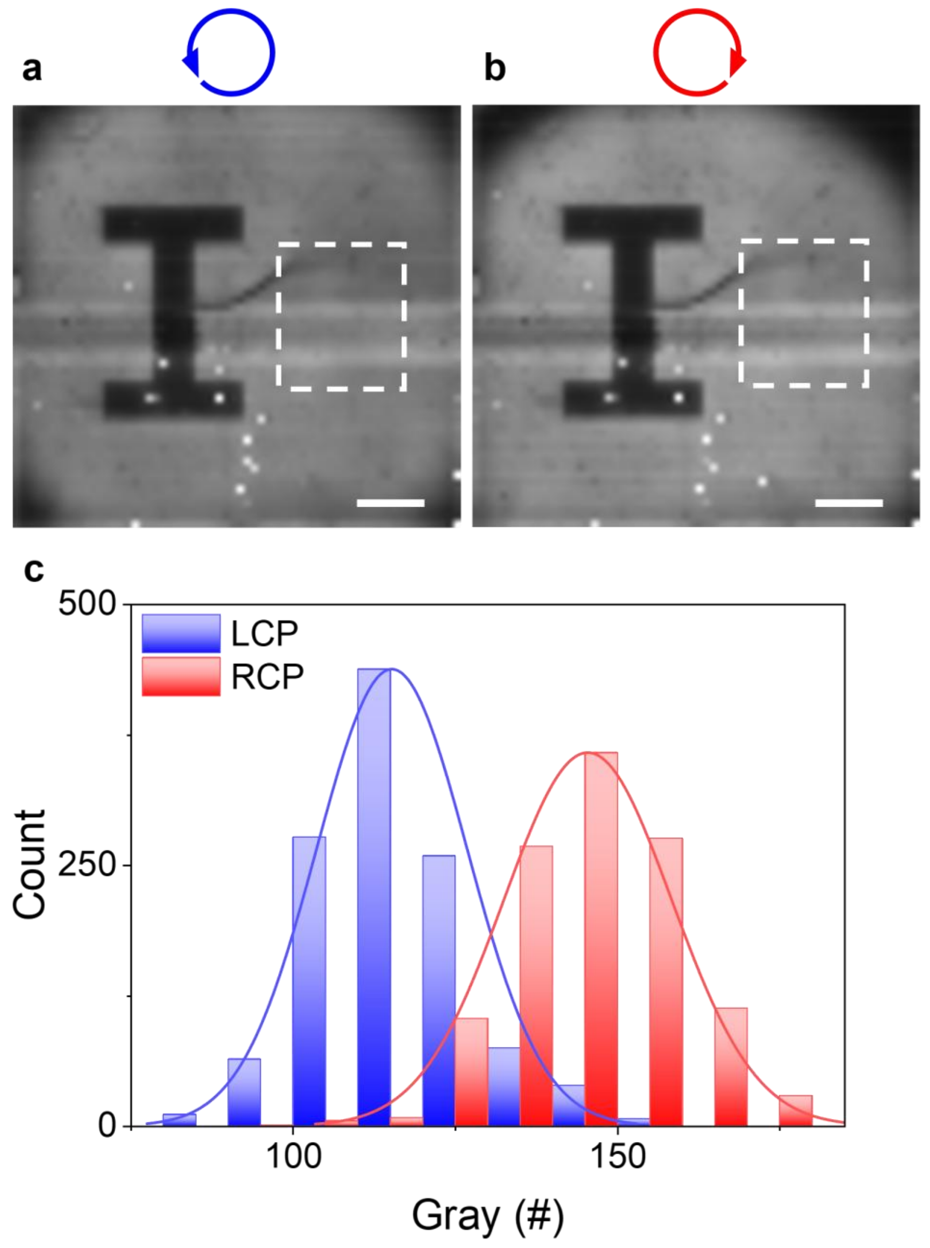

Supplementary Figure 26| Scanned images from an organic CP-imager. a, Scanned image under LCP light or **b**, RCP light with a wavelength of 470 nm. where a transparent mask with an opaque 'I'-shaped pattern ('I' part is opaque, and the other part is transparent), representing Imperial College London, was placed between the imager and the light source. **c**, Histogram exhibiting grayscale of dashed areas for each scanned image. Inset scale bars in all scanned images indicate 1 cm.

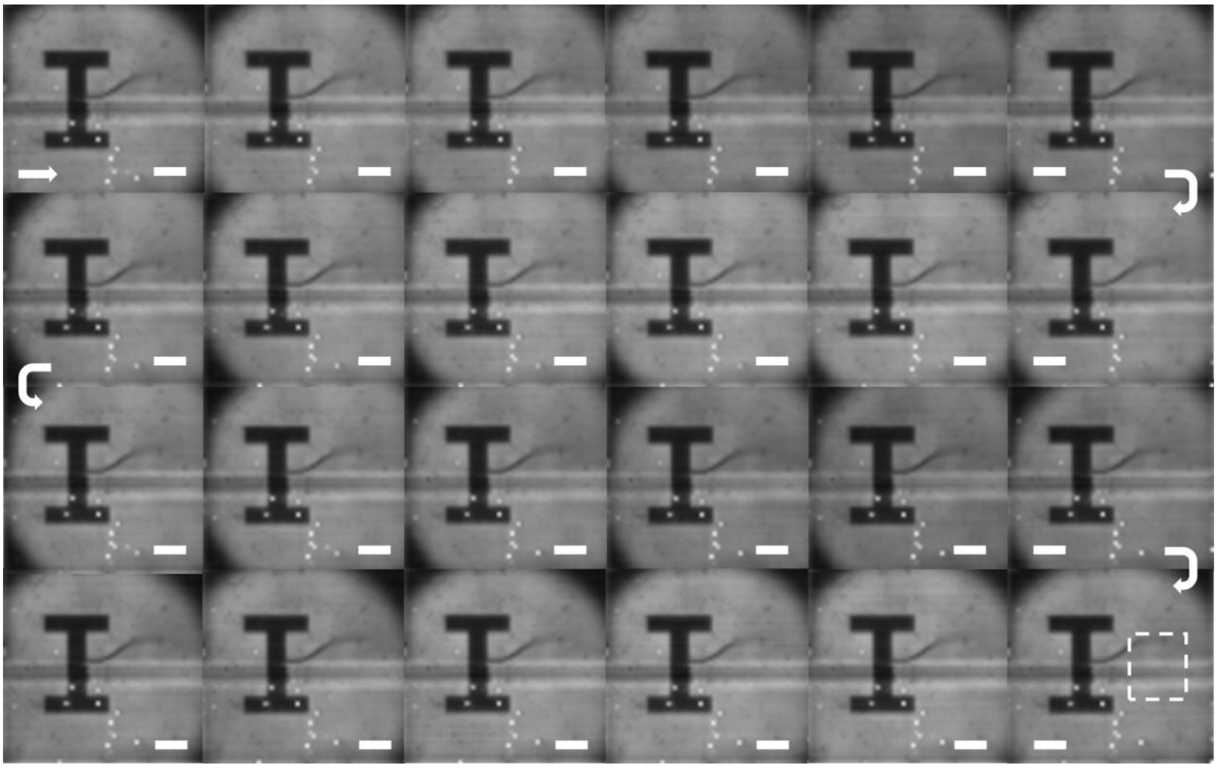

Supplementary Figure 27| Scanned images from an organic CP-imager. Scanned images with full polarization states, where the polarization state is controlled by adjusting the angle of the quarter-wave plate (QWP) from 0 ° to 345° with a wavelength of 470 nm. Grayscale are extracted from corresponding dashed areas for each image. A transparent mask with an opaque 'I'-shaped pattern ('I' part is opaque, and the other part is transparent), representing Imperial College London, was placed between the imager and the light source. Inset scale bars in all scanned images indicate 1 cm.

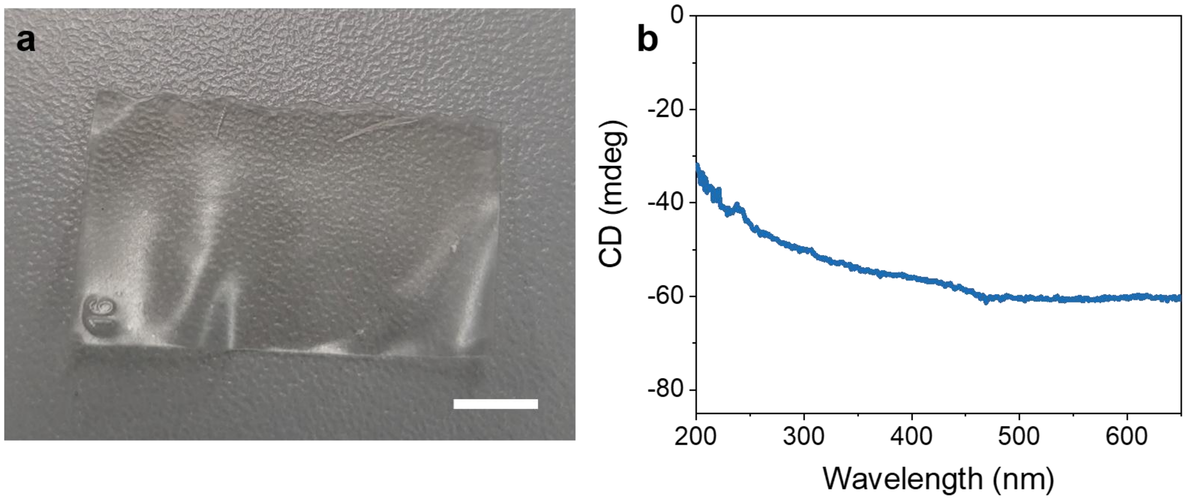

Supplementary Figure 28| Transparent CNCs film. a, Photograph of transparent CNCs film. **b**, CD spectrum of the CNCs film. The CNCs powder purchased from Cellulose Lab was dissolved in DI water (60 mg ml^{-1}) and drop-cast onto the cleaned fused-silica substrates. Drop-cast films were naturally dried for 48 hours at room temperature. An inset scale bar indicates 1 cm.

Supplementary Table 1. GIWAXS derived *d*-spacing calculated from chiral matrices.

Sample peak		*q* (Å $^{-1}$)	*d* (Å)
Pristine	Lamellar	0.37	17.0
	π-π	1.29	4.9
Y6	Lamellar	0.35	18.0
	π-π	1.28	4.9
ITIC-4F	Lamellar	0.32	19.6
	π-π	1.27	4.9
$PC_{71}BM$	Lamellar	0.32	19.6
	π-π	1.24	5.1

Supplementary Table 2. Summarized g_{Ph}, g_{abs}, and driving voltage (V_d) of CP-OPDs in previous studies.

Chiral Matrices	Absolute g_{abs}	Absolute g_{Ph}	V_d (V)	Detectivity (Jones)	Responsivity (AW^{-1})	Detecting λ (nm)	Device Type	Reference
aza[6]H	~10^{-3}	1.8	60	N/A	N/A	365	Transistor	8
CPDI-Ph	0.015	0.33	70	N/A	15.7	460	Transistor	9
F6BT	N/A	1.94	60	7.44×10^{7}	1.86×10^{-5}	450	Transistor	10
ClCPDI-Ph-CF	N/A	0.129	10	2.2×10^{16}	10^{3}	495	Transistor	11
Bis[60]PCBM enantiomer	0.0006	1.27	20	N/A	N/A	405	Transistor	12
3a-*P*/*M*	0.002	0.057	80	2.1×10^{10}	0.45	730	Transistor	13
Chiral naphthalene diimides	2.2×10^{-3}	0.05	30	3.9×10^{12}	314	385	Transistor	14
4CldiPDI	0.005	0.16	80	4.7×10^{11}	16.5	488	Transistor	15
C13P3.75:S6N	0.2	0.58	80	5×10^{11}	3.88	405	Transistor	16
NTPH-P:DPA	1.5×10^{-3}	0.24	60	1.33×10^{9}	0.233	556	Transistor	17
Metal organic framework (*R*-MeBINOL-PorDC)	N/A	4.3×10^{-4}	2	N/A	N/A	450	Photoconductor	18
P3CT:BN	N/A	0.1	5	N/A	N/A	375	Photoconductor	19
chiral covalent organic frameworks (CityU-7 and CityU-8)	2.3×10^{-4}	0.95	0.1	5.5×10^{8}	1	405	Photoconductor	20
chiral silver nanoclusters (Ag_7(R/S-DMA)$_2$(dpppy)$_3$(BF_4)$_3$)	N/A	0.18	1	1.00×10^{9}	5.92	520	Photoconductor	21
Chiral fluorene copolymer	0.06	0.017	0	N/A	N/A	543	Diode	22
(*S*,*S*)-ProSQ-C6:PCBM	0.09	0.1	0	N/A	N/A	543	Diode	23
F8T2:aza[6]H	0.03	0.41	0	N/A	N/A	510	Diode	24
DPP6T:PCBM	0.02	0.17	0	2.64×10^{10}	0.044	606	Diode	25
Chiral polythiophene nanowires	0.0016	0.047	0.4	1.25×10^{11}	0.123	560	Diode	26
ITIC:R5011	0.09	0.113	2	1.07×10^{13}	0.329	700	Diode	27
PM6:chiral BTP-4Cl	0.02	0.03	0	3×10^{11}	0.4	830	Diode	28
PM6:chiral BTP-4F	0.015	0.016	0	N/A	0.4	830	Diode	29
Chiral Polyacetylene	2.25×10^{-3}	0.08	0	3.15×10^{7}	0.18	365	Diode	30
PCDTPT:limonene	N/A	0.18	0	1.5×10^{12}	6	532	Diode	31
PCPDTTBTT:S5011	1.21	1.16	1	1.5×10^{11}	N/A	670	Diode	32
PCPDTTBTT:S5011:N2200	1	0.53	5	2.1×10^{11}	0.75	730	Diode	33
(+)/(−)-2NDI	0.06	1.67	50	8.25×10^{10}	13.76	450	Transistor	34
(*S*) or (*R*)-P(NDI2MH-T2)	1.38×10^{-2}	0.043	70	1.1×10^{12}	92	670	Transistor	35
F8T2:aza[6]H	0.7	1.19	1.5	1.01×10^{10}	3.84×10^{-3}	470	Diode	This work

Supplementary Table 3. Fitted parameters for TPC analysis of CP-OPDs depending on the handedness of 470 nm CP light. Biexponential equation was used to fit the decay curves as follows, $y = y_0 + A_1 \exp\left(-\frac{x}{\tau_1}\right) + A_2 \exp\left(-\frac{x}{\tau_2}\right)$.

Active layer (CHP : [*P*]-aza[6]H)		A_1	A_2	τ_1 (ms)	τ_2 (ms)	$<\tau>$ (ms)	$(\tau_L-\tau_R)/(\tau_L+\tau_R)$
Pristine	LCP	0.49	0.51	1.06	5.30	4.62	8.58×10^{-2}
	RCP	0.60	0.40	1.02	4.80	3.89	
ITIC-4F	LCP	0.53	0.47	9.76×10^{-1}	7.03	6.20	1.74×10^{-1}
	RCP	0.62	0.38	8.82×10^{-1}	5.31	4.36	
Y6	LCP	0.61	0.39	1.00	6.99	5.90	1.56×10^{-1}
	RCP	0.72	0.28	9.58×10^{-1}	5.77	4.31	
$PC_{71}BM$	LCP	0.57	0.43	1.12	6.89	5.87	1.36×10^{-1}
	RCP	0.67	0.33	1.04	5.71	4.46	
Active layer (CHP : [*M*]-aza[6]H)		A_1	A_2	τ_1 (ms)	τ_2 (ms)	$<\tau>$ (ms)	$(\tau_L-\tau_R)/(\tau_L+\tau_R)$
Pristine	LCP	0.66	0.34	9.09×10^{-1}	5.16	4.07	7.18×10^{-2}
	RCP	0.40	0.60	9.89×10^{-1}	5.17	4.70	
ITIC-4F	LCP	0.83	0.17	9.54×10^{-1}	7.09	4.60	1.64×10^{-1}
	RCP	0.50	0.50	9.53×10^{-1}	7.15	6.41	
Y6	LCP	0.66	0.34	9.99×10^{-1}	5.74	4.55	1.45×10^{-1}
	RCP	0.58	0.42	1.04	7.10	6.09	
$PC_{71}BM$	LCP	0.66	0.34	9.29×10^{-1}	5.20	4.09	1.30×10^{-1}
	RCP	0.56	0.44	9.95×10^{-1}	6.21	5.31	

Supplementary references

1 Owens, D. K. & Wendt, R. C. Estimation of the surface free energy of polymers. *J. Appl. Polym. Sci.* **13**, 1741 (1969)

2 Zhao, T., Xia, K., Natali, D. & Pecunia, V. Solution-Based Integration of Vertically Stacked Organic Photodetectors Toward Easy-To-Fabricate Filterless Multi-Color Light Sensors. *Adv. Opt. Mater.* **10**, 2200862 (2022).

3 Furlan, F. *et al.* Electrical control of photon spin angular momentum in organic electroluminescent materials. *ChemRxiv.* doi:10.26434/chemrxiv-2025-rbqh1 (2025).

4 R. Luginbuhl, B. *et al.* Resolving Atomic-Scale Interactions in Nonfullerene Acceptor Organic Solar Cells with Solid-State NMR Spectroscopy, Crystallographic Modelling, and Molecular Dynamics Simulations. *Adv. Mater.* **34**, 2105943 (2022).

5 Yu, R., Yao, H. & Hou, J. Recent Progress in Ternary Organic Solar Cells Based on Nonfullerene Acceptors. *Adv. Energy Mater.* **8**, 1702814 (2018).

6 Pan, M.-A. *et al.* 16.7%-efficiency ternary blended organic photovoltaic cells with PCBM as the acceptor additive to increase the open-circuit voltage and phase purity. *J. Mater. Chem. A* **7**, 20713-20722 (2019).

7 Jain, A. *et al.* Trap filled limit voltage (V_{TFL}) and V^2 law in space charge limited currents. *J. Appl. Phys.* **102**, 094505 (2007).

8 Yang, Y., da Costa, R. C., Fuchter, M. J. & Campbell, A. J. Circularly polarized light detection by a chiral organic semiconductor transistor. *Nat. Photonics* **7**, 634-638 (2013).

9 Shang, X. *et al.* Supramolecular Nanostructures of Chiral Perylene Diimides with Amplified Chirality for High-Performance Chiroptical Sensing. *Adv. Mater.* **29**, 1605828 (2017).

10 Cheng, J. *et al.* Enabling discrimination capability in an achiral F6BT-based organic semiconductor transistor via circularly polarized light induction. *J. Mater. Chem. C* **8**, 9271-9275 (2020).

11 Shang, X. *et al.* Surface-Doped Quasi-2D Chiral Organic Single Crystals for Chiroptical Sensing. *ACS Nano* **14**, 14146-14156 (2020).

12 Shi, W. *et al.* Fullerene Desymmetrization as a Means to Achieve Single-Enantiomer Electron Acceptors with Maximized Chiroptical Responsiveness. *Adv. Mater.* **33**, 2004115 (2021).

13 Zhang, L. *et al.* π-Extended perylene diimide double-heterohelicenes as ambipolar organic semiconductors for broadband circularly polarized light detection. *Nat. Commun.* **12**, 142 (2021).

14 Kwon, Y., Jung, J.-Y., Lee, W. B. & Oh, J. H. Axially Chiral Organic Semiconductors for Visible-Blind UV-Selective Circularly Polarized Light Detection. *Adv. Sci.* **11**, 2308262 (2024).

15 Gao, K. *et al.* Reversal of chirality in solutions and aggregates of chiral tetrachlorinated diperylene diimides towards efficient circularly polarized light detection. *Mater. Horiz.* **12**, 1903-1912 (2025).

16 Wu, X., Liu, J., Xu, Y., Qiu, L. & Wang, X. High-performance circularly polarized photodetectors based on chiral transfer of achiral poly(9,9-dioctylfluorene). *J. Mater. Chem. C* **13**, 628-638 (2025).

17 Zhu, D. *et al.* Organic donor-acceptor heterojunctions for high performance circularly polarized light detection. *Nat. Commun.* **13**, 3454 (2022).

18 Li, C. *et al.* Twisting of Porphyrin by Assembly in a Metal-Organic Framework yielding Chiral Photoconducting Films for Circularly-Polarized-Light Detection. *Angew. Chem., Int. Ed.* **62**, e202217377 (2023).

19 Kim, N. Y. *et al.* Chiroptical-Conjugated Polymer/Chiral Small Molecule Hybrid Thin Films for Circularly Polarized Light-Detecting Heterojunction Devices. *Adv. Funct. Mater.* **29**, 1808668 (2019).

20 Gu, Q. *et al.* Constructing Chiral Covalent-Organic Frameworks for Circularly Polarized Light Detection. *Adv. Mater.* **36**, 2306414 (2024).
21 He, W. M. *et al.* Atomically precise chiral metal nanoclusters for circularly polarized light detection. *Angew. Chem.* **136**, e202407887 (2024).
22 Gilot, J. *et al.* Polymer photovoltaic cells sensitive to the circular polarization of light. *Adv. Mater.* **22**, E131-E134 (2010).
23 Schulz, M. *et al.* Chiral Excitonic Organic Photodiodes for Direct Detection of Circular Polarized Light. *Adv. Funct. Mater.* **29**, 1900684 (2019).
24 Ward, M. D. *et al.* Highly Selective High-Speed Circularly Polarized Photodiodes Based on π-Conjugated Polymers. *Adv. Opt. Mater.* **10**, 2101044 (2022).
25 Liu, L. *et al.* Building Supramolecular Chirality in Bulk Heterojunctions Enables Amplified Dissymmetry Current for High-Performing Circularly Polarized Light Detection. *ACS Mater. Lett.* **4**, 401-409 (2022).
26 Wang, Z., Gao, M., Hao, X. & Qin, W. Helical-chiroptical nanowires generated orbital angular momentum for the detection of circularly polarized light. *Appl. Phys. Lett.* **116** (2020).
27 Wan, L. *et al.* Sensitive near-infrared circularly polarized light detection via non-fullerene acceptor blends. *Nat. Photonics* **17**, 649-655 (2023).
28 Liu, L. *et al.* Chiral Non-Fullerene Acceptor Enriched Bulk Heterojunctions Enable High-Performance Near-Infrared Circularly Polarized Light Detection. *Small* **18**, 2202941 (2022).
29 Liu, L., Wei, Z. & Meskers, S. C. J. Semi-Transparent, Chiral Organic Photodiodes with Incident Direction-Dependent Selectivity for Circularly Polarized Light. *Adv. Mater.* **35**, 2209730 (2023).
30 Zhang, Y. *et al.* Chiral Polyacetylene with Thermally Activated Delayed Fluorescence Feature for High-Performance Circularly Polarized Light Detection. *Chem. Mater.* **36**, 3369-3380 (2024).
31 Hu, R., Lu, X., Hao, X. & Qin, W. An Organic Chiroptical Detector Favoring Circularly Polarized Light Detection from Near-Infrared to Ultraviolet and Magnetic-Field-Amplifying Dissymmetry in Detectivity. *Adv. Mater.* **35**, 2211935 (2023).
32 Song, I. *et al.* Helical polymers for dissymmetric circularly polarized light imaging. *Nature* **617**, 92-99 (2023).
33 Song, I. *et al.* Helical donor–acceptor bulk heterojunctions for dissymmetric circularly polarized light detection. *Chem. Eng. J.* **505**, 158991 (2025).
34 Zhuo, H. *et al.* Group theory-guided materials design of chiral organic semiconductors for high-performance circularly polarized light detection. *Matter* **8**, 102371 (2025).
35 Gao, K. *et al.* High-performance flexible circularly polarized light photodetectors based on chiral n-type naphthalenediimide-bithiophene polymers. *npj Flex. Electron.* **9**, 83 (2025).
