# Supplementary material for: Trap-assisted circularly polarized organic photodetector
Source: Nat Commun. 2026 Apr 25;17:5710. doi: 10.1038/s41467-026-72474-w (PMC13319231; doi:10.1038/s41467-026-72474-w)
Supplement: Supplementary file 2 — Transparent Peer Review file [file 41467_2026_72474_MOESM2_ESM.pdf]

nature portfolio

Peer Review File

Trap-assisted circularly polarized organic photodetector

Corresponding Author: Professor Matthew Fuchter

This file contains all reviewer reports in order by version, followed by all author rebuttals in order by version.

Version 0:

Reviewer comments:

Reviewer #1

(Remarks to the Author)
Comments on "Trap-Assisted Circularly Polarized Organic Photodetector"
In the manuscript entitled Trap-Assisted Circularly Polarized Organic Photodetector, the authors aim to demonstrate that carrier traps can enhance the asymmetric photocurrent in chiral organic photodetectors (OPDs). An interesting result was observed: the gPh of CP-OPDs was enhanced without modifying the gabs. However, the current version of the paper needs to be further improved to address the following comments:
1. The manuscript relies excessively on speculative claims that lack either experimental data or rigorous theoretical support. A particularly notable example appears in Line 199, where the authors state: "We hypothesised that the acceptor does not form a continuous electron transport network, but instead acts as a trap site that modifies the charge extraction process." For such a core premise of the study, a mere hypothesis is insufficient; conclusive experimental evidence demonstrating that the acceptors function as traps (rather than alternatively provide donor-acceptor interface or act as charge-transport components) is imperative.
2. Continuous-wave photoinduced polarization charge (cw-PPPc) measurements were employed in an attempt to directly observe trapped charge carriers in the CP-OPDs. However, the study fails to quantify critical trap-related parameters—including trap energy depth, trap level dispersion, and energy barriers governing carrier mobility—that are essential to establishing a causal link between trapping effects and CP selectivity. Consequently, there is no quantitative evidence to support the claimed correlation between carrier trapping and the handedness of circularly polarized light (CPL) in devices with or without achiral acceptors.
3. Serious concerns arise regarding the reliability of the circular dichroism (CD) measurements and their subsequent analysis. In Figure 2, the absorption spectra of the acceptors are not provided, making it impossible to correlate the observed CD signals with specific electronic transitions of these acceptors. Given the low acceptor loading, their contribution to the overall absorption is negligible—raising the strong possibility that the measured g values for the acceptors are experimental artifacts.
Furthermore, comparing the CD spectra in Figure 2a/d, 2b/e, and 2c/f reveals that increasing the acceptor loading from 1% to 5% induces significant changes in CD band shape. These changes show no clear correlation with the acceptors' absorption features (i.e., the associated electronic transitions remain undefined). Collectively, these issues cast substantial doubt on the validity of the CD measurement data and the corresponding analytical interpretations.
4. The mechanism underlying the structure-chirality correlation in the studied systems remains poorly characterized and analyzed. Solution-state diffusion-ordered nuclear magnetic resonance (DOSY) spectroscopy was used to investigate interactions between [P]-aza[6]H (CI) and the acceptors, but this data is insufficient to confirm the miscibility of acceptors with the CHP:CI blend. Notably, the polymer CHP constitutes a major component of the blend, yet the DOSY analysis fails to account for interactions involving CHP—whether with CI or the acceptors.
5. The highest gPh is obtained under reverse bias. The reverse bias may induce the artifacts when calculating the gPh. The intrinsic gph without bias needs to be compared and analyzed.

Reviewer #2

(Remarks to the Author)
In this manuscript, the authors explore the fundamental question of "potential mechanisms for asymmetric enhancement." Through detailed investigations in materials science and advanced spectroscopy, they reveal the crucial role of asymmetric trap dynamics in amplifying the gph within CP OPDs. This approach enabled the realization of a large area organic circularly polarized imaging system, demonstrating its potential for real time spatiotemporal visualization of optical chirality

and CNC assisted pattern encryption. However, certain vague descriptions—particularly the lack of clear core arguments—limit the paper's practical applicability. The following questions and recommendations should be addressed:
1. On page 4, the authors state: "We bridge this knowledge gap by incorporating a selection of acceptors into the chiral host polymer (CHP):CI blend and investigating CP OPD performance." Please elaborate on the rationale for selecting these specific acceptors and the CHP:CI blend. Why is this system expected to "bridge this knowledge gap"? Relying solely on prior team results to justify the choice appears too vague.
2. It's reported that adding an acceptor to the [P] aza[6]H system weakens the device's circularly polarized photocurrent response, with a significantly larger effect on LCP than on RCP (Figure 1e). However, when [P] aza[6]H is replaced with [M] aza[6]H, the acceptor doped devices show varied trends in RCP and LCP response depending on the acceptor type, without a clearly dominant polarization effect (Supplementary Figs 1 and 2). This observation seems inconsistent with the authors' core argument and requires explanation.
3. It is recommended to provide CD spectra of the neat CHP:CI film (without acceptor) for comparison. The authors should also discuss in detail how acceptor incorporation affects CD signal intensity and corresponding wavelengths. Furthermore, why do the CD signals for some acceptor films increase (Figures 2a and 2d) while others decrease (Figures 2b, 2e, 2c and 2f) as the acceptor content rises from 1 wt% to 5 wt%?
4. The authors note: "When the acceptor concentration was increased to 5 wt%, ITIC 4F exhibited a more pronounced CD with bisignate Cotton effects, indicating significant perturbation of ITIC 4F molecules by the CHP:CI thin film. This effect was more pronounced than that observed with the Y6 introduced film (Fig. 2d–f)." However, the provided figures do not show that the ITIC 4F system yields the strongest CD signal at either low or high concentration.
5. In Fig. 1a, the device electrode is labeled as Al, whereas the Methods section describes it as Ag. Please verify and correct the device structure accordingly.
6. The authors did not include the [M] aza[6]H system in their core investigations on circularly polarized light detection and mechanism analysis. This omission leaves a critical gap in comparative evidence. It is recommended to supplement the study with experiments on the [M] aza[6]H system, which would help strengthen the central argument regarding the role of molecular chirality in the observed phenomena.

Reviewer #3

(Remarks to the Author)
In this work, the authors reported the trap-assisted circularly polarized organic photodetectors (CP-OPDs) based on π-conjugated polymer, helicene and small molecular dopant, such as PCBM, Y6 and ITIC-4F. By adopting the blend systems with small amount of these achiral acceptors, the authors achieved asymmetric trap filling that depends on the handedness of circularly polarized light. The modulated trap dynamics further enhance the CPL selectivity during charge extraction. Moreover, the authors fabricated a 4096-pixel large-area organic CP imaging system and demonstrated its potential for real-time visual encryption. Overall, this manuscript is well-organized, and the conclusion is strongly supported by the data. Therefore, I recommend to accept this work after addressing the below minor issues.
1. Why are different excitation wavelengths used (405 nm for PPPc experiments vs. 470 nm for CPL detection)? Could this affect the correlation between the proposed trap mechanism and CPL performance?
2. As PPPc only measures trapped carrier concentration, it remains unclear whether the observed discrepancy arises from differences in carrier generation or trap capture probability.
3. The DOSY experiments were performed in solution, while the devices are based on thin films. Could this state difference impact the correlation between the observed intermolecular interactions and the CPL device performance?
4. Please compare the device performance with that of state-of-the-art photodiode-based CP-OPDs to clarify the advantages.
5. The latest progress of CP-OPDs should be included (DOI: 10.1016/j.matt.2025.102371; 10.1038/s41528-025-00443-2).
6. Please address whether the strategy of enhancing gph via the trap strategy sacrifices other device performance metrics (e.g., response speed, detectivity, stability) and provide a balanced assessment of the strategy in the manuscript.
7. There are spelling errors in the manuscript (e.g., "afftect" should be "affect"), and it is recommended that the authors undergo professional English language proofreading before submission.

Version 1:

Reviewer comments:

Reviewer #1

(Remarks to the Author)
The manuscript has been revised according to the reviewers' comments and could be acceptable for publication.

Reviewer #2

(Remarks to the Author)
All the issues have been well addressed, so I recommend accepting this paper for publication.

Reviewer #3

(Remarks to the Author)
The authors have properly revised the manuscript according to the review comments. The revised version is acceptable. I

recommend acceptance of this manuscript.

Open Access This Peer Review File is licensed under a Creative Commons Attribution 4.0 International License, which permits use, sharing, adaptation, distribution and reproduction in any medium or format, as long as you give appropriate credit to the original author(s) and the source, provide a link to the Creative Commons license, and indicate if changes were made.
In cases where reviewers are anonymous, credit should be given to 'Anonymous Referee' and the source.
The images or other third party material in this Peer Review File are included in the article's Creative Commons license, unless indicated otherwise in a credit line to the material. If material is not included in the article's Creative Commons license and your intended use is not permitted by statutory regulation or exceeds the permitted use, you will need to obtain permission directly from the copyright holder.
To view a copy of this license, visit https://creativecommons.org/licenses/by/4.0/

We have carefully studied the reviewers' and editor's comments, and conducted additional experiments to fully address their comments and concerns. The manuscript has now been thoroughly revised in accordance to these comments and suggestions, and we thank the reviewers for improving the manuscript. **We have highlighted the changes and additions in neon blue in the revised manuscript and supplementary information (SI)**.

Reviewer #1

In the manuscript entitled Trap-Assisted Circularly Polarized Organic Photodetector, the authors aim to demonstrate that carrier traps can enhance the asymmetric photocurrent in chiral organic photodetectors (OPDs). An interesting result was observed: the gPh of CP-OPDs was enhanced without modifying the gabs. However, the current version of the paper needs to be further improved to address the following comments:

Response:

Thank you for your constructive comments. We are grateful to the reviewer for their constructive and thoughtful comments, which were very helpful for improving our manuscript.

Comment 1: The manuscript relies excessively on speculative claims that lack either experimental data or rigorous theoretical support. A particularly notable example appears in Line 199, where the authors state: "We hypothesised that the acceptor does not form a continuous electron transport network, but instead acts as a trap site that modifies the charge extraction process." For such a core premise of the study, a mere hypothesis is insufficient; conclusive experimental evidence demonstrating that the acceptors function as traps (rather than alternatively provide donor-acceptor interface or act as charge-transport components) is imperative.

Response:

Thank you for this comment. Our assumption that the introduced acceptors do not form a continuous electron-transport network, but instead function as isolated trap sites, is firmly grounded in the extensive prior literature on trap-assisted photomultiplication-type organic photodetectors. It is well established that when acceptors are incorporated into a polymer host at very low concentrations (e.g. 1% by weight), they predominantly act as charge traps rather than forming percolated transport pathways (*Nanoscale* 2020, 12, 1091–1099; *Nano Lett.* 2017, 17, 1995–2002; *Sci. Rep.* 2015, 5, 9181; *Phys. Chem. Chem. Phys.* 2015, 17, 30712–30720; *Adv. Opt. Mater.* 2018, 6, 1800001; *Chem. Eng. J.* 2022, 435, 134973).

More specifically, multiple studies have demonstrated that introducing trace amounts of PCBM into polymer matrices does not generate a continuous electron-conduction network. Instead, PCBM forms spatially isolated islands that serve as electron-trapping centers (*ACS Appl. Mater. Interfaces* 2015, 7, 5890–5897; *ACS Appl. Mater. Interfaces* 2015, 7, 22660–22668; *Adv. Physics Res.* 2023, 2, 2200075). Importantly, analogous behavior has also been reported for non-fullerene acceptors, including Y6 (*Adv. Funct. Mater.* 2025, e18210), IDIC (*Adv. Mater.* 2024, 36, 2404597), and ITIC (*Adv. Opt. Mater.* 2016, 4, 1711–1717), where similarly low loading leads to trap formation rather than facilitating long-range electron transport.

Taken together, these studies conclusively show that small-molecule acceptors such as Y6, ITIC, IDIC, and PCBM when incorporated into host polymers at concentrations comparable to those used here (~1 wt%), act primarily as electron-trapping sites rather than forming continuous charge-transport pathways. The consistency across material classes and device architectures strongly supports our assertion that low loadings of NFAs and PCBM in our system function as extrinsic traps.

Our experimental observations further corroborate this conclusion. If the addition of 1 wt% acceptor was to establish continuous electron-transport pathways, one would expect a clear enhancement in photocurrent, external quantum efficiency, and responsivity. In contrast, we observe slightly reduced current density, EQE, and responsivity upon acceptor incorporation (Fig. 1e–g; Supplementary Figs. 2, 7, and 8). This device-level behavior is inconsistent with percolated electron conduction and instead fully consistent with the formation of trapping centers.

Nonetheless, to directly verify the trapping role of the introduced acceptors in our study, we performed additional space-charge-limited current (SCLC) measurements. Electron-only devices were fabricated to isolate electron transport and quantitatively probe electron trap density (Supplementary Fig. 17 in the revised SI). The SCLC analysis reveals a pronounced increase in electron trap density following acceptor incorporation. Because electrical current in these electron-only devices arises exclusively from electron transport, the observed increase in trap density provides direct experimental evidence that the introduced acceptors act as electron traps rather than forming a continuous electron-transport network.

We updated the data and interpretation in the revised manuscript as follows:

(Page 10 in revised manuscript) This was confirmed by space-charge-limited current (SCLC) measurements on electron-only devices with the introduction of acceptors, showing that the addition of 1 wt% acceptors increased the electron trap density (Supplementary Fig. 17).

(Page S18 in revised SI)

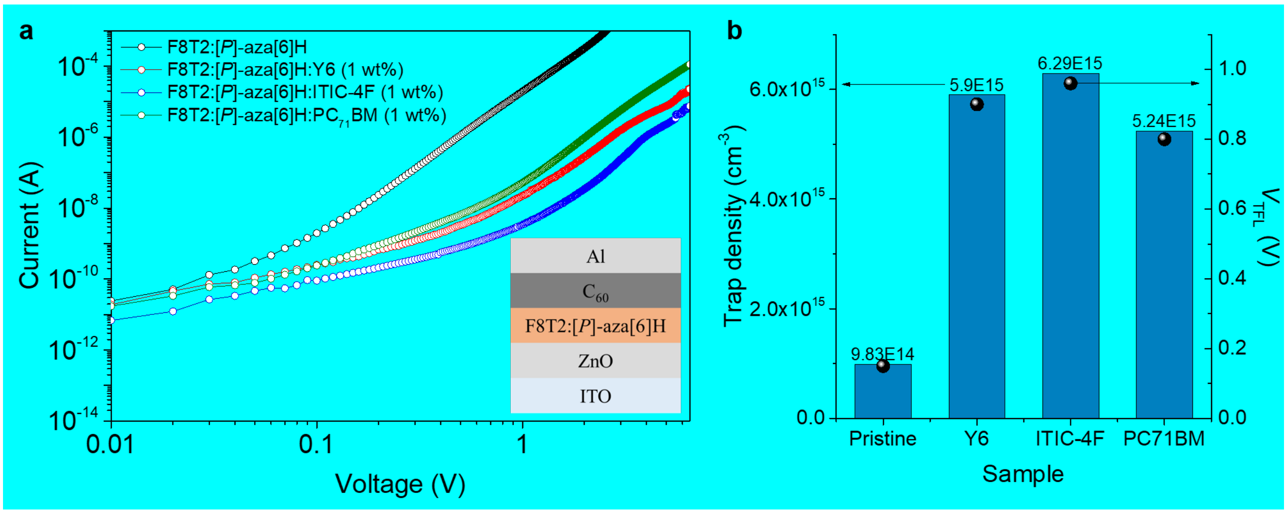

Supplementary Figure 17| SCLC measurements of electron-only devices. a, *I–V* curves of electron-only devices without or with acceptors and inset device configuration is used for electron-

only device. **b,** Electron trap density (left-axis) was calculated by using trap-filled limit voltage (V_{TFL}) value (right-axis) based on SCLC technique[7].

Comment 2: Continuous-wave photoinduced polarization charge (cw-PPPc) measurements were employed in an attempt to directly observe trapped charge carriers in the CP-OPDs. However, the study fails to quantify critical trap-related parameters—including trap energy depth, trap level dispersion, and energy barriers governing carrier mobility—that are essential to establishing a causal link between trapping effects and CP selectivity. Consequently, there is no quantitative evidence to support the claimed correlation between carrier trapping and the handedness of circularly polarized light (CPL) in devices with or without achiral acceptors.

Response:

We thank the reviewer for this comment. Whether the acceptor serves as an electron or hole trap is determined by the relative HOMO/LUMO energy alignment between the host polymer and the acceptor. In most reported systems, electrons in the host polymer transfer to the lower-lying LUMO level of the sparsely distributed acceptor molecules. Because a continuous electron-transport network cannot be formed at such a low loading (1 wt%), the transferred electrons remain localized on the acceptor and therefore do not contribute to the electrical current (*Nat. Commun.* 2021, 12, 4259; *Nano Lett*. 2017, 17, 1995–2002; *Adv. Opt. Mater*. 2018, 6, 1800001).

Importantly, these traps originate from the intentionally introduced acceptors rather than from intrinsic defects in the host polymer. Consequently, the energetic property of the trap is governed directly by the LUMO level of the acceptor molecule itself. In this framework, the trap depth can be defined simply as the energy offset between the LUMO of the host polymer and that of the acceptor. Because this offset determines the energetic barrier for electron detrapping, the trap characteristics can be considered to follow the acceptor LUMO level and its associated density of states. This interpretation is valid specifically because the traps observed here are extrinsic in nature: electrons are driven from the host polymer into the acceptor LUMO, where they are immobilized and recombined.

In studies in which a very small amount of acceptor is intentionally introduced to create trap states, this description has been consistently adopted. In these reports, the trap depth is explicitly described as the LUMO–LUMO offset between host and acceptor (*Adv. Opt. Mater.* 2018, 6, 1800001; *Appl. Phys. Lett.* 2023, 123, 111101; *J. Mater. Chem. C* 2020, 8, 14049–14055; *Nanoscale* 2020, 12, 1091–1099; *J. Phys. Chem. Lett.* 2022, 13, 10577–10588).

For this reason, we did not perform additional independent experiments to extract trap depths, as they are directly dictated by the well-established energy-level offsets between F8T2 and the acceptors used in this work. The relevant energy levels have been widely reported in the literature (Supplementary Fig. 16). Based on these values, the trap depths are defined as shown in Fig. R1. Electrons transferred from the host polymer to the acceptor thus experience a sufficiently deep energetic well to suppress thermally activated detrapping ($k_BT \approx 0.026$ eV) back into the host LUMO.

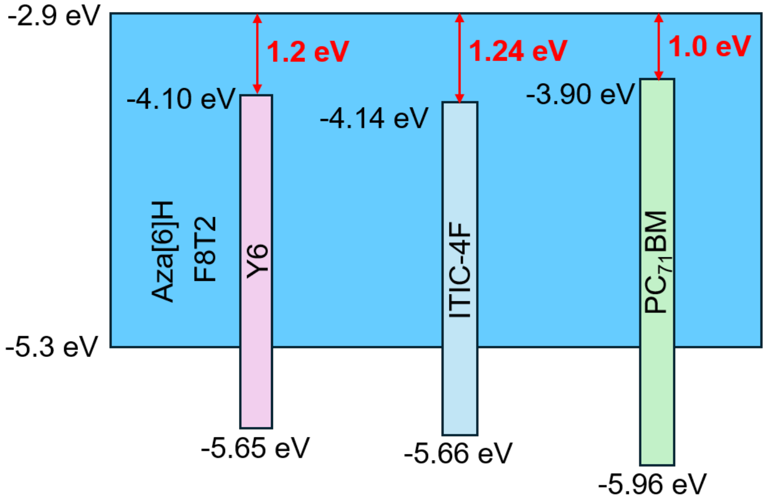

Fig. R1 Trap depths information depending on the added acceptors.

We further emphasize that the primary objective of this study is not to provide an exhaustive quantitative analysis of the trap physics itself in this blend material, but rather to demonstrate that introducing a small amount (1 wt%) of acceptor can induce strongly *asymmetric* trapping in CP-OPDs and to elucidate how this asymmetry governs the device's circular-polarization selectivity. Our focus is therefore on how intentionally created traps modulate CP-dependent photocurrent extraction and enable substantial amplification of g_{ph} without increasing g_{abs}.

We also emphasize that the trap effect itself is not an isolated effect for improving g_{Ph} in our case; such as a simple reduction in charge-carrier mobility caused by trapping in the active layer. Instead, it is a coupled phenomenon arising from the interplay between charge trapping and the polarization-dependent optical penetration depth of CP-OPDs. Specifically, as the optical penetration depth depends on the polarization state of the incident light, the trapping effect becomes highly asymmetric with respect to light polarization when acceptor is introduced, which is a key aspect of our study.

In addition, the PPPc experiment employed in this study is a powerful and well-established technique for identifying trap dynamics in both organic semiconductors and perovskite materials (ref: *Adv. Funct. Mater.* 2025, e18210; *Nat. Commun.* 2023, 14, 8000). Recent studies have demonstrated that PPPc measurements are particularly effective for probing traps formed by the introduction of very small amounts of acceptors into a host semiconductor (ref: *Adv. Funct. Mater.* 2025, e18210). In such systems, where acceptors act as isolated trap sites rather than forming continuous transport pathways, PPPc analysis has been successfully used to directly reveal a trapping effect. These prior demonstrations confirm that PPPc is a validated and sensitive method for investigating trap dynamics induced by low-loading acceptors in material systems closely related to our work, thereby strongly supporting the validity of our analytic tool to study trap dynamics.

In this study, we systematically and qualitatively analyzed asymmetric trapping and the resulting charge extraction characteristics using PPPc and TPC measurements. By examining pristine devices and acceptor-introduced CP-OPDs side by side, we were able to clearly identify how CP-OPDs behave asymmetrically by intentionally introduced traps, which was the robust way to prove our proposed mechanism. Within the scope and aims of this study, such qualitative and comparative analyses provide compelling evidence for the proposed mechanism. Thus, we would like to

emphasize again that a detailed quantitative extraction of all trap-related parameters is therefore beyond our intended focus.

In addition to our answer above, although not directly requested by the reviewer, we provide a simplified conceptual illustration in Fig. R2 and supplementary discussion to further clarify the proposed mechanism and aid further review.

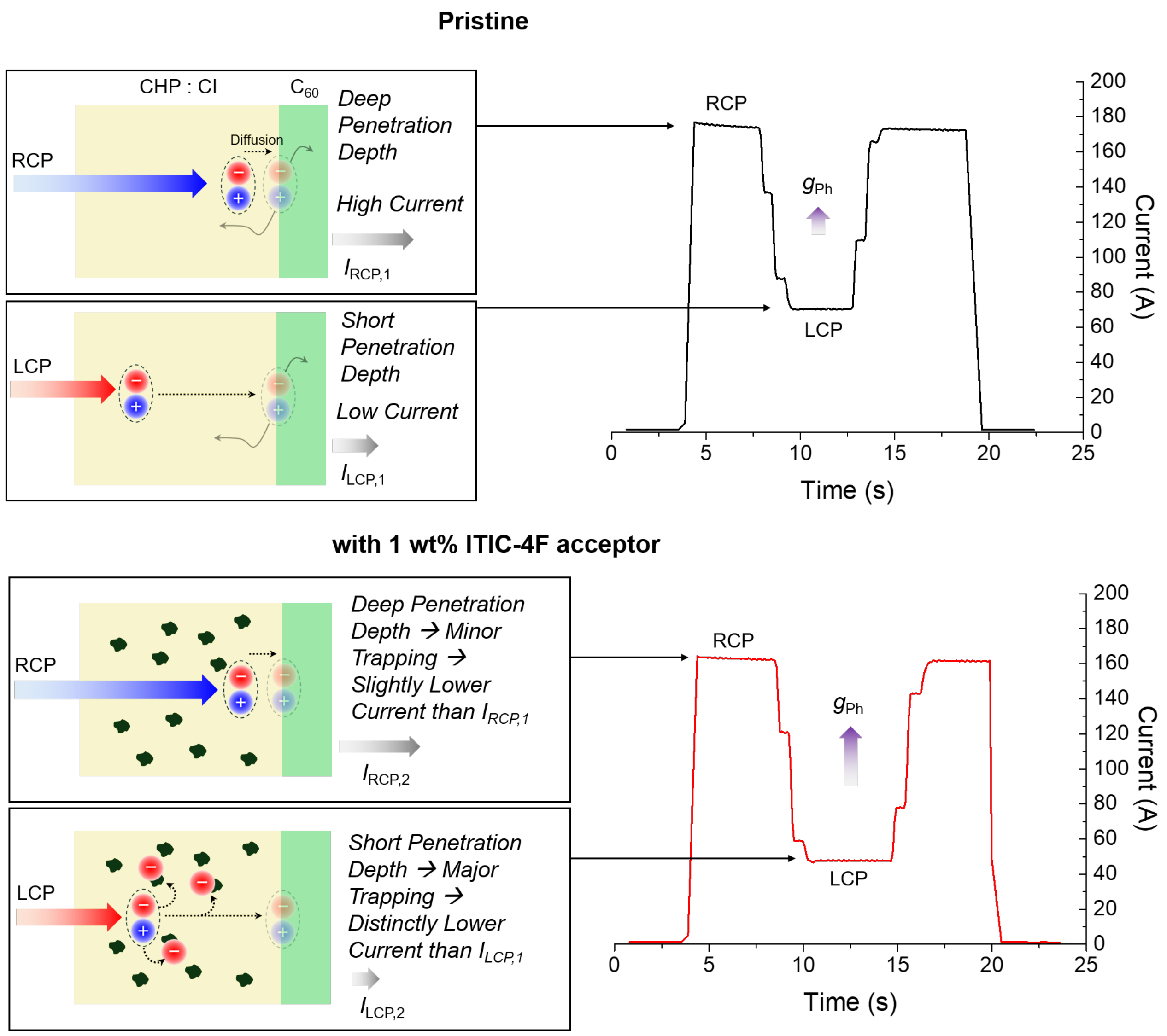

Fig. R2 Simplified schematic illustration of amplified asymmetric trapping in CP-OPDs.

To further support our mechanism, we additionally measured the wavelength-dependent EQE and responsivity and extracted the detectivity of pristine CP-OPDs as well as devices incorporating the opposite enantiomer of the chiral inducer, [*M*]-aza[6]H (Fig. R3). By comparing pristine devices, acceptor-introduced devices, and devices employing the opposite enantiomer (Fig. 1j and Supplementary Figs. 2 and 7 in the revised SI), we confirmed that the acceptor introduction selectively and more strongly influences the device response under one specific polarization state, while having a weaker effect under the opposite polarization state at 470 nm (Table R1). These systematic comparisons in both enantiomeric systems provide strong and consistent evidence supporting the proposed asymmetric-trapping mechanism illustrated in Fig. R2.

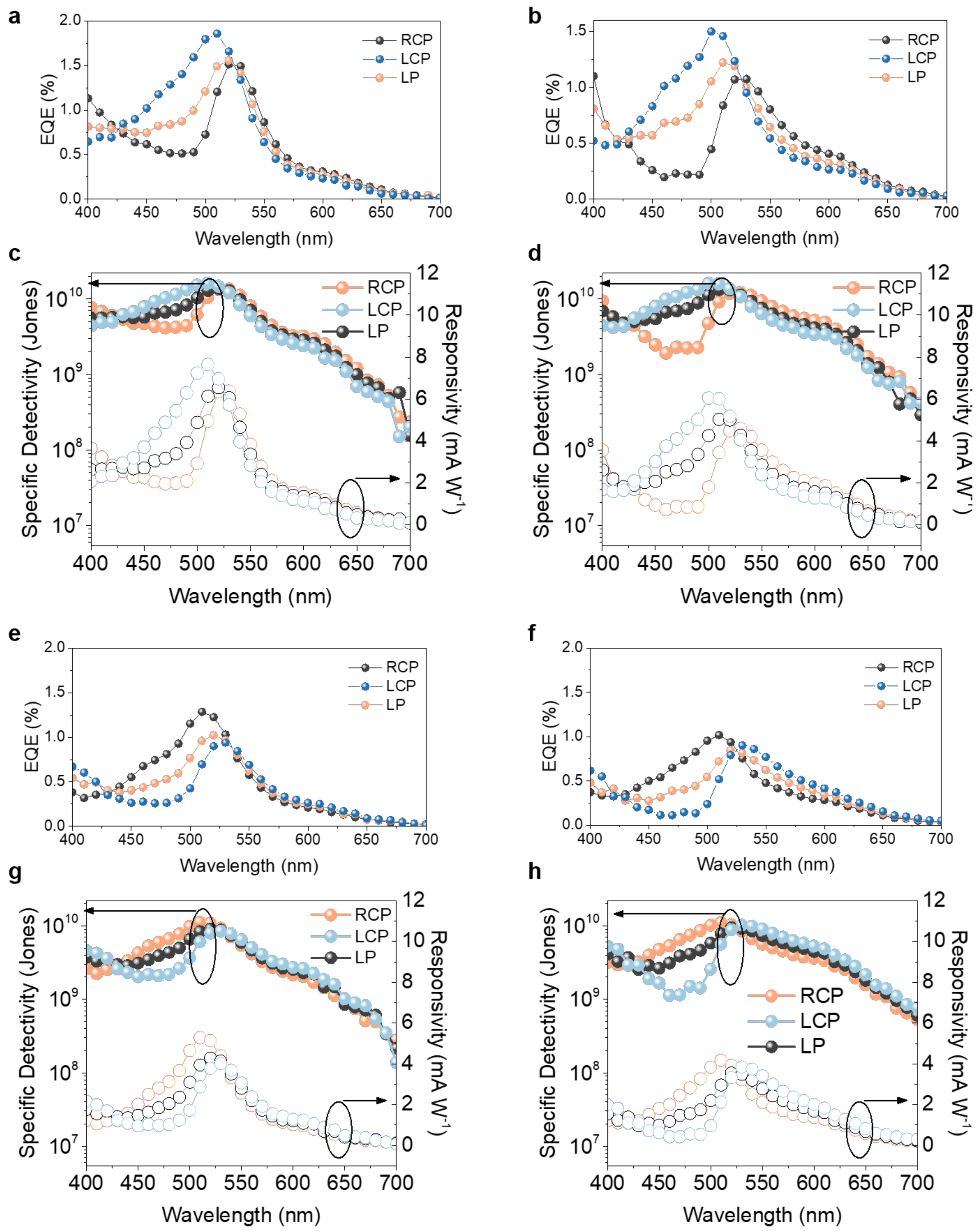

Fig. R3 EQE, *R*, and *D of CP-OPDs by using CHP:CI ([*M*]-aza[6]H) or CHP:CI ([*P*]-aza[6]H) matrices, without acceptor or with ITIC-4F. a–d,** Spectral EQE, *R*, and *D** of pristine CP-OPDs (**a,c**) or ITIC-4F-introduced CP-OPDs (**b,d**) by using CHP:CI ([*M*]-aza[6]H). **e–h,** Spectral EQE, *R*, and *D** of pristine CP-OPDs (**e,g**) or ITIC-4F-introduced CP-OPDs (**f,h**) by using CHP:CI ([*P*]-aza[6]H) under the linear polarized (LP), RCP, and LCP excitation.

CHP:[*P*]-aza[6]H	EQE		*R*		D^*	
	RCP	**LCP**	RCP	**LCP**	RCP	**LCP**
Pristine	0.74	**0.26**	2.82×10^{-3}	$\mathbf{9.86\times10^{-4}}$	5.98×10^{9}	$\mathbf{2.09\times10^{9}}$
ITIC-4F	0.65	**0.11**	2.46×10^{-3}	$\mathbf{4.32\times10^{-4}}$	6.52×10^{9}	$\mathbf{1.15\times10^{9}}$
Change (%)	12.9	**56.2**	12.9	**56.2**	9.0	**45.2**
CHP:[*M*]-aza[6]H	EQE		*R*		D^*	
	**RCP**	LCP	**RCP**	LCP	**RCP**	LCP
Pristine	**0.52**	1.28	$\mathbf{1.96\times10^{-3}}$	4.88×10^{-3}	$\mathbf{4.16\times10^{9}}$	1.04×10^{10}
ITIC-4F	**0.23**	1.08	$\mathbf{8.69\times10^{-4}}$	4.09×10^{-3}	$\mathbf{2.30\times10^{9}}$	1.08×10^{10}
Change (%)	**55.7**	16.2	**55.7**	16.2	**44.6**	4.8

Table. R1 Comparison of EQE, *R*, and *D of CP-OPDs at 470 nm for CHP:CI ([*M*]-aza[6]H) or CHP:CI ([*P*]-aza[6]H) matrices, without acceptor or with ITIC-4F.**

We updated the data and discussion in the manuscript as follows, as follows:

(Page 12 in revised manuscript)

This interpretation is consistent with our device-level observations, including the *I*–*V* curves (Fig. 1f,g), time-dependent real-time CP selectivity (Fig. 1e), and the spectral EQE, *R*, and D^* (Fig. 1j and Supplementary Fig. 7 and 8) as well as results obtained using the opposite enantiomer of CI ([*M*]-aza[6]H) (Supplementary Fig. 2). In both enantiomer cases, introducing the acceptor more strongly influences the device response under one specific polarization state, while having less effect under the opposite polarization state, providing strong support for the proposed mechanism.

Comment 3: Serious concerns arise regarding the reliability of the circular dichroism (CD) measurements and their subsequent analysis. In Figure 2, the absorption spectra of the acceptors are not provided, making it impossible to correlate the observed CD signals with specific electronic transitions of these acceptors. Given the low acceptor loading, their contribution to the overall absorption is negligible—raising the strong possibility that the measured g values for the acceptors are experimental artifacts. Furthermore, comparing the CD spectra in Figure 2a/d, 2b/e, and 2c/f reveals that increasing the acceptor loading from 1% to 5% induces significant changes in CD band shape. These changes show no clear correlation with the acceptors' absorption features (i.e., the associated electronic transitions remain undefined). Collectively, these issues cast substantial doubt on the validity of the CD measurement data and the corresponding analytical interpretations.

Response:

We agree that the CD spectra presented in the previous version did not include the absorbance of the acceptor itself, which may have caused confusion. To address this issue according to the reviewer's comment, we have newly updated both the data presentation and the analysis. As suggested by the reviewer, we first independently measured the absorbance spectra of the acceptors to establish a clear correlation between their optical transitions and the observed CD signals (Fig. R4). We then re-examined the CD spectra of the CHP:Cl films upon incorporation of different acceptors, with particular emphasis on how the acceptors modify the CD features of the CHP:Cl matrix. Because the amount of added acceptor was very small, we focused on spectral changes or the appearance of additional CD peaks induced by the acceptor, rather than on the variation of absolute CD intensity or the g_{abs} values themselves in revised manuscript.

Notably, even at a low loading of 1 wt%, ITIC-4F exhibits a clear induced CD peak near 700 nm (Fig. R4b), which corresponds to the 0–0 transition observed in the absorbance spectrum of an ITIC-4F film (ref: *Mater. Adv.* 2020, 1, 2846–2861). Upon increasing the ITIC-4F content to 5 wt%, a stronger CD peak appears in the same spectral region, indicating strong chiral perturbation of ITIC-4F by the CHP:Cl film.

This induced CD feature at the 0–0 transition is more distinct than that observed for Y6-containing films. When Y6 is introduced at a low loading of 1 wt%, no clearly discernible induced CD peak is observed in the wavelength region corresponding to the 0–0 transition (~850 nm) of Y6 (ref: *J. Phys. Chem. Lett.* 2025, 16, 1367–1375) (Fig. R4a). At a higher loading of 5 wt%, it seemed that a broad CD feature emerged in the spectral region associated with the 0–1 transition (~750 nm), accompanied by the absence of a clearly discernible CD peak associated with the 0–0 transition. Overall, the induced CD features of Y6 remain less distinct than those observed for ITIC-4F at their respective 0–0 transition. For $PC_{71}BM$ (Fig. R4c), no additional discernable CD peaks attributable to its 0–0 transition near 720 nm (ref: *J. Mater. Chem. C* 2014, 2, 3639-3644) or other absorbance region are detected at either 1 wt% or 5 wt% loading, indicating negligible perturbation of $PC_{71}BM$ molecules induced by the CHP:Cl films, which was also confirmed by the absence of CD signals in the $PC_{71}BM$:CI blend film (Supplementary Fig. 10).

We updated the data and interpretation in the revised manuscript as follows:

(Page 7 in revised manuscript) While no changes were observed in the visible region (Fig. 1c), even at a low loading of 1 wt% ITIC-4F, it exhibited a clear induced CD peak near 700 nm (marked with a dashed circle), which corresponds to the 0–0 transition observed in the absorption spectrum of a neat ITIC-4F film (Fig. 2b)[34]. Upon increasing the ITIC-4F content to 5 wt%, a more distinct CD peak appeared in the same spectral region (marked with a dashed circle), suggesting a strong perturbation of ITIC-4F molecules by the CHP:CI film[10,35]. This induced CD feature at the 0–0 transition is more distinct than that observed for Y6-containing films (Fig. 2a). In contrast, when Y6 is introduced at a low loading of 1 wt%, no clearly discernible induced CD peak is observed in the wavelength region corresponding to the 0–0 transition (~850 nm) of Y6[36]. At a higher loading of 5 wt%, a broad CD feature emerged in the spectral region associated with the 0–1 transition (~750 nm), accompanied by the absence of a clearly discernible CD peak associated with the 0–0 transition. Overall, the induced CD features by Y6 remain less distinct than those observed for ITIC-4F at their respective 0-0 transitions. For $PC_{71}BM$, a flat CD profile with no distinct additional CD peak features

attributable to its 0-0 transition near 720 nm[37] or other absorbance region is detected at either 1 wt% or 5 wt% loading (Fig. 2c), indicating that the perturbation of $PC_{71}BM$ molecules by the CHP:Cl films is negligible, which was also confirmed by the absence of CD signals in the $PC_{71}BM$:CI blend film (Supplementary Fig. 10).

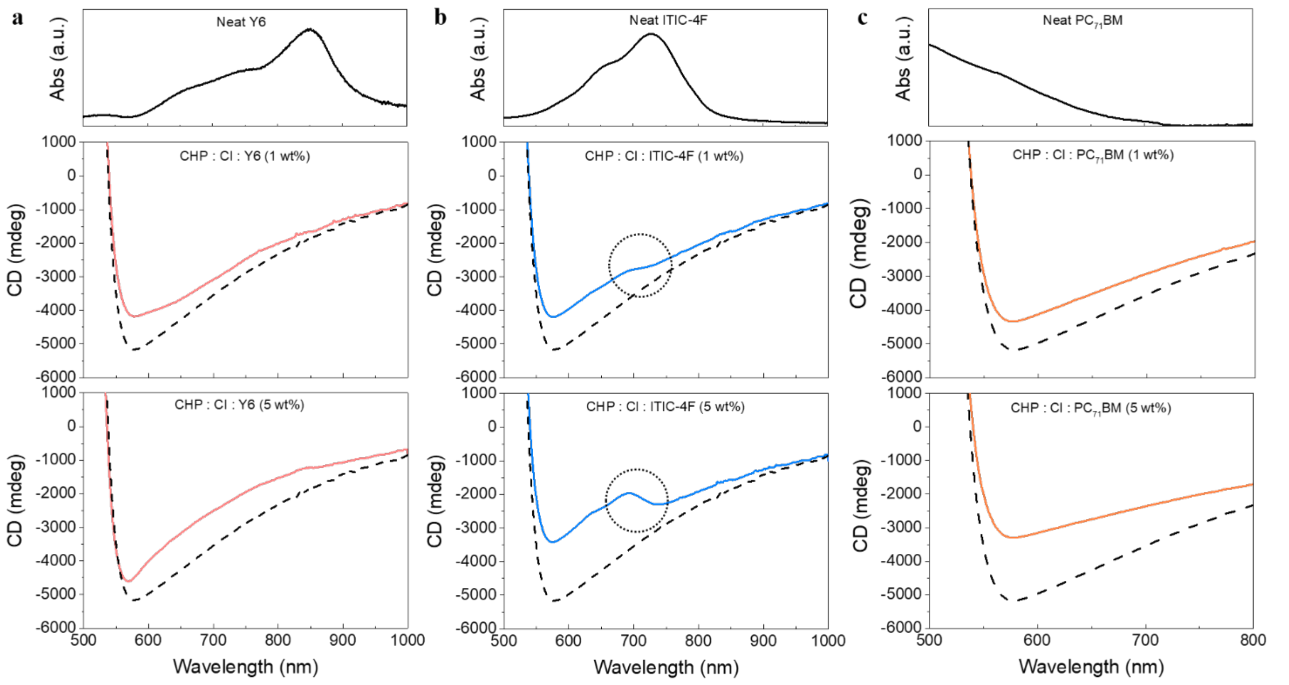

Fig. R4 CD spectra of acceptor introduced CHP:CI films in the region where CHP:CI doesn't absorb. a-c, Absorbance of neat Y6, ITIC-4F, and $PC_{71}BM$ films (top). CD spectra of CHP:CI with a low-content (1%) introduction of Y6, ITIC-4F, and $PC_{71}BM$ (middle). CD spectra of CHP:CI with a high-content (5%) introduction of Y6, ITIC-4F, and $PC_{71}BM$ (bottom). Dashed black line shows the CD spectrum of pristine CHP:CI film.

Comment 4: The mechanism underlying the structure-chirality correlation in the studied systems remains poorly characterized and analyzed. Solution-state diffusion-ordered nuclear magnetic resonance (DOSY) spectroscopy was used to investigate interactions between [P]-aza[6]H (CI) and the acceptors, but this data is insufficient to confirm the miscibility of acceptors with the CHP:CI blend. Notably, the polymer CHP constitutes a major component of the blend, yet the DOSY analysis fails to account for interactions involving CHP—whether with CI or the acceptors.

Response:

Thank you for this important comment. Our thin-film system is intrinsically complex, as it comprises three distinct organic components: the chiral host polymer, the chiral dopant, and the acceptor molecules. In such a multicomponent solid-state system, it is inherently difficult with currently available experimental techniques to unambiguously resolve, in situ, which specific functional groups from each material interact with one another. Direct molecular-level identification of the precise intermolecular interactions and the functional moieties involved would require advanced

spectroscopic or structural probes beyond the scope of the present work and remains an important topic for future investigation.

To gain initial insight into these interactions, we simplified the system to a binary mixture comprising the chiral dopant and the acceptor and employed DOSY NMR analysis to probe their interactions in solution. While molecular interactions in solution cannot be directly equated to those in solid-state films, the DOSY diffusion data provide indirect evidence that the interaction strength with [*P*]-aza[6]H seems to follow the trend ITIC-4F > Y6 > $PC_{71}BM$ in our system. This trend is further supported by circular dichroism (CD) measurements, where the addition of ITIC-4F induced the most pronounced changes.

Nevertheless, we fully agree with the reviewer that our initial analysis did not explicitly include all three components and was therefore insufficient to provide a comprehensive picture of the overall miscibility in our system. To address this limitation, we conducted additional experiments aimed at evaluating miscibility in a general manner. Specifically, we performed contact angle measurements to extract surface free energies (γ) and estimate miscibility (χ) using the Flory–Huggins framework. Within the Flory−Huggins model, the interaction parameter χ serves as a thermodynamic indicator of miscibility by quantifying the energetic penalty associated with mixing components. A smaller χ value corresponds to more favorable intermolecular interactions and thus higher miscibility, whereas a larger χ value indicates unfavorable interactions that promote phase separation. Therefore, comparing χ values among different material combinations provides a quantitative and comparative measure of their relative miscibility (ref: *Chem. Mater.* 2025, 37, 4, 1541–1553).

Note that surface energy extraction based on contact angle measurements was performed under conditions that closely reflect the actual thin-film environment in the devices. In our system, the active layer is predominantly composed of the host polymer F8T2, together with 10 wt% [*P*]-aza[6]H and a significantly smaller fraction (1 wt%) of the acceptor. Accordingly, contact angle measurements were carried out on neat F8T2 films and on [*P*]-aza[6]H:acceptor blend films, since F8T2 and [*P*]-aza[6]H constitute the major components of the thin film. In addition, [*P*]-aza[6]H:acceptor blend films were prepared with an acceptor to [*P*]-aza[6]H ratio of 1:10, identical to that used in the devices. This approach reflects the relatively minor acceptor content compared to both F8T2 and [*P*]-aza[6]H in the active layer. In addition, we also performed contact angle measurements by using a F8T2:[*P*]-aza[6]H film and each acceptor film.

As shown in Supplementary Fig. 12 and 13 in revised SI, the surface free energies were calculated from contact angle measurements using water, ethylene glycol (EG), and diiodomethane (DIM) as probe liquids, based on the Owens–Wendt–Rabel–Kaelble (OWRK) method (ref: *J. Appl. Polym. Sci.* 1969, 13, 1741). Based on these values, Flory–Huggins interaction parameters were subsequently calculated to assess the miscibility between materials. The extracted Flory–Huggins interaction parameters follow the order [*P*]-aza[6]H:ITIC-4F (or ITIC-4F) < [*P*]-aza[6]H:Y6 (or Y6) < [*P*]-aza[6]H:$PC_{71}BM$ (or $PC_{71}BM$), indicating that the ITIC-4F-containing [*P*]-aza[6]H (or ITIC-4F) exhibits the strongest miscibility with the F8T2 host polymer (or F8T2:[*P*]-aza[6]H) among the investigated acceptors. This result provides further information on the miscibility behavior containing all three components.

We added the following further discussion and experimental data to the revised manuscript and revised SI as follows:

(Page 9 in revised manuscript) To elucidate the miscibility trend of the multicomponent system, contact angle measurements were performed to extract surface free energies (γ) and estimate miscibility using the Flory–Huggins framework. Surface free energies were calculated from contact angles measured with water, ethylene glycol (EG), and diiodomethane (DIM) as probe liquids (Supplementary Fig. 12 and Fig. 13), and the corresponding Flory–Huggins interaction parameters (χ) were derived to assess the miscibility between the F8T2 host polymer (or F8T2:[*P*]-aza[6]H) and the [*P*]-aza[6]H:acceptor (or acceptor) systems. The extracted Flory–Huggins interaction parameters follow the order [*P*]-aza[6]H:ITIC-4F (or ITIC-4F) < [*P*]-aza[6]H:Y6 (or Y6) < [*P*]-aza[6]H:$PC_{71}BM$ (or $PC_{71}BM$), indicating that the [*P*]-aza[6]H:ITIC-4F (or ITIC-4F) exhibits the strongest miscibility with the F8T2 (or F8T2:[*P*]-aza[6]H) among the investigated acceptors. This trend provides information on the general miscibility behavior between the host F8T2 polymer (or F8T2:[*P*]-aza[6]H) and the [*P*]-aza[6]H:acceptor (or acceptor) system containing all three components.

(Page S13 in revised SI)

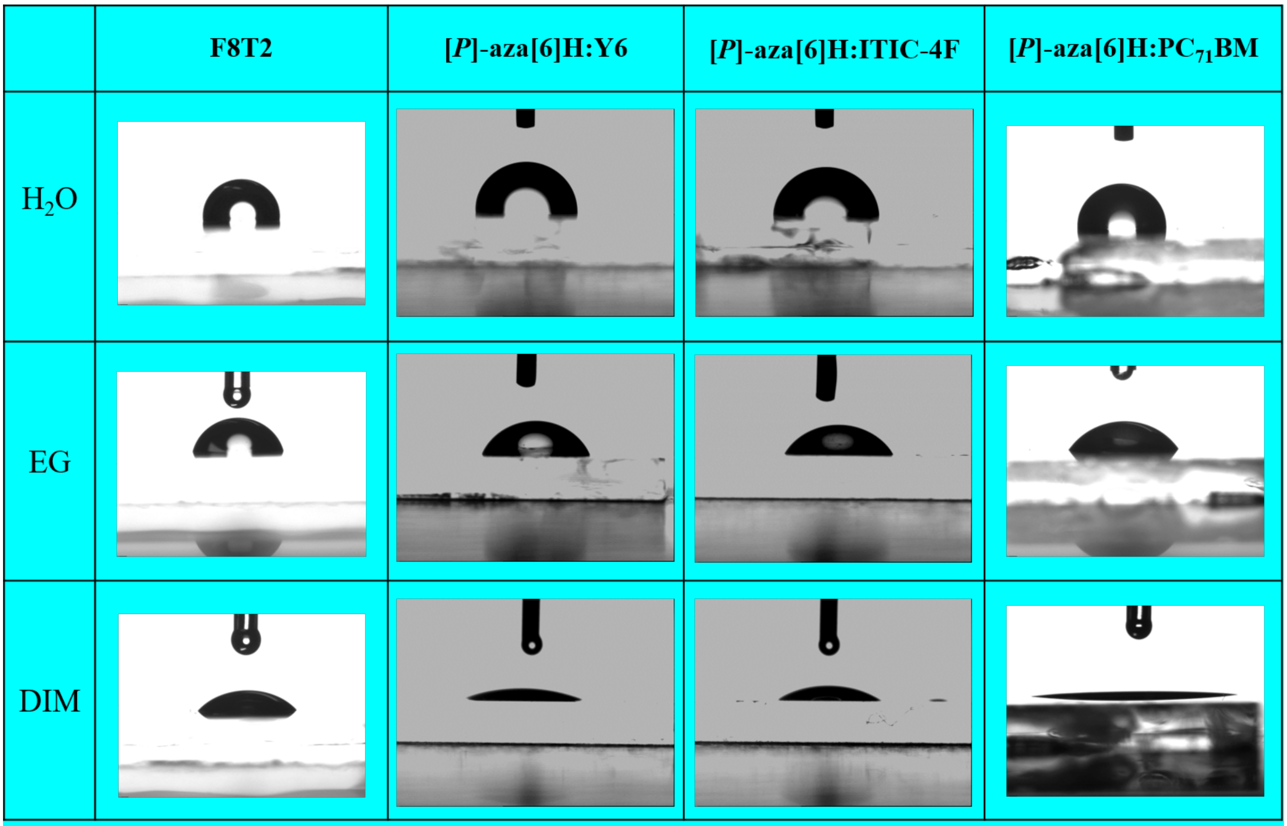

	θ_{water} (°)	θ_{EG} (°)	θ_{DIM} (°)	γ (mM/m)	χ
F8T2	100.8	75.2	48.8	31.5	
[*P*]-aza[6]H:Y6	96.3	66.4	27.5	40.7	0.58
[*P*]-aza[6]H:ITIC-4F	93.4	60.6	34.5	39.5	0.45
[*P*]-aza[6]H:$PC_{71}BM$	94.2	57.9	4.6	46.7	1.49

Supplementary Figure 12| Contact angle measurements. The surface energy was calculated by using Owens–Wendt–Rabel–Kaelble (OWRK) method[1]. The Flory-Huggins interaction parameter between F8T2 and [*P*]-aza[6]H:acceptor was calculated by using $\chi = (\sqrt{\gamma_{F8T2}} - \sqrt{\gamma_{aza[6]H:acceptor}})^2$, where γ_{F8T2} and $\gamma_{aza[6]H:accpetor}$ are surface energies of F8T2 and [*P*]-aza[6]H:acceptor blend. Surface energy extraction based on contact angle measurements was performed under conditions that closely reflect the actual thin-film environment in the devices. In our system, the active layer is predominantly composed of the host polymer F8T2, together with 10 wt% [*P*]-aza[6]H and a significantly smaller fraction (1 wt%) of the acceptor. Accordingly, contact angle measurements were carried out on neat F8T2 films and on [*P*]-aza[6]H:acceptor blend films, since F8T2 and [*P*]-aza[6]H constitute the major components of the thin film. In addition, [*P*]-aza[6]H:acceptor blend films blend films were prepared with an acceptor to [*P*]-aza[6]H ratio of 1:10, identical to that used in the devices.

(Page S14 in revised SI)

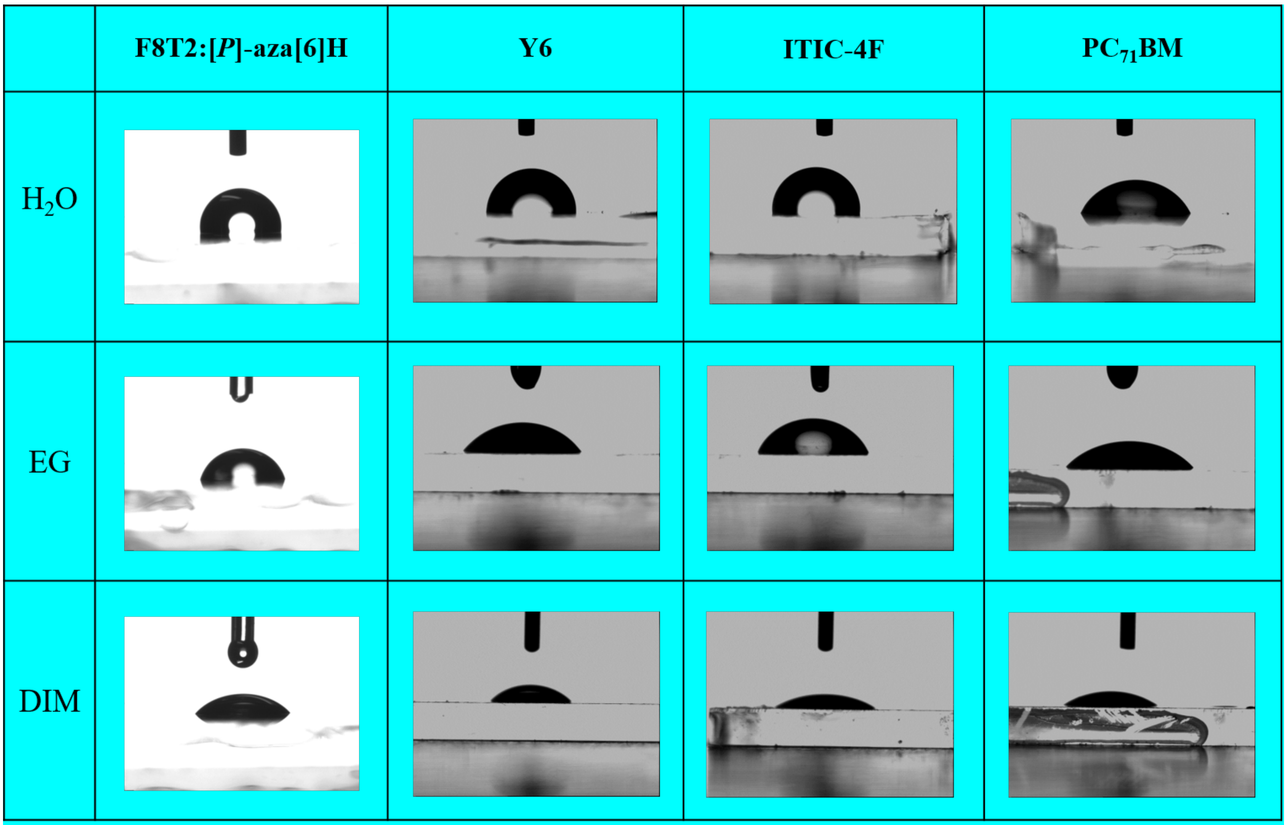

	θ_{water} (°)	θ_{EG} (°)	θ_{DIM} (°)	γ (mM/m)	χ
F8T2:[*P*]-aza[6]H	99.8	75.6	49.6	30.9	
Y6	92.9	53.3	46.0	37.1	0.28
ITIC-4F	99.1	68.2	45.3	35.7	0.17
$PC_{71}BM$	65.4	48.3	33.8	44.0	1.15

Supplementary Figure 13| Contact angle measurements. The surface energy was calculated by using Owens–Wendt–Rabel–Kaelble (OWRK) method[1]. The Flory-Huggins interaction parameter between F8T2 and [*P*]-aza[6]H:acceptor was calculated by using $\chi = (\sqrt{\gamma_{\mathrm{F8T2:}[P]\text{-aza}[6]\mathrm{H}}} - \sqrt{\gamma_{\mathrm{acceptor}}})^2$, where $\gamma_{\mathrm{F8T2:}[P]\text{-aza}[6]\mathrm{H}}$ and $\gamma_{\mathrm{accpetor}}$ are surface energies of F8T2:[*P*]-aza[6]H blend and acceptor. To ensure relevance to device conditions, films were processed using toluene, the same solvent employed during device fabrication.

Comment 5: The highest gPh is obtained under reverse bias. The reverse bias may induce the artifacts when calculating the gPh. The intrinsic gph without bias needs to be compared and analyzed.

Response:

As shown in Fig. 1i in the manuscript, under zero bias conditions, the absolute photocurrent dissymmetry factor ($|g_{\mathrm{Ph}}|$) values are 0.51 and 0.66 for the pristine and ITIC-4F–incorporated CP-OPDs, respectively. Notably, the enhancement in $|g_{\mathrm{Ph}}|$ becomes much more pronounced upon application of reverse bias. This indicates that acceptor incorporation has only a limited impact on dissymmetric photoresponse at zero bias.

We attribute this behavior to intrinsically restricted exciton dissociation and charge extraction under zero bias, arising from our device configuration and the relatively thick chiral active layer. Specifically, our devices employ a bilayer architecture consisting of a thick chiral active layer and a C_{60} layer, rather than a bulk heterojunction structure with a thin active layer. In our device configuration, the built-in electric field is primarily confined to the interfacial region between the active layer and the C_{60} layer, which likely limits exciton dissociation and charge extraction throughout the bulk of the active layer under zero bias conditions. When no external voltage is applied, electron transport is substantially suppressed, leading to reduced carrier flux, thereby a lower probability of electrons encountering trap sites. Consequently, trapping events become less frequent and do not significantly influence the asymmetric trapping effect in CP-OPDs, leading to a much reduced impact of acceptor addition on CP selectivity.

Our intrinsic device behavior is consistent with the previous report of a chiral polmer donor with a bilayer device structure, but without addition of traps (*Adv. Opt. Mater*. 2022, 10, 2101044). In this prior study $|g_{\mathrm{Ph}}|$ was also significantly enhanced upon application of reverse bias, with the use of a thick active layer and bilayer device structure. Accordingly, even though acceptor incorporation can induce asymmetric trapping effects, its influence on $|g_{\mathrm{Ph}}|$ remains weak at zero bias because exciton dissociation and charge extraction are fundamentally limited in this device structure under this condition.

We added further discussion to the revised manuscript as follows:

(Page 13 in revised manuscript) Meanwhile, the enhancement of g_{Ph} was minor under zero-bias conditions (Fig. 1i). This behavior can be attributed to limited exciton dissociation and charge extraction under zero bias, arising from the bilayer device architecture consisting of a thick chiral

active layer and a C_{60} layer. In this configuration, the built-in electric field is mainly localized at the active layer/C_{60} interface, thereby restricting exciton dissociation and carrier extraction within the bulk of the active layer. As a result, carrier transport is expected to be strongly suppressed under zero bias, reducing the carrier flux and the probability of electrons encountering trap sites. Consequently, trapping events may occur less frequently, weakening the asymmetric trapping effect and leading to a much reduced impact of acceptor addition on CP selectivity.

Furthermore, we do not attribute the observed trend in g_{Ph} to measurement artifacts, as the same trend is consistently observed not only in *I–V* measurements but also in real-time CP light detection experiments (Fig. R5 and Fig. 1e). In the real-time CP detection measurements, all devices were measured in current–time mode under the same fixed bias voltage, while the incident polarization was sequentially switched between right-handed circular polarization (RCP) and left-handed circular polarization (LCP) in real time. The reproducible and polarization-dependent photocurrent responses confirm that the extracted g_{Ph} values are intrinsic to the devices and are not influenced by experimental artifacts. Because the polarization state is alternated continuously and the photocurrent is recorded simultaneously in real time, any instrumental noise or systematic error would affect signal recording equally, and thus cannot produce the consistent asymmetry observed. This ensures that the measured g_{Ph} reflects the true chiral response of the device rather than artifacts of the measurement system.

In addition, to further prove the observation from *I–V* curves and proposed mechanism for improved g_{Ph}, we newly measured wavelength dependent EQE and *R*, and extracted D^* of CP-OPDs incorporating [*M*]-aza[6]H without or with the introduction of ITIC-4F by using F8T2:[*M*]-aza[6]H blends at the reverse bias of 1 V. By comparing all EQE, *R*, and D^* in both enantiomer cases without or with ITIC-4F as shown in Fig. R3, it was confirmed that the introduction of the ITIC-4F impacted more strongly EQE, *R*, and D^* under one specific polarization, with a much smaller effect under the opposite polarization at 470 nm, which is consistent what we observed from *I–V* curves and real-time *I–t* curves, and proposed mechanism displayed in Fig. R2. This effect resulted in a substantially enhanced polarization-dependent differential response. The newly obtained EQE, *R*, and D^* data, directly comparing pristine and ITIC-4F-introduced CP-OPDs, validate our central argument and proposed mechanism.

Collectively, a various of results obtained from *I–V* curves, real-time *I–t*, spectral EQE, responsivity, and detectivity provide a consistent result and strong comparative evidence, confirming that the observed trend in g_{ph} do not arise from artefacts.

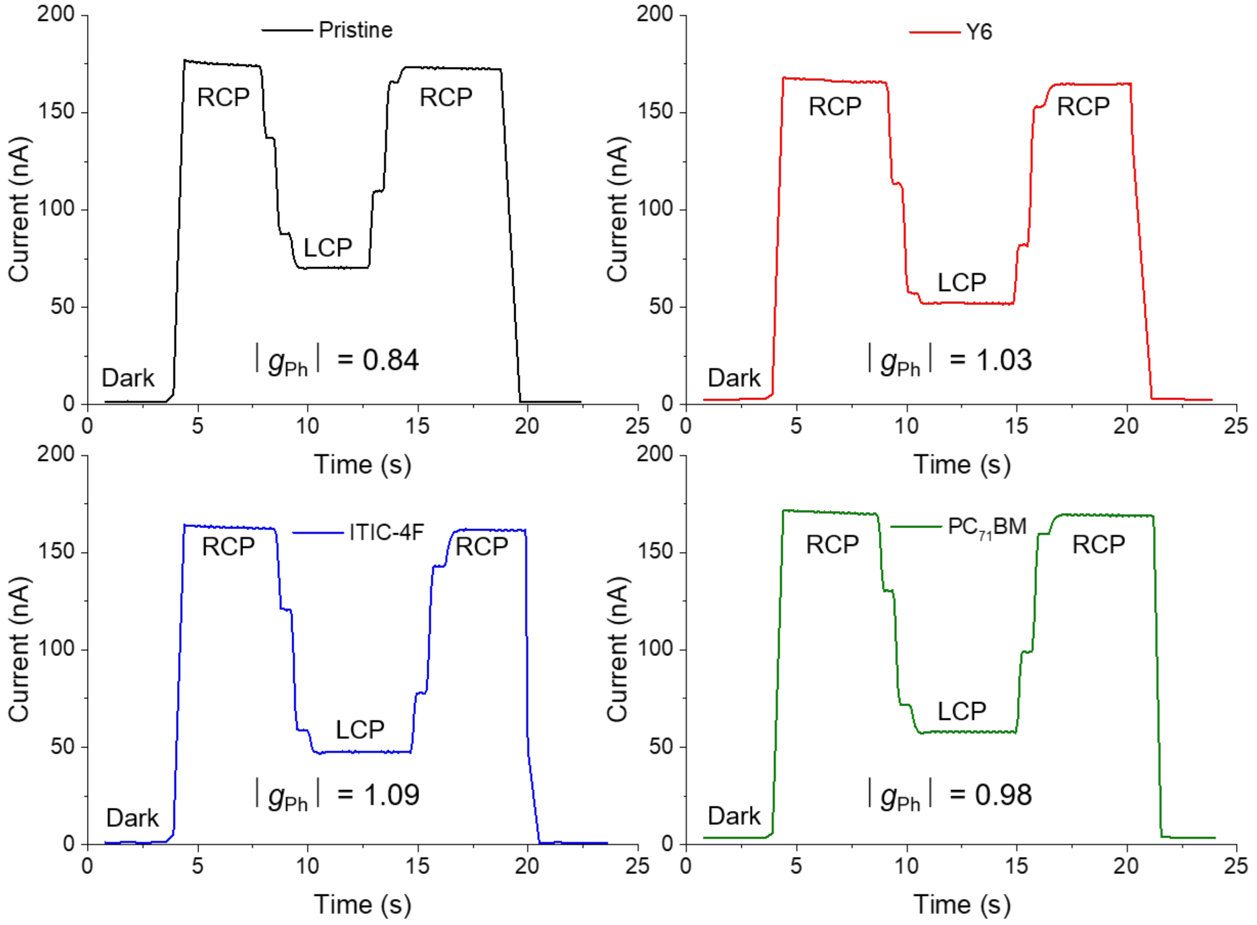

Fig. R5 Real-time CP selectivity, where all devices (F8T2:[*P*]-aza[6]H without or with acceptors) were measured at the same fixed bias voltage (1 V) in current–time mode, while the incident polarization was alternated sequentially between RCP and LCP.

Reviewer #2

In this manuscript, the authors explore the fundamental question of "potential mechanisms for asymmetric enhancement." Through detailed investigations in materials science and advanced spectroscopy, they reveal the crucial role of asymmetric trap dynamics in amplifying the gph within CP OPDs. This approach enabled the realization of a large area organic circularly polarized imaging system, demonstrating its potential for real time spatiotemporal visualization of optical chirality and CNC assisted pattern encryption. However, certain vague descriptions—particularly the lack of clear core arguments—limit the paper's practical applicability. The following questions and recommendations should be addressed:

Response:

Thank you for your constructive comments. We are deeply grateful to the reviewer for the constructive and thoughtful comments, which were very helpful for improving our manuscript.

Comment 1: On page 4, the authors state: "We bridge this knowledge gap by incorporating a selection of acceptors into the chiral host polymer (CHP):CI blend and investigating CP OPD performance." Please elaborate on the rationale for selecting these specific acceptors and the CHP:CI blend. Why is

this system expected to "bridge this knowledge gap"? Relying solely on prior team results to justify the choice appears too vague.

Response:

Thank you for your valuable comment. The rationale for selecting the acceptor materials was explained in the main text as follows: "we selected acceptors of distinct materials classes: Y6 and ITIC-4F (planar non-fullerene acceptors, NFAs), and $PC_{71}BM$ (a non-planar fullerene derivative) (Fig. 1b). The planar NFAs are more susceptible to conformational perturbations induced by CIs, which can drive them into chiral configurations, as reported previously[10]. In contrast, the non-planar fullerene derivative possesses greater structural rigidity and are therefore less responsive to such perturbations. These structural distinctions are expected to affect the homogeneity of the acceptors within the chiral blend, leading to variations in the interfacial area within the chiral material. Such variations may, in turn, influence the overall charge dynamics of CP-OPDs."

In addition, F8T2 was chosen as the chiral matrix because this polymer is readily commercially available and fluorene-based copolymer is well known to exhibit strong chirality when blended with a chiral inducer (aza[6]H). Owing to this characteristic, F8T2 has been widely employed in chiral optoelectronic materials research (ref: *Nat. Commun.* 2020, 11, 6137; *Adv. Optical Mater.* 2022, 10, 2101044; *Nature* 2023, 617, 92–99). The rationale for selecting the F8T2 polymer was also explained in the main text as follows: "This blend was chosen because our previous work demonstrated that thin films prepared with this ratio exhibited a high g_{abs} of 0.7 and excellent film homogeneity, enabling planar CP-OPDs with absolute g_{Ph} values approaching 0.8[19]"

However, as pointed out by the reviewer, our study primarily focuses on the optimization and utilization of a single polymer-based chiral matrix. Therefore, we acknowledge that the statement "We bridge this knowledge gap by incorporating a selection of acceptors into the chiral host polymer (CHP):CI blend and investigating CP OPD performance." may not be universally applicable to all chiral material systems and could be interpreted ambiguously.

Prior studies have insufficiently addressed the role of dissymmetric charge carrier extraction process in CP-OPDs. While material chirality and optical dissymmetry have been extensively studied, the contribution of charge carrier dynamics particularly in chiral optoelectronic systems remains poorly understood. Importantly, chare carrier extraction process in CP-OPDs can differ fundamentally from those in conventional non-polarized photodetectors, considering the optical penetration depth basically varies depending on the polarization state, yet the impact of these differences on device's dissymmetric factor, coupled with the introduction of an acceptor, has not been investigated. In this regard, our intent was not to suggest a generalizable material strategy, but rather to highlight a conceptual gap in the current understanding of charge transfer processes that may influence CP photodetector selectivity.

But still acknowledging the ambiguity identified by the reviewer, we have revised the Introduction part to more clearly justify our material selection and to explicitly articulate the originality of this study. Rather than presenting a broadly applicable material integration strategy, we now emphasize the deliberate modulation of dissymmetric behavior in chiral optoelectronic devices through the introduction of acceptors, highlighting the novelty of our work: demonstrating how acceptor-assisted

trapping effect and thereby asymmetric trap dynamics can be used as an effective strategy to control CP selectivity, an aspect that has been never explored in previous studies.

(Page 4 in revised manuscript)

Considering charge-carrier extraction process in CP-OPDs can differ fundamentally from those in conventional non-polarized photodetectors due to the variable optical penetration depth depending on the polarization state, yet the impact of these differences on g_{Ph}, coupled with the introduction of an acceptor, has not been investigated. In this work, by incorporating a small amount (1 wt%) of non-fullerene small molecule acceptors (NFAs) into a chiral host material, we intentionally create trap states that asymmetrically affect carrier extraction based on the handedness of incident CP light. This trap-induced asymmetry significantly enhances g_{Ph} , uncovering a previously untapped mechanism for performance improvement.

Comment 2: It's reported that adding an acceptor to the [P] aza[6]H system weakens the device's circularly polarized photocurrent response, with a significantly larger effect on LCP than on RCP (Figure 1e). However, when [P] aza[6]H is replaced with [M] aza[6]H, the acceptor doped devices show varied trends in RCP and LCP response depending on the acceptor type, without a clearly dominant polarization effect (Supplementary Figs 1 and 2). This observation seems inconsistent with the authors' core argument and requires explanation.

Response:

In the case of g_{Ph}, the comparison of trends is reliable. However, the absolute current values may show slight variations from batch to batch or between different measurements. Such variations are most likely attributable to minor concentration fluctuations during solution preparation and other subtle process-related factors, which can lead to small variations in film thickness or acceptor concentration.

To further elucidate the trend of CP-OPDs with the opposite enantiomer (also in response to comment 6 below), we updated the manuscript using newly acquired data and applied the same analysis as that used in the main figures to CP-OPDs based on [*M*]-aza[6]H. We confirmed that g_{Ph} trend follows the same trend as CP-OPDs with [*P*]-aza[6]H, and I–V curves and time-dependent detection characteristics under CP light revealed that the introduction of the acceptor impacts I_{RCP}, with a much smaller effect on I_{LCP}. This effect maximized the difference between I_{LCP} and I_{RCP}, amplifying g_{Ph}. This behavior is completely reversed compared to CP-OPDs containing [*P*]-aza[6]H, confirming that the observed trend is similarly present in CP-OPDs incorporating [*M*]-aza[6]H, but with opposite handedness.

In addition, to further prove the observation from the I–V curves and proposed mechanism for improved g_{Ph}, we newly measured wavelength dependent EQE and R, and extracted D^* of CP-OPDs incorporating [*M*]-aza[6]H without or with the introduction of ITIC-4F (as already discussed in our response to reviewer 1). As shown in Supplementary Fig. 2 in revised SI, all EQE, R, and D^* data showed that the introduction of the acceptor impacted more strongly EQE, R, and D^* under RCP illumination, with a much smaller effect under LCP illumination. This effect resulted in a substantially enhanced polarization-dependent differential response. Our newly obtained EQE, R, and D^* data comparing between pristine and ITIC-4F-introduced CP-OPDs verifies our core argument and

mechanism, which is consistent with the results with the case using [*P*]-aza[6]H. As a result, our new data provides strong comparative evidence.

Our device-level observations include the *I*–*V* curves (Fig. 1f,g), time-dependent real-time CP selectivity (Fig. 1e), and the spectral EQE, *R*, and D^* (Fig. 1j and Supplementary Fig. 7 and 8) as well as results obtained using the opposite enantiomer of CI ([*M*]-aza[6]H) (Supplementary Fig. 2). In both enantiomer cases, introducing the acceptor more strongly influences the device response under one specific polarization state (Table R1), while having less effect under the opposite polarization state, providing strong support for the proposed mechanism.

To further strengthen the core observation and present consistent behaviour of CP-OPDs with an opposite enantiomer, we updated the manuscript, as follows:

(Page 12 in revised manuscript)

This interpretation is consistent with our device-level observations, including the *I*–*V* curves (Fig. 1f,g), time-dependent real-time CP selectivity (Fig. 1e), and the spectral EQE, *R*, and D^* (Fig. 1j and Supplementary Fig. 7 and 8) as well as results obtained using the opposite enantiomer of CI ([*M*]-aza[6]H) (Supplementary Fig. 2). In both enantiomer cases, introducing the acceptor more strongly influences the device response under one specific polarization state, while having less effect under the opposite polarization state, providing strong support for the proposed mechanism.

(Page S3 in revised SI)

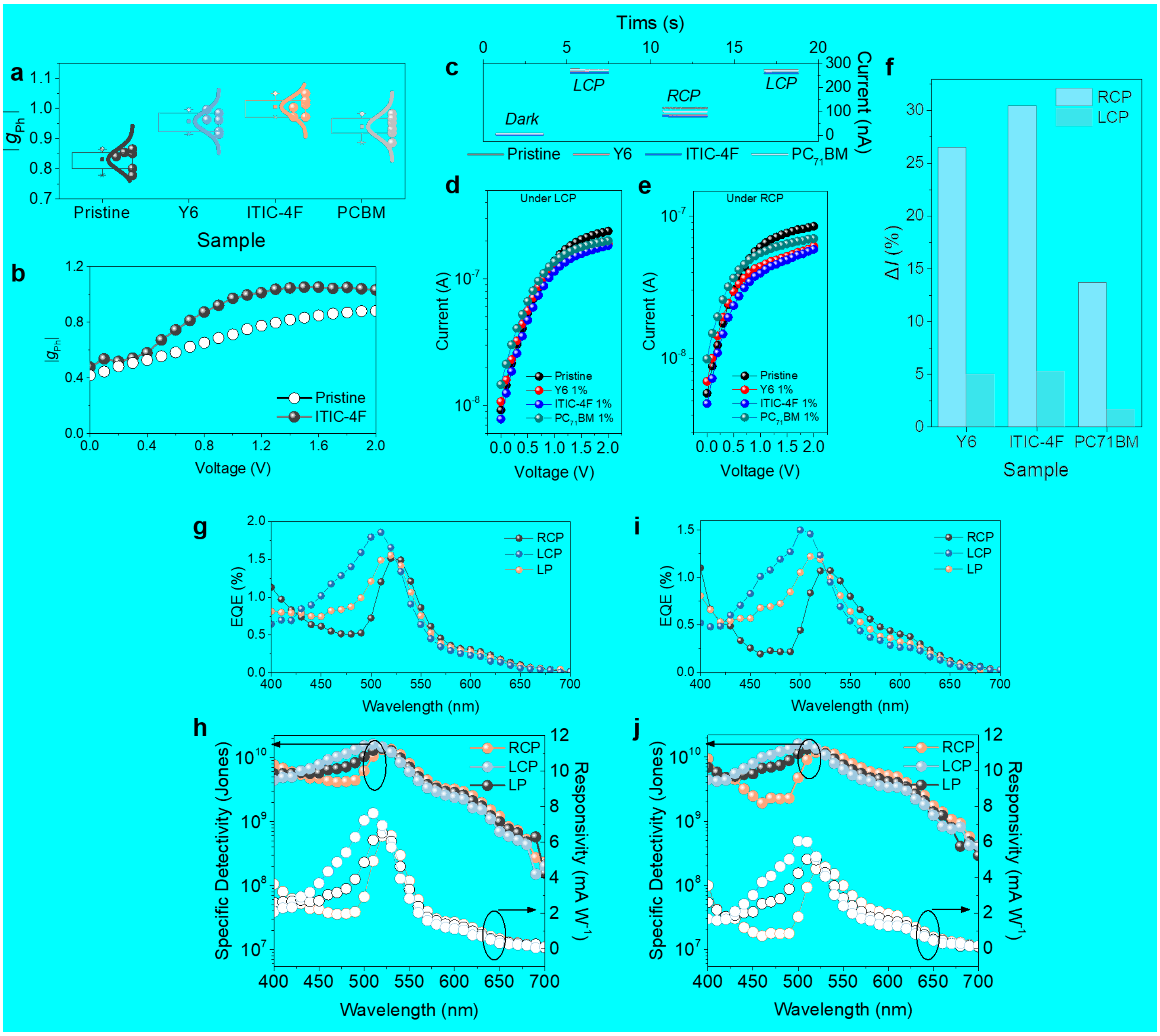

Supplementary Figure 2| g_{Ph} trends and device characteristics of CP-OPDs by using CHP:CI ([*M*]-aza[6]H) matrices, without acceptor or with acceptors. a, Statistics of g_{Ph} for each type of CP-OPDs under 470 nm CP light (23.6 mW cm^{-2}). **b**, Voltage-dependent g_{Ph} for the pristine and the ITIC-4F-incorporated CP-OPD. **c,** Time-dependent CP light detection characteristics for each type of device. **d,e,** *I*–*V* curves of each CP-OPDs under LCP (**d**) and RCP illumination (**e**). **f,** Photocurrent difference under RCP and LCP compared to those from the pristine device. **g–j,** EQE, Spectral *R*, and D^* of pristine CP-OPDs (**g,h**) or ITIC-4F-introduced CP-OPDs (**i,j**) under the linear polarized (LP), RCP, and LCP excitation.

CHP:[*P*]-aza[6]H	EQE		*R*		D^*	
	RCP	**LCP**	RCP	**LCP**	RCP	**LCP**
Pristine	0.74	**0.26**	2.82×10^{-3}	$\mathbf{9.86\times10^{-4}}$	5.98×10^{9}	$\mathbf{2.09\times10^{9}}$

ITIC-4F	0.65	**0.11**	2.46×10^{-3}	$\mathbf{4.32\times10^{-4}}$	6.52×10^{9}	$\mathbf{1.15\times10^{9}}$
Change (%)	12.9	**56.2**	12.9	**56.2**	9.0	**45.2**
CHP:[*M*]-aza[6]H	EQE		*R*		*D**	
	**RCP**	LCP	**RCP**	LCP	**RCP**	LCP
Pristine	**0.52**	1.28	$\mathbf{1.96\times10^{-3}}$	4.88×10^{-3}	$\mathbf{4.16\times10^{9}}$	1.04×10^{10}
ITIC-4F	**0.23**	1.08	$\mathbf{8.69\times10^{-4}}$	4.09×10^{-3}	$\mathbf{2.30\times10^{9}}$	1.08×10^{10}
Change (%)	**55.7**	16.2	**55.7**	16.2	**44.6**	4.8

Table. R1 Comparison of EQE, *R*, and *D of CP-OPDs at 470 nm for CHP:CI ([*M*]-aza[6]H) or CHP:CI ([*P*]-aza[6]H) matrices, without acceptor or with ITIC-4F.**

Comment 3: It is recommended to provide CD spectra of the neat CHP:CI film (without acceptor) for comparison. The authors should also discuss in detail how acceptor incorporation affects CD signal intensity and corresponding wavelengths. Furthermore, why do the CD signals for some acceptor films increase (Figures 2a and 2d) while others decrease (Figures 2b, 2e, 2c and 2f) as the acceptor content rises from 1 wt% to 5 wt%?

Response:

As discussed in response to comment 3 of reviewer 1, we have remasured and updated the CD data. As suggested by this reviewer, we also provided the neat CD spectrum of CHP:CI film for the clear comparison. We also added the absorbance of neat Y6, ITIC-4F, and $PC_{71}BM$ film to clearly correlate the absorbance of acceptors with CD features observed in CHP:CI:acceptor films. We then re-examined the CD spectra of the CHP:Cl films upon incorporation of different acceptors, with particular emphasis on how the acceptors modify the CD shape of the CHP:Cl matrix (Fig. R6). Because the amount of acceptor added was very small, our interpretation focused on changes in the CD spectral shape or additional appearance of CD peak induced by acceptor incorporation, rather than on absolute variations in CD intensity or *g* values. Consequently, we revised the discussion on how acceptor incorporation affects CD feature of pristine CHP:CI films, correlated with the absorbance of neat acceptors, as follows:

(Page 7 in revised manuscript) While no changes were observed in the visible region (Fig. 1c), even at a low loading of 1 wt% ITIC-4F, it exhibited a clear induced CD peak near 700 nm (marked with a dashed circle), which corresponds to the 0–0 transition observed in the absorption spectrum of a neat ITIC-4F film (Fig. 2b)[34]. Upon increasing the ITIC-4F content to 5 wt%, a more distinct CD peak appeared in the same spectral region (marked with a dashed circle), suggesting a strong perturbation of ITIC-4F molecules by the CHP:CI film[10,35]. This induced CD feature at the 0–0 transition is more distinct than that observed for Y6-containing films (Fig. 2a). In contrast, when Y6

is introduced at a low loading of 1 wt%, no clearly discernible induced CD peak is observed in the wavelength region corresponding to the 0–0 transition (~850 nm) of Y6[36]. At a higher loading of 5 wt%, a broad CD feature emerged in the spectral region associated with the 0–1 transition (~750 nm), accompanied by the absence of a clearly discernible CD peak associated with the 0–0 transition. Overall, the induced CD features by Y6 remain less distinct than those observed for ITIC-4F at their respective 0-0 transitions. For $PC_{71}BM$, a flat CD profile with no distinct additional CD peak features attributable to its 0-0 transition near 720 nm[37] or other absorbance region is detected at either 1 wt% or 5 wt% loading (Fig. 2c), indicating that the perturbation of $PC_{71}BM$ molecules by the CHP:Cl films is negligible, which was also confirmed by the absence of CD signals in the $PC_{71}BM$:CI blend film (Supplementary Fig. 10).

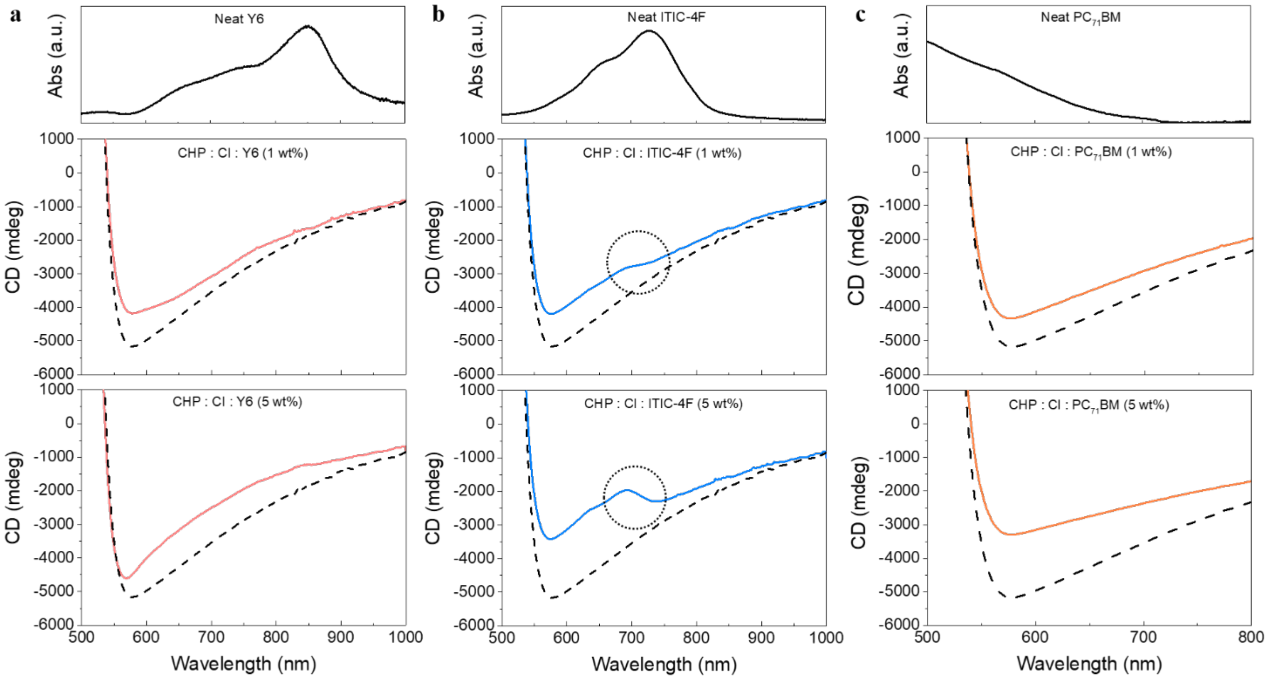

Fig. R6 CD spectra of acceptor introduced CHP:Cl films. a-c, Absorbance of neat Y6, ITIC-4F, and $PC_{71}BM$ films (top). CD spectra of CHP:CI with a low-content (1%) introduction of Y6, ITIC-4F, and $PC_{71}BM$ (middle). CD spectra of CHP:CI with a high-content (5%) introduction of Y6, ITIC-4F, and $PC_{71}BM$ (bottom). Dashed black line shows the CD spectrum of pristine CHP:CI film.

Comment 4: The authors note: "When the acceptor concentration was increased to 5 wt%, ITIC 4F exhibited a more pronounced CD with bisignate Cotton effects, indicating significant perturbation of ITIC 4F molecules by the CHP:CI thin film. This effect was more pronounced than that observed with the Y6 introduced film (Fig. 2d–f)." However, the provided figures do not show that the ITIC 4F system yields the strongest CD signal at either low or high concentration.

Response:

In the previous analysis, the CD data were compared without including the absorbance spectra of the acceptors, which led to ambiguity in interpreting how the CD response changes upon acceptor incorporation. To address this issue, we have added absorbance data for the neat acceptors and directly

correlated the observed CD modifications with their corresponding absorption features, enabling a more reliable interpretation.

From the updated data set (Fig. 2 in revised manuscript), it is evident that films containing ITIC-4F exhibit a clear and distinct CD peak near 700 nm, which directly corresponds to the 0–0 transition observed in the absorption spectrum of a neat ITIC-4F film. In contrast, for films incorporating Y6 or $PC_{71}BM$, no additional distinct CD peaks can be identified that can be unambiguously assigned to the electronic transitions in their respective absorption spectra.

By jointly analyzing the CD spectral features of CHP:CI films with each acceptor, we clearly confirmed that the CD modification induced by ITIC-4F is more pronounced than that induced by Y6 or $PC_{71}BM$ (Fig. R7). Because the amount of added acceptor was very small, we focused on spectral changes or the appearance of additional CD peaks induced by the acceptor, rather than on the absolute CD intensity or the g_{abs} values themselves.

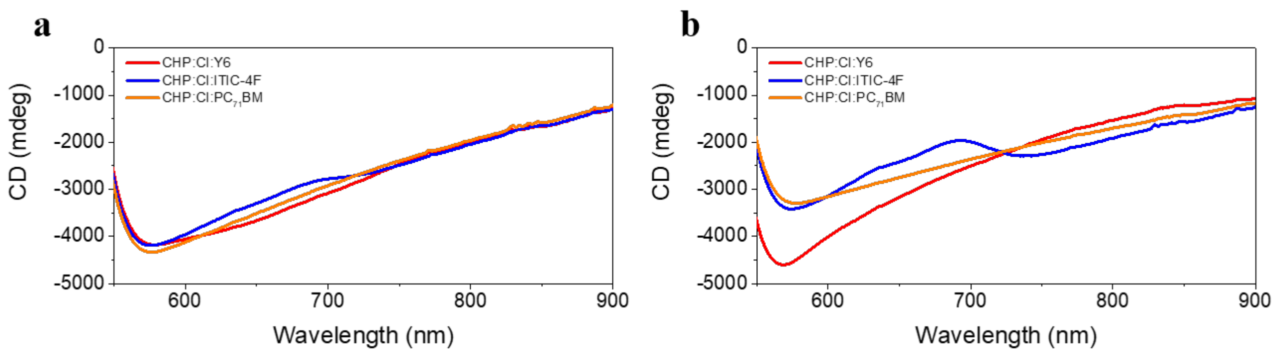

Fig. R7 CD spectra in the near-infrared region of CHP:CI blend films with different type of acceptors. a, CD spectra of 1 wt% introduction of Y6, ITIC-4F, and $PC_{71}BM$ into the CHP:CI blend. **b**, CD spectra of 5 wt% introduction of Y6, ITIC-4F, and $PC_{71}BM$ into the CHP:CI blend.

Comment 5: In Fig. 1a, the device electrode is labeled as Al, whereas the Methods section describes it as Ag. Please verify and correct the device structure accordingly.

Response:

For typical CP-OPD devices, Al was used as the electrode. However, for the large-area image sensor shown in Figure 5, a bilayer electrode structure was employed, consisting of 100 nm of Al followed by an additional 100 nm of Ag. The Ag overlayer was introduced to suppress electrode oxidation in large-area devices. We have clarified this point and corrected the corresponding description in the revised manuscript to avoid confusion.

Then, a 14 nm thick layer of C_{60} and 100 nm thick layer of Al was thermally evaporated under vacuum (<10^{-6} mbar). An additional Ag electrode (100 nm) was deposited on top to suppress oxidation of the device.

Comment 6: The authors did not include the [M] aza[6]H system in their core investigations on circularly polarized light detection and mechanism analysis. This omission leaves a critical gap in comparative evidence. It is recommended to supplement the study with experiments on the [M] aza[6]H system, which would help strengthen the central argument regarding the role of molecular chirality in the observed phenomena.

Response:

As the reviewer suggested, we newly included the investigations on CP-OPDs with a different enantiomer ([*M*]-aza[6]H) (Supplementary Fig. 2 in revised SI). Additional device experiments using the CHP:[*M*]-aza[6]H system confirmed that the device behaviours are the same as those observed for the CHP:[*P*]-aza[6]H system, which we believe strengthens our core argument and proposed mechanism. Together with the newly updated device experimental data obtained with CHP:[*M*]-aza[6]H blends, we have updated TPC data with CP-OPDs fabricated with an opposite enantiomer ([*M*]-aza[6]H).

Regarding TPC measurements, as opposed to CP-OPDs with [*P*]-aza[6]H, all devices exhibited a faster current decay under LCP excitation and a slower decay under RCP excitation. The fitting parameters for the TPC decay curves were summarized in Supplementary Table 3 in revised SI, with the lifetimes depending on the handedness of CP light (τ_R, τ_L) and the dissymmetric factor for the lifetime (g_τ), defined by $(\tau_R - \tau_L)/(\tau_R + \tau_L)$, as same shown in Fig. 3e. Similarly to the trend from CP-OPDs with [*P*]-aza[6]H, τ_L remained relatively constant across different acceptors, while τ_R showed significant variation depending on the acceptor type. This observation supports our interpretation that carrier extraction dynamics depend strongly on the polarization state of CP light by asymmetric trap dynamics. Specifically, under RCP illumination, the shorter optical penetration depth enhances electron trapping by the added acceptors, leading to the strongest suppression of charge extraction in the CP-OPD.

Consequently, we newly added the discussion and updated data, as follows:

(Page 12 in revised manuscript)

To ensure the reliability of the observed trend, we also conducted TPC measurements using the opposite enantiomer of aza[6]H incorporated into a chiral matrix (CHP:[*M*]-aza[6]H). As a result, the response to polarization was reversed upon using the opposite enantiomer, while the same trend was consistently observed upon addition of the acceptor (Supplementary Fig. 20).

(Page S3 in revised SI)

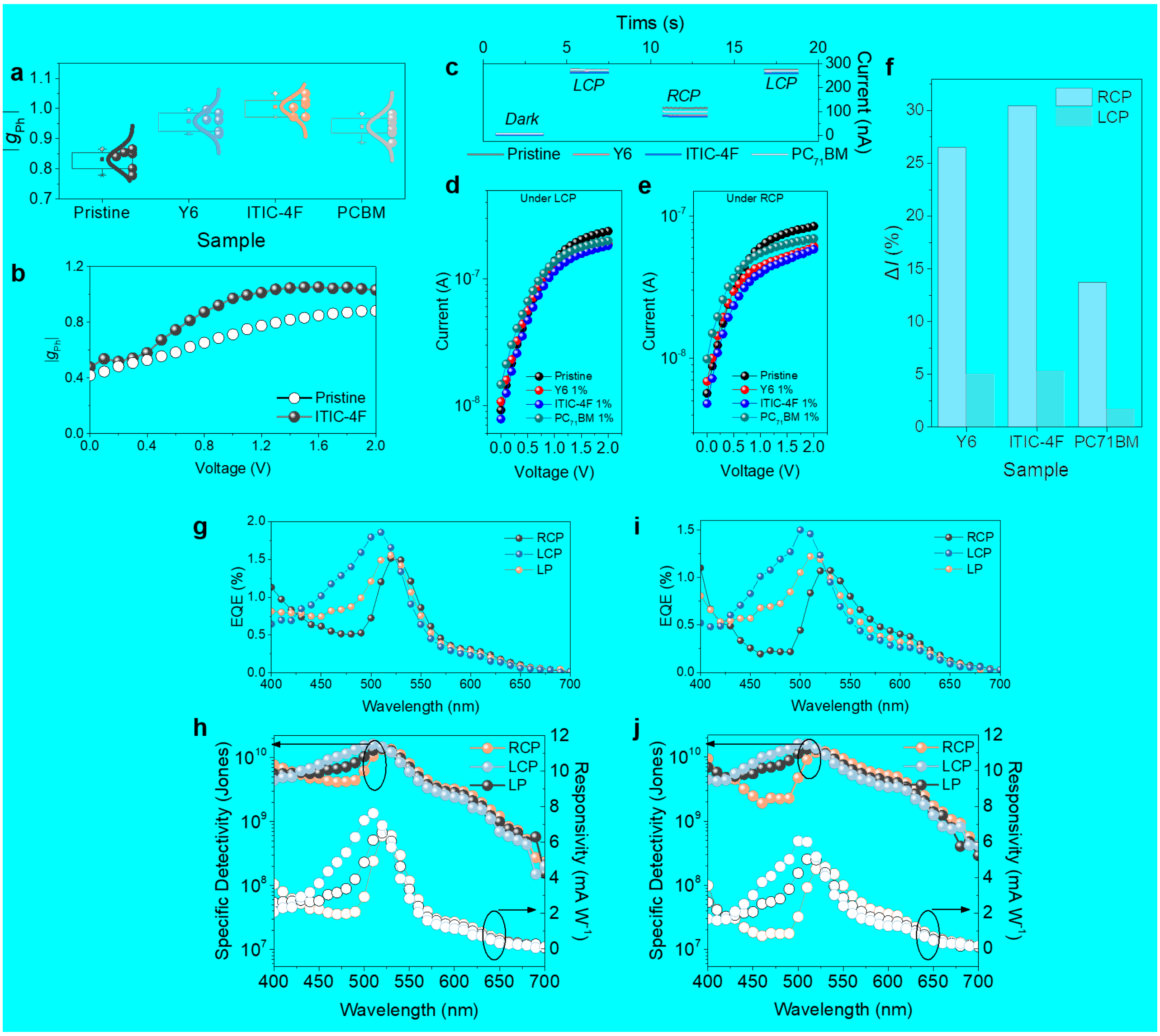

Supplementary Figure 2| g_{Ph} trends and device characteristics of CP-OPDs by using CHP:CI ([*M*]-aza[6]H) matrices, without acceptor or with acceptors. a, Statistics of g_{Ph} for each type of CP-OPDs under 470 nm CP light (23.6 mW cm^{-2}). **b**, Voltage-dependent g_{Ph} for the pristine and the ITIC-4F-incorporated CP-OPD. **c,** Time-dependent CP light detection characteristics for each type of device. **d,e,** *I*–*V* curves of each CP-OPDs under LCP (**d**) and RCP illumination (**e**). **f,** Photocurrent difference under RCP and LCP compared to those from the pristine device. **g–j,** EQE, Spectral *R*, and D^* of pristine CP-OPDs (**g,h**) or ITIC-4F-introduced CP-OPDs (**i,j**) under the linear polarized (LP), RCP, and LCP excitation.

(Page S21 in revised SI)

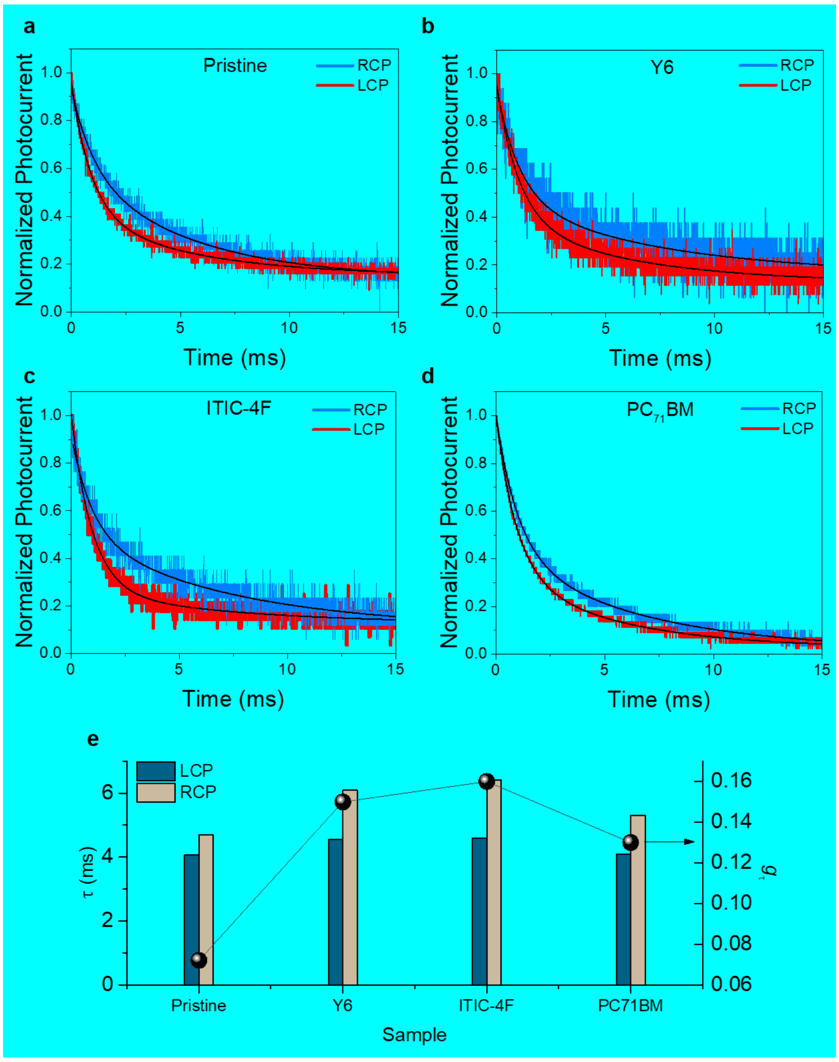

Supplementary Figure 20| TPC measurements with a different enantiomer of aza[6]H molecule. Polarization-dependent TPC decay curves of CP-OPDs without the acceptor (**a**) or with the introduction of Y6 (**b**), ITIC-4F (**c**), and $PC_{71}BM$ (**d**). Devices with the configuration of ITO/PEDOT:PSS/CHP:CI ([*M*]-aza[6]H)/C_{60}/Al were fabricated and measured under CP illumination at a wavelength of 470 nm. Fitted parameters for TPC analysis are shown in Supplementary Table 3.

We further performed additional PPPc measurements using CHP:CI films incorporating the opposite enantiomer, [*M*]-aza[6]H (Fig. R8). By comparing a pristine CP-OPD with a CP-OPD containing an acceptor, we found that acceptor-introduced devices display an enhanced polarization-dependent difference in ΔJ (i.e., $\Delta J_{IR,LCP}-\Delta J_{IR,RCP}$). This also verifies the asymmetric-trapping mechanism proposed in this work.

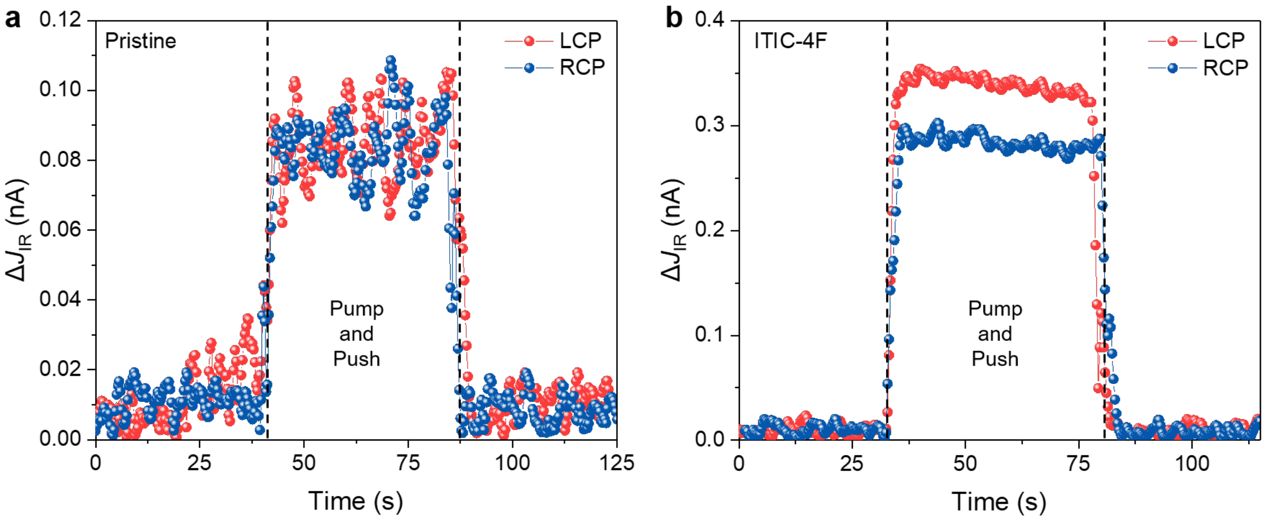

Fig. R8 PPPc measurements for CP-OPDs using an opposite enantiomer ([*M*]-aza[6]H). ΔJ_{IR} responses in polarization-dependent cw-PPPc measurement for pristine (**a**), ITIC-4F-introduced CP-OPDs (**b**).

Reviewer #3

In this work, the authors reported the trap-assisted circularly polarized organic photodetectors (CP-OPDs) based on π-conjugated polymer, helicene and small molecular dopant, such as PCBM, Y6 and ITIC-4F. By adopting the blend systems with small amount of these achiral acceptors, the authors achieved asymmetric trap filling that depends on the handedness of circularly polarized light. The modulated trap dynamics further enhance the CPL selectivity during charge extraction. Moreover, the authors fabricated a 4096-pixel large-area organic CP imaging system and demonstrated its potential for real-time visual encryption. Overall, this manuscript is well-organized, and the conclusion is strongly supported by the data. Therefore, I recommend to accept this work after addressing the below minor issues.

Response:

Thank you for your valuable comments. We are deeply grateful to the reviewer for the constructive and thoughtful comments, which were very helpful for improving our manuscript.

Comment 1: Why are different excitation wavelengths used (405 nm for PPPc experiments vs. 470 nm for CPL detection)? Could this affect the correlation between the proposed trap mechanism and CPL performance?

Response:

Owing to the higher signal-to-noise ratio achievable with the 405 nm laser compared to the available setup near 470 nm, PPPc experiments were primarily conducted using 405 nm excitation. Although there is a difference between the excitation wavelength used in the PPPc measurements and the wavelength region used for CP detection, we assume that the incorporation of 1% acceptor primarily introduces trap states that induce asymmetric trap behavior, which in turn governs the CP detection performance.

To validate that the observed asymmetric trap behavior from PPPc measurements indeed affects CP selectivity performance, we additionally characterized the CP selectivity performance of CP-OPDs under 405 nm excitation, as shown in Fig. R9. Although the absolute g_{Ph} values are much lower than those obtained under 470 nm excitation, mainly due to the intrinsically lower g_{abs} at 405 nm, the overall trend remains consistent. Taken together with the electron trap density (Supplementary Fig. 17 in revised SI) and differential optical penetration depth at 405 nm depending on the polarization of light (Supplementary Fig. 21 revised SI), the consistency between the PPPc results and CP detection performance at the same excitation wavelength indicates that asymmetric trap behavior is still the dominant mechanism responsible for the enhancement of g_{Ph}.

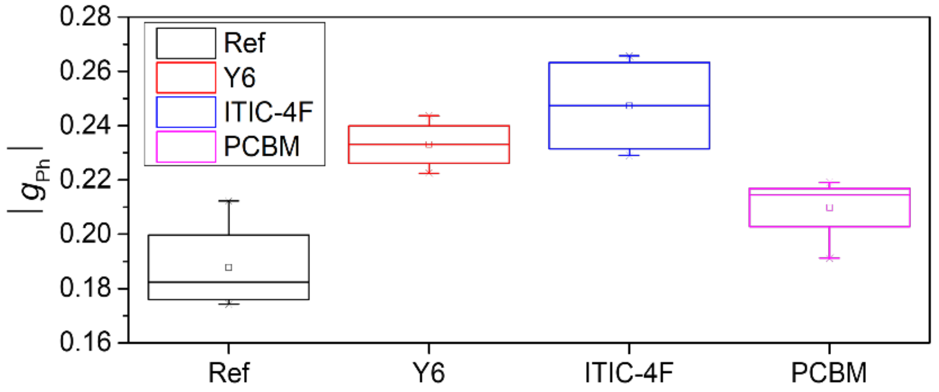

Fig. R9 Absolute g_{Ph} of CP-OPDs fabricated with the different type of acceptors under 405 nm. The thickness of the chiral active layers was kept same with devices characterized under 470 nm.

Comment 2: As PPPc only measures trapped carrier concentration, it remains unclear whether the observed discrepancy arises from differences in carrier generation or trap capture probability.

Response:

PPPc spectroscopy utilizes two laser beams: a visible pump that photogenerates charge carriers and a sub-bandgap infrared (IR) push that interacts with charges trapped in the space-charge region. In our continuous-wave PPPc (cw-PPPc) measurements, the optical pump first creates band-edge carriers (generating J_{Pump}), a fraction of which become trapped during or after photoexcitation. The IR push beam, with photon energies below the bandgap, is subsequently absorbed by some of these trapped carriers, promoting them back into extended band states (Ref: *Nat. Commun.* 2023, 14, 8000). These IR-released carriers, which might otherwise recombine, contribute to an additional photocurrent in the device (ΔJ_{IR}) (Fig. R10). Consequently, the magnitude of the IR-induced photocurrent provides a direct measure of the trapped carrier population in the device.

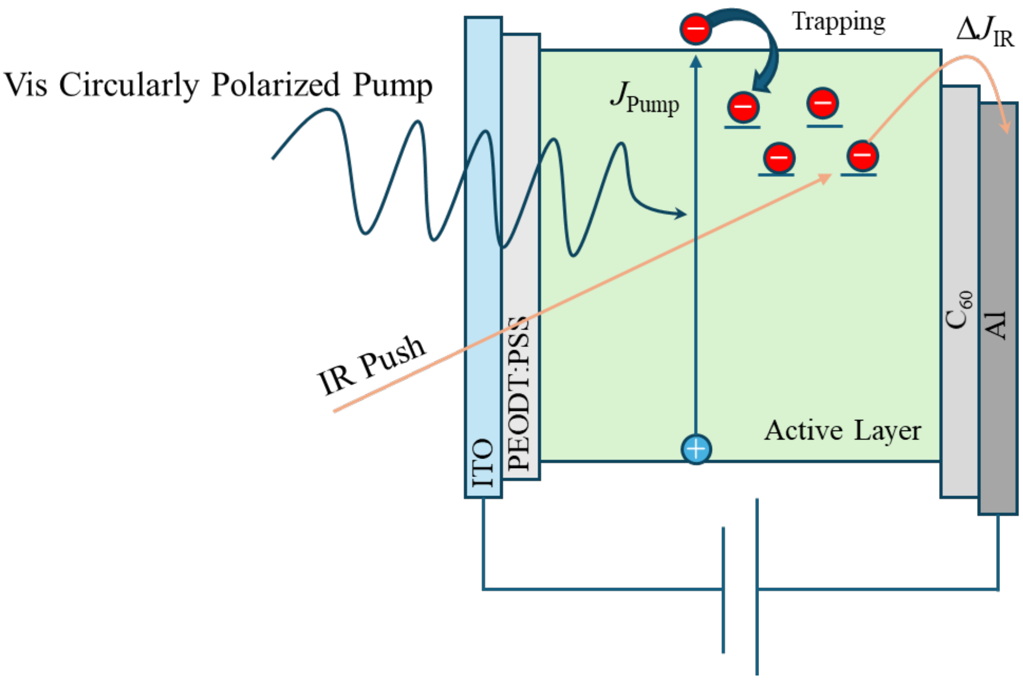

Fig. R10 Schematic representation of PPPc working mechanism in device under operando working conditions.

Thus, the observed discrepancy in ΔJ_{IR} originates from relative differences in the concentration of trapped carriers rather than from variations in carrier generation.

Our key observations are that the difference in ΔJ_{IR} between left- and right-circularly polarized (LCP and RCP) excitation is maximized upon introducing the acceptor. These results indicate that trapping event becomes strongly dissymmetric under CP excitation when the small amount of acceptors is introduced. This dissymmetric trapping effect, in turn, has a significant impact on the device's CP selectivity (as shown in Fig. 4 in manuscript).

Comment 3: The DOSY experiments were performed in solution, while the devices are based on thin films. Could this state difference impact the correlation between the observed intermolecular interactions and the CPL device performance?

Response:

Please see our review to comment 4 of reviewer 1.

Comment 4: Please compare the device performance with that of state-of-the-art photodiode-based CP-OPDs to clarify the advantages.

Response:

As suggested by the reviewer, we have updated Supplementary Table 2 in the revised SI to provide a more comprehensive comparison of key performance metrics, specifically detectivity and

responsivity, between our CP-OPD and state-of-the-art CP-OPDs, including both photodiode-type and transistor-type devices, as follows:

(Page S31 in revised SI)

Chiral Matrices	Absolute g_{abs}	Absolute g_{Ph}	V_d (V)	Detectivity (Jones)	Responsivity (AW^{-1})	Detecting λ (nm)	Device Type	Reference
aza[6]H	~10^{-3}	1.8	60	N/A	N/A	365	Transistor	8
CPDI-Ph	0.015	0.33	70	N/A	15.7	460	Transistor	9
F6BT	N/A	1.94	60	7.44×10^{7}	1.86×10^{-5}	450	Transistor	10
ClCPDI-Ph-CF	N/A	0.129	10	2.2×10^{16}	10^{3}	495	Transistor	11
Bis[60]PCBM enantiomer	0.0006	1.27	20	N/A	N/A	405	Transistor	12
3a-*P*/*M*	0.002	0.057	80	2.1×10^{10}	0.45	730	Transistor	13
Chiral naphthalene diimides	2.2×10^{-3}	0.05	30	3.9×10^{12}	314	385	Transistor	14
4CldiPDI	0.005	0.16	80	4.7×10^{11}	16.5	488	Transistor	15
C13P3.75:S6N	0.2	0.58	80	5×10^{11}	3.88	405	Transistor	16
NTPH-P:DPA	1.5×10^{-3}	0.24	60	1.33×10^{9}	0.233	556	Transistor	17
Metal organic framework (*R*-MeBINOL-PorDC)	N/A	4.3×10^{-4}	2	N/A	N/A	450	Photoconductor	18
P3CT:BN	N/A	0.1	5	N/A	N/A	375	Photoconductor	19
chiral covalent organic frameworks (CityU-7 and CityU-8)	2.3×10^{-4}	0.95	0.1	5.5×10^{8}	1	405	Photoconductor	20
chiral silver nanoclusters (Ag_7(R/S-DMA)$_2$(dpppy)$_3$ $(BF_4)_3$)	N/A	0.18	1	1.00×10^{9}	5.92	520	Photoconductor	21
Chiral fluorene copolymer	0.06	0.017	0	N/A	N/A	543	Diode	22
(*S*,*S*)-ProSQ-C6:PCBM	0.09	0.1	0	N/A	N/A	543	Diode	23
F8T2:aza[6]H	0.03	0.41	0	N/A	N/A	510	Diode	24
DPP6T:PCBM	0.02	0.17	0	2.64×10^{10}	0.044	606	Diode	25
Chiral polythiophene nanowires	0.0016	0.047	0.4	1.25×10^{11}	0.123	560	Diode	26
ITIC:R5011	0.09	0.113	2	1.07×10^{13}	0.329	700	Diode	27
PM6:chiral BTP-4Cl	0.02	0.03	0	3×10^{11}	0.4	830	Diode	28
PM6:chiral BTP-4F	0.015	0.016	0	N/A	0.4	830	Diode	29
Chiral Polyacetylene	2.25×10^{-3}	0.08	0	3.15×10^{7}	0.18	365	Diode	30
PCDTPT:limonene	N/A	0.18	0	1.5×10^{12}	6	532	Diode	31
PCPDTTBTT:S5011	1.21	1.16	1	1.5×10^{11}	N/A	670	Diode	32
PCPDTTBTT:S5011:N2200	1	0.53	5	2.1×10^{11}	0.75	730	Diode	33
(+)/(−)-2NDI	0.06	1.67	50	8.25×10^{10}	13.76	450	Transistor	34
(*S*) or (*R*)-P(NDI2MH-T2)	1.38×10^{-2}	0.043	70	1.1×10^{12}	92	670	Transistor	35
F8T2:aza[6]H	0.7	1.19	1.5	1.01×10^{10}	3.84×10^{-3}	470	Diode	This work

The detectivity and responsivity of our device are relatively modest compared to previously reported CP-OPDs. This is primarily attributed to the bilayer device architecture incorporating a thick active layer and C_{60}. Importantly, the primary objective of this study is to introduce and demonstrate a new and effective strategy for enhancing photocurrent asymmetry (g_{Ph}) (one of the most critical performance metrics in chiral optoelectronic devices) at a fixed system without the need to increase g_{abs}. Thus, we focused on suggesting a new understanding of how trap-mediated charge collection governs g_{Ph} at the device level. Further optimization of conventional performance metrics, such as detection sensitivity and responsivity, within the current device architecture will be the subject of our future studies.

Rather than relying on increasing the intrinsic chiroptical response (g_{abs}) or redesigning molecular structures to boost g_{Ph}, we reveal that intentionally introduced traps can induce asymmetric trap dynamics that strongly amplify g_{Ph} at the device level. This new insight fundamentally distinguishes our approach from previously reported strategies. As a result, our device achieves among the highest g_{Ph} reported to date for *diode-type CP-OPDs operating at very low bias voltages*, underscoring the unique advantage and significance of our methodology.

Comment 5: The latest progress of CP-OPDs should be included (DOI: 10.1016/j.matt.2025.102371; 10.1038/s41528-025-00443-2).

Response:

As the reviewer suggested, we included the references in Fig. 11 and Supplementary Table 2 in our revised SI as follows:

(Page S31 in revised SI)

(+)/(−)-2NDI	0.06	1.67	50	8.25×10^{10}	13.76	450
(*S*) or (*R*)-P(NDI2MH-T2)	1.38×10^{-2}	0.043	70	1.1×10^{12}	92	670

Comment 6: Please address whether the strategy of enhancing gph via the trap strategy sacrifices other device performance metrics (e.g., response speed, detectivity, stability) and provide a balanced assessment of the strategy in the manuscript.

Response:

As the reviewer suggested, to clearly show how other device performance metrics behaves with the introduction of acceptor, we conducted additional experiments and presented the complete device comparison between the pristine and acceptor-introduced CP-OPDs (Supplementary Fig. 8 in revised SI). As shown, the detectivity, responsivity, response speed, and LDR of acceptor-introduced CP-OPDs have similar or slightly lower performance level of those obtained from pristine CP-OPD without an acceptor. This is because the intentionally introduced trap sites slightly reduce charge extraction efficiency. In particular, the response speed of the acceptor-doped CP-OPD was slower than that of the pristine CP-OPD, indicating that the additional trap sites created by the added acceptor slow down the device response. Meanwhile, the acceptor-introduced CP-OPDs exhibited slightly lower noise current than pristine CP-OPDs, so the devices did not suffer a significant loss in detectivity.

To provide a balanced assessment of our strategy, we added the comparative discussion on the other device performance metrics between the pristine and the acceptor-introduced CP-OPDs as follows:

(Page 6 in revised manuscript and page S9 in revised SI)

Other figures of merit of acceptor-introduced CP-OPDs, such as D^*, R, response speed, and linear dynamic range (LDR) were maintained similarly or slightly decreased compared to those obtained from pristine CP-OPDs without the addition of acceptors (Supplementary Fig. 8). The reductions can be explained by a decrease in charge extraction efficiency caused by traps induced by the acceptor. The pristine CP-OPDs exhibited D^* values of 8.42×10^9 Jones and 3.11×10^9 Jones, together with R of 4.65 mA W^{-1} and 1.72 mA W^{-1} under 500 nm RCP and LCP illumination, respectively. Compared with the ITIC-4F–introduced CP-OPDs, the pristine devices showed slightly higher EQE, R, and faster response times. However, the introduction of ITIC-4F led to a pronounced enhancement in CP selectivity. In addition, the ITIC-4F-introduced CP-OPDs exhibited a slightly higher D^* under RCP illumination than the pristine CP-OPDs, which was attributed to their slightly lower noise current. Although a minor trade-off in overall photodetector performance was observed, the incorporation of ITIC-4F resulted in a substantially enhanced polarization-dependent differential response.

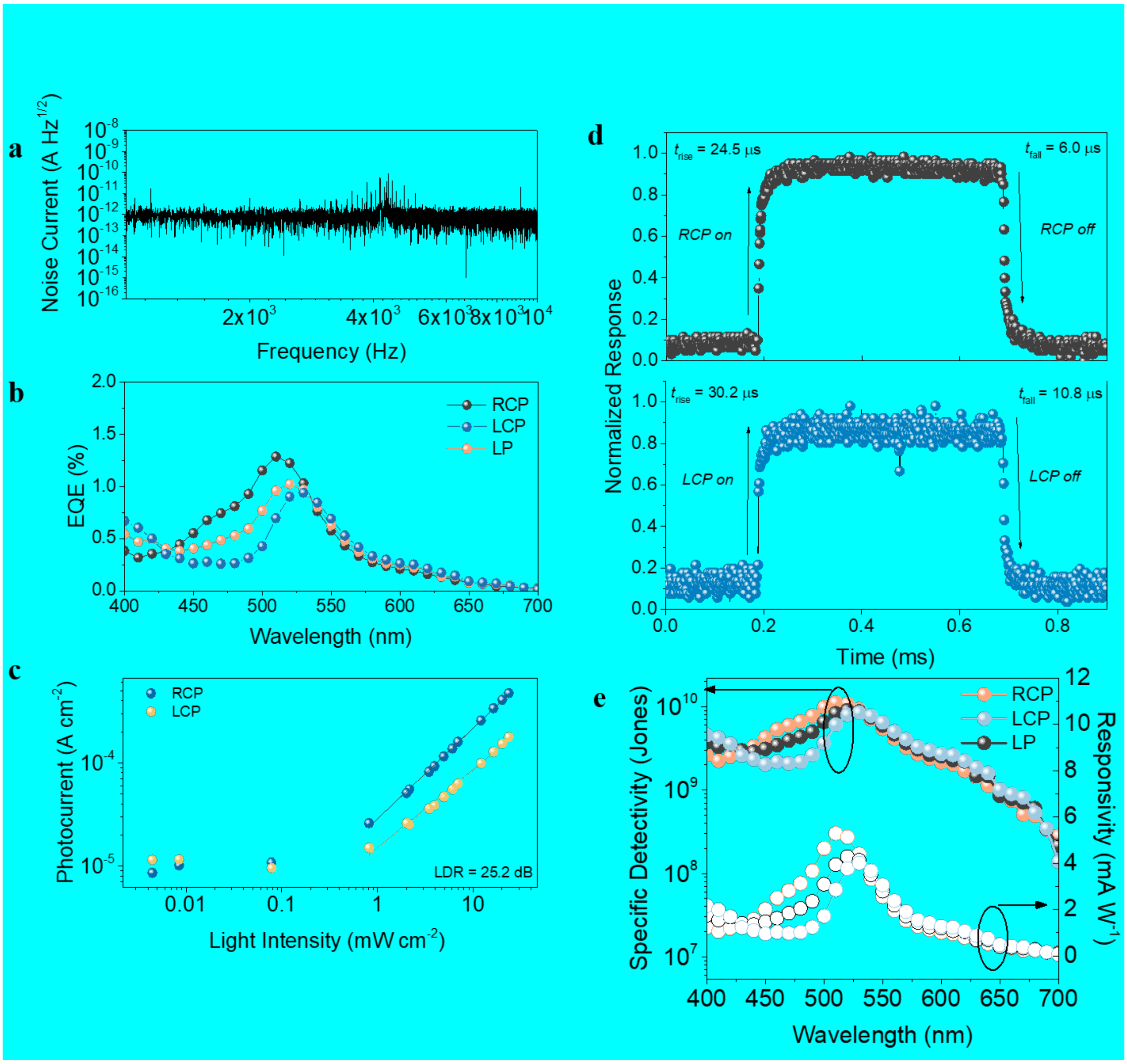

Supplementary Figure 8| Photo-response characteristics of pristine CP-OPDs. a, Noise current spectral density. **b**, EQE spectra of CP-OPD with the different polarization of light. **c**, LDR under CP light. **d**, Rise and fall time under RCP (top) and LCP light excitation (bottom). **e**, Spectral *R* and D^* of ITIC-4F-introduced CP-OPDs under the linear polarized (LP), RCP, and LCP excitation.

Comment 7: There are spelling errors in the manuscript (e.g., "afftect" should be "affect"), and it is recommended that the authors undergo professional English language proofreading before submission.

Response:

Thank you for your careful comment. Throughout the paper, we reviewed and corrected spelling errors.
